# Alizarin, a nature compound, inhibits the growth of pancreatic cancer cells by abrogating NF-κB activation

**DOI:** 10.7150/ijbs.70567

**Published:** 2022-03-28

**Authors:** Zihang Xu, Yifei Hou, Chunpu Zou, Haibin Liang, Jiasheng Mu, Xiaoning Jiao, Yangzhuangzhuang Zhu, Lin Su, Mingxi Liu, Xiao Chen, Chunmei Qian, Xiandan Zhu, Wei Gong, Qian Dong, Fei Zhang

**Affiliations:** 1 Shanghai Key Laboratory of Health Identification and Assessment, School of Basic Medical Science, Shanghai University of Traditional Chinese Medicine, Shanghai, 201203, China.; 2 Department of General Surgery, Xinhua Hospital affiliated to Shanghai Jiao Tong University School of Medicine, Shanghai, 200092, China; 3 Experimental Center for Science and Technology, Shanghai University of Traditional Chinese Medicine, Shanghai, 201203, China

**Keywords:** Nature compound, Alizarin, Pancreatic cancer, NF-κB signal, Gemcitabine

## Abstract

The current performance of nature compounds in antitumor field is gradually attracted more and more attention, we discovered a nature active ingredient alizarin possess potent natural reductive NF-κB activity to against pancreatic cancer. However, the preclinical pharmacology and therapeutic effect, and the underlying mechanisms of alizarin in inhibiting pancreatic cancer are still unclear. After high-throughput screening, this is the first report that alizarin can induce a potent inhibitory effect against pancreatic cancer cells. Alizarin induced cell cycle arrest and promoted cell apoptosis by inhibiting TNF-α-stimulated NF-κB activity and nuclear translocation, and inactivated its related TNF-α-TAK1-NF-κB signaling cascade followed by downregulation of NF-κB target genes involved in cell apoptosis (Bcl-2, Bcl-xL, XIAP) and in the cell cycle and growth (cyclin D, c-myc). Due to the abrogation of NF-κB activity, combination of alizarin and gemcitabine exerted a better inhibitory effect on pancreatic cancer. In summary, natural component alizarin, inhibited cell proliferation and induced apoptosis *in vitro* and *in vivo* through targeting of the NF-κB signaling cascade with minimal toxicity, which combine with gemcitabine, can significantly enhance the antitumor capability, playing a synergistic effect. Therefore, alizarin may play a role in reversing gemcitabine resistance caused by overactivated NF-κB in clinical application in the future.

## Introduction

Pancreatic cancer is a common type of digestive system tumor with high malignancy and poor prognosis, with a 5-year survival rate of approximately 10~25% 1. Due to the rapid progression, high aggressiveness and metastasis ability of pancreatic cancer, the incidence and mortality of pancreatic cancer have been increasing year by year globally 2, 3. Based on the latest data on the global cancer burden for 2020 from the International Agency for Research on Cancer (IARC), pancreatic cancer ranks 7^th^ in global tumor mortality rate, while in China, the incidence and mortality of pancreatic cancer are both in the top 10, ranking 8^th^ and 6^th^, respectively 1. Because of the deep anatomical location of the pancreas, early clinical symptoms of pancreatic cancer are not always easy to recognize. More than 85% of patients have local infiltration or metastasis of cancer cells at diagnosis, and the prognosis is poor 4. At present, surgery combined with chemotherapy is the dominant traditional treatment for pancreatic cancer patients. However, one of the other important characteristics of this disease is severe drug resistance, which inevitably leads to treatment failure 5. For example, gemcitabine (Gem) is the first-line and standard chemotherapy for advanced pancreatic cancer, but drug resistance often causes treatment to be ineffective 6. Therefore, developing new potent and safer agents for the treatment of pancreatic cancer is warranted. In recent years, nature compounds have attracted increasing attention due to its potential antitumor capability and minimal toxicity 7.

Radix rubiae (Qian cao) is a kind of Chinese herbs that has been very commonly used clinically in China for thousands of years and was first documented in the classic ancient book *Shennong's Classic of Material Medical* in the Han Dynasty. Radix rubiae, the root and rhizome of Rubiaceae (Rubia cordifolia L.), are excavated in spring and autumn, the sediment is removed, and the substance is dried. The traditional Chinese medicine (TCM) medicinal effects of Radix rubiae mainly include cooling blood, promoting blood circulation, removing blood stasis, and easing menstruation. Therefore, according to the basic theory of TCM, Radix rubiae can treat hematemesis, epistaxis, hematochezia, blood collapse, blood in the urine, amenorrhea-related abdominal pain, bruising, blood stasis, and so on, in clinical practice. In addition, pharmacological studies have shown that the active ingredients of Radix rubiae, including a variety of hydroxyl anthraquinone derivatives, alizarin, purpuro-xanthin, purpurin, pseudopurpurin, munjistin, rubia, and ruberythric acid, possess anti-inflammatory, anticancer, antioxidant, hemostatic, antibacterial and other pharmacological characteristics 8.

In our current study, we identified alizarin as one of the most active anthraquinone components of Radix rubiae by a luciferase-based high-throughput screening system we established 9; alizarin exhibited potent anti-pancreatic cancer capability. In this study, it was revealed for the first time that alizarin inhibited the growth of pancreatic cancer cells and induced cellular apoptosis by blocking the nuclear translocation of NF-κB and downregulating the related TNF-α-TAK1-NF-κB signaling cascade. According to multiple studies, the exceptional increase in NF-κB is one of the major mechanisms causing Gem resistance in the treatment of pancreatic cancer 10, 11. In our study, we demonstrate for the first time that alizarin combined with Gem has a synergistic effect in orthotopic pancreatic cancer mice. Moreover, alizarin showed minimal cytotoxicity against normal cells and had almost no overt side effects in mice, highlighting the potential of alizarin as a safe and potent candidate and warranting its development as a complementary and alternative therapy for pancreatic cancer.

## Materials and methods

### Cell lines and cell culture

The cell lines HPDE, 293T, PANC-1, MIA PaCa-2, Capan-1, SW1990 and BxPC3 were purchased from the Cell Bank of the Chinese Academic of Science (Shanghai, China). BxPC3 cells were cultured in RPMI-1640 (Gibco, Gran Island, NY) supplemented with 100 U/mL penicillin-streptomycin (Hyclone, Logan, Utah) and 10% fetal bovine serum (Gibco, Gran Island, NY). 293T, Capan-1, SW1990, PANC-1, MIA PaCa-2, cells were maintained in DMEM (Gibco, Gran Island, NY), which contained 100 U/mL penicillin-streptomycin and 10% fetal bovine serum. HPDE cells were cultured in K-SFM medium (Gibco) containing 10% FBS and 1% epidermal growth factor. All the cell lines were incubated at 37^◦^C and 5% CO_2_.

### Drugs, reagents and antibodies

Alizarin and the nature compound library were kindly provided by China National Compound Resource Center, and the compounds were dissolved in dimethyl sulfoxide (DMSO, Sigma-Aldrich, USA) to prepare a stock solution, and the purity was analyzed by HPLC to be at least 95%. Red blood cell lysis buffer (68027757) was purchased from Biosharp (Shanghai, China); trypsin containing 0.25% EDTA (25200072), GeneChip IVT labeling kit (901609) were obtained from Thermo Fisher Scientific (Waltham, MA); Enhanced CCK-8 kit (C0042), crystal violet staining solution (C0121), cell cycle and apoptosis detection kit (C1052), Trizol reagent (R0016), SDS-PAGE protein loading buffer (P0015L), GAPDH (AF0006), Hsp90 (AF1378), Histone H3 (AF0009) were purchased from Biyuntian Biotechnology Co., Ltd. (Shanghai, China); Hoechst 33342 staining solution (C0031) was purchased from Beijing Soleibao Technology Co., Ltd. (Beijing, China); TAK1 (ab109526), TAB1 (ab76412), IKKα/β (ab178870), p-IKKα/β (ab194528), NF-κB p65 (ab16502), p-NF-κB p65 (ab76302), ERK (ab32537), p-ERK (ab201015), IkB-α (ab32518), p-IkB-α (ab92700), p38 (ab170099), p-p38 (ab178867), JNK (ab124956), p-JNK (ab76572), c-myc (ab32072) and kit for determination of mitochondrial membrane potential (ab113852) were purchased from Abcam (Cambridge, MA) and used for Western blotting, Immunofluorescence, co-immunoprecipitation analysis or detection of mitochondrial membrane potential; XIAP (14334), Cyclin D1 (55506S), Cyclin B1 (12231T), Bcl-xl (2762S), cl-PARP (9548T), cl-Caspase-3 (9664T), PCNA (13110S) and 488 conjugated anti-rabbit antibody (4412) were purchased from Cell Signaling Technology (Danvers, MA) and used for Western blotting, immunohistochemistry or immunofluorescence analysis.

### High-throughput screening assay

Based on the previous work of our lab, a luminescence-based cell viability assay was designed to identify natural products that can inhibit the viability of pancreatic cancer cells 9. Pancreatic cancer cells were treated with 2951 candidate products in the nature compounds library. After a total of 24 hours of incubation, luciferase and its substrate were added to the medium. In the catalysis process of luciferase, O_2_ and ATP produced by tumor cells were required, and then interacted to produce bioluminescence. The proportion of metabolically active cells were showed by quantitative detection of tumor cell lysis by ATP, and then the nature compounds that have inhibitory effects on pancreatic cancer cells were screened out.

### Gene ontology and pathway enrichment analysis for alizarin

The DAVID Bioinformatics Resources was used for gene ontology (GO) function and KEGG pathway enrichment analysis. According to the P value, the biological process in GO function analysis and the enrichment result of KEGG pathway are analyzed, and the bubble chart and histogram are produced through the bioinformatic online platform (http://www.bioinformatics.com.cn/) for visualization 12.

### Cell viability assay

Pancreatic cancer cells in the logarithmic growth phase were seeded into a 96-well plate at a density of 5×10^3^ cells/well. After the cells adhered, alizarin was configured at a concentration of 0, 5, 10, 20, 40, and 80 μM. The corresponding drug-containing solution was added to the cells, and each group was set up with 5 multiple wells, cultured for 24h, 48h, 72h, then added CCK8 reagent, placed in the incubator and incubated for 2h, then the OD value was measured with a microplate reader (Molecular, USA).

### Clone formation experiment

The pancreatic cancer cells in the logarithmic growth phase were prepared to the single cell suspension, and cells were inoculated into dishes containing different concentrations of alizarin (0, 5, 10, 20 μM) or Gem (2 μM) culture solution for 48h. After treatment, the cells were allowed to form cell colonies for another 14 days. Cell colonies were then fixed with 4% paraformaldehyde, stained with crystal violet staining solution for 10~30 minutes. The clone formation rate was calculated by: clone formation rate = number of clones/inoculated cells number×100%.

### Cell cycle analysis

The pancreatic cancer cells treated with the culture medium containing different concentrations of alizarin were prepared into a single cell suspension, centrifuged at 2000 rpm for 5 minutes, then the cells were resuspended and washed with pre-cooled PBS, and the supernatant was removed by suction. The cells were fully resuspended in 1mL PBS to form a single cell suspension which was shaken while slowly adding 3mL of pre-cooled absolute ethanol dropwise to the final concentration of 75%, and it was leaved at 4^◦^C overnight (18~24h) for cell fixation. The fixed cells were washed twice with pre-cooled PBS, and the supernatant was removed by centrifugation at 2000 rpm for 5 minutes. The cells were resuspended in 200 μL PBS and 20 μL 50 μg/mL RNase. The cells were incubated in a water bath at 37^◦^C for 30 minutes, and then 20 μL of PI was added to a final concentration of 50 μg/mL. The cells were stained for 30 minutes at 4^◦^C in the dark, and detected by flow cytometer (Beckman Coulter, USA).

### Cell apoptosis

The Annexin V-FITC Apoptosis Detection Kit (BioVision) was used for the apoptosis analysis. Cells (5×10^5^) were exposed to different concentrations of alizarin for 48 hours. After resuspended in 5mL binding buffer, cells were incubated with Annexin V-fluorescein isothiocyanate (FITC) and PI. After 30 minutes incubation, cells were analyzed by fluorescence-activated cell sorting (FACS) by flow cytometer. Annexin V-FITC-stained only cells which indicated early apoptosis and cells with Annexin V-FITC- and PI double positive signals were combined for analysis.

### Hoechst Staining

Pancreatic cancer cells treated with culture solutions containing different concentrations of alizarin were prepared the single cell suspension which was fixed with acetic acid-ethanol and rinsed with PBS for 5 minutes. The cells were stained with Hoechst working solution for 15 minutes at room temperature. Then, the cells were rinsed with PBS three times for 5 minutes each; next, they were mounted with a 1:9 mixture of glycerol and PBS, and finally observed under a fluorescence microscope (Zeiss, Germany).

### Affymetrix geneChip human genome array

Total RNA were extracted from pancreatic cancer cells treated with alizarin or DMSO using Trizol reagent (Invitrogen, Carlsbad, CA) according to instructions of manufacturer, and digested with DNase I at 37^◦^C for 15 minutes to remove any contaminating DNA. The RNA was cleaned up with RNeasy Kit (Qiagen, Hilden, Germany) and the quantities and qualities were determined by spectrophometer and 1% formaldehyde denaturing gel electrophoresis. The samples with bright bands of ribosomal 28S to18S RNA in a ratio > 1.5:1 were used for microarray analysis. Affymetrix GeneChip® PrimeView™ Human Gene Expression Array, which contains more than 53,000 probe sets covering more than 36,000 transcripts and variants, which represent more than 20,000 genes, was used in microarray annalysis. Hybridization, data capture, and analysis were performed by CapitalBio Corporation (Beijing, China), a service provider authorized by Affymetrix Inc (Santa Clara, CA).

### Mitochondrial membrane potential detection

After pancreatic cancer cells were treated with alizarin, according to the instructions, TMRE was added to the cells and incubated at 37^◦^C for 20 minutes. TMRE staining was analyzed by microplate spectrophotometry at Ex/Em = 549/575 nm.

### Immunofluorescence (IF)

The cells were fixed with 4% paraformaldehyde for 20 minutes, then treated with 1% Triton X-100 to permeate for 20 minutes, and incubated with anti-NF-κB p65 antibody overnight at 4^◦^C. Then the cells were incubated with 488-conjugated anti-rabbit antibody for 30 minutes, and stained with 4',6-diamidino-2-phenylindole (DAPI) for 20 minutes, and images were obtained by Laser scanning confocal microscope (Oberkochen Zeiss, Germany).

### Animal experiment

The stable pancreatic cancer MIA PaCa-2-luc cell line expressing luciferase (luciferace, luc) was constructed by our lab. The pancreatic cancer MIA PaCa-2-luc cells were cultured to the logarithmic growth phase and then were digested with 0.25% trypsin, then were adjusted to 2×10^6^/100 μL with PBS. The 50 μL cell suspension was mixed with matrigel (Corning) in an equal volume of 1:1, and the mixture was injected into the pancreas of SCID mice. All mice were randomly divided into alizarin high (30 mg/kg) or low (10 mg/kg) concentration group, Gem (5 mg/kg) + alizarin (10 mg/kg) group and control group. Alizarin was injected into the tail vein of mice in the alizarin group every afternoon; Gem was given intraperitoneally every three days; the control group was injected with the same amount of PBS, and each group was administered once a day for 28 consecutive days.

On the 7, 14, 21, and 28 days after modeling, the mice were imaged with IVIS Lumina XR Imaging System (Perkin Elmer, Germany) to observe tumor growth. At the same time, the mice's physical signs such as activity, hair, claw nails, and body temperature were observed daily.

### Co-immunoprecipitation assay

Cells were lysed in HPO buffer containing protease and phosphatase inhibitors (50 mM Hepes pH 7.5, 100 mM NaCl, 50 mM KCl, 2 mM MgCl2, 1% Triton X-100, 0.5% NP-40, 5% glycerol) and were homogenized by ultrasonic treatment. Cell lysates were immunoprecipitated with a primary antibody conjugated with protein A conjugated agarose in 2% BSA. After incubating for 2.5 hours on a rotator at 4^◦^C, the beads were washed 3 times in the same buffer, and the purified protein was boiled in Laemmli final sample buffer to perform Western blotting with a secondary antibody specific for HRP.

### Western blotting analysis

The total protein of cells or tissues was extracted, and a standard curve was made by BCA quantitative method. 10% separating glue and 5% concentrated glue were configured. The loading solution was prepared, and electrophoresis and membrane transfer were performed after loading. After the transfer, the membrane was blocked with skim milk for 1.5 hours, and the primary antibody was added to incubate at 4^◦^C overnight, then TBST washed 3 times, next the secondary antibody was added to incubate at room temperature for 1.5 hours, and the membrane was washed 3 times with TBST. Finally, the images were used ECL chemiluminescence solution to develop in the Chemidoc Imaging System (Bio-Rad; Hercules, CA), and was ananlysed by image J software, then was compared the gray value of protein bands.

### Hematoxylin-eosin (H&E) staining

After 4 weeks of tail vein injection of high and low doses of alizarin and PBS, the mice were euthanized by overdose of 1% sodium pentobarbital, and the mouse tumor tissue was fixed by 4% paraformaldehyde, dehydrated by ethanol gradient, then transparented with xylene, and then placed in paraffin for embedding sections. After drying, the paraffin sections were deparaffinized to water. Stain with wood essence and eosin dye, and finally, the sections were mounted and placed under a microscope for observation.

### Immunohistochemical (IHC) staining

The mouse tumor tissues or patient tumor tissues were taken for dewaxing and hydration and then the non-specific protein was blocked by 5% goat serum. After the sections were incubated with primary and secondary antibodies and developed with 3.3'-diaminobenzidine and counterstained with hematoxylin. After dehydration and fixation, images were captured under light microscopy.

### Enzyme linked immunosorbent assay (ELISA)

Blood was taken from the eyeballs of the mice after anesthesia and the serum was separated, next the levels of alanine aminotransferase (ALT), aspartate aminotransferase (AST), creatinine (Cr) and creatine kinase (CK) in the serum were detected according to the manufacturer's instructions.

### Statistical analysis

All data are expressed as the mean values ± standard errors of at least three independent experiments. Statistical significance was calculated using the Mann-Whitney U test and a p value less than 0.05 was considered significant in all tests. Multiple comparisons were performed by one-way ANOVA with Tukey's comparisons-test. Univariate survival analysis was performed using the Kaplan-Meier test. All analyses were performed using SPSS software version 19.0 (SPSS Inc., Chicago, IL, United States).

## Results

### A luminescence-based high-throughput screening system identified that alizarin exhibits potent cytotoxicity against pancreatic cancer cells

**Figure 1:**
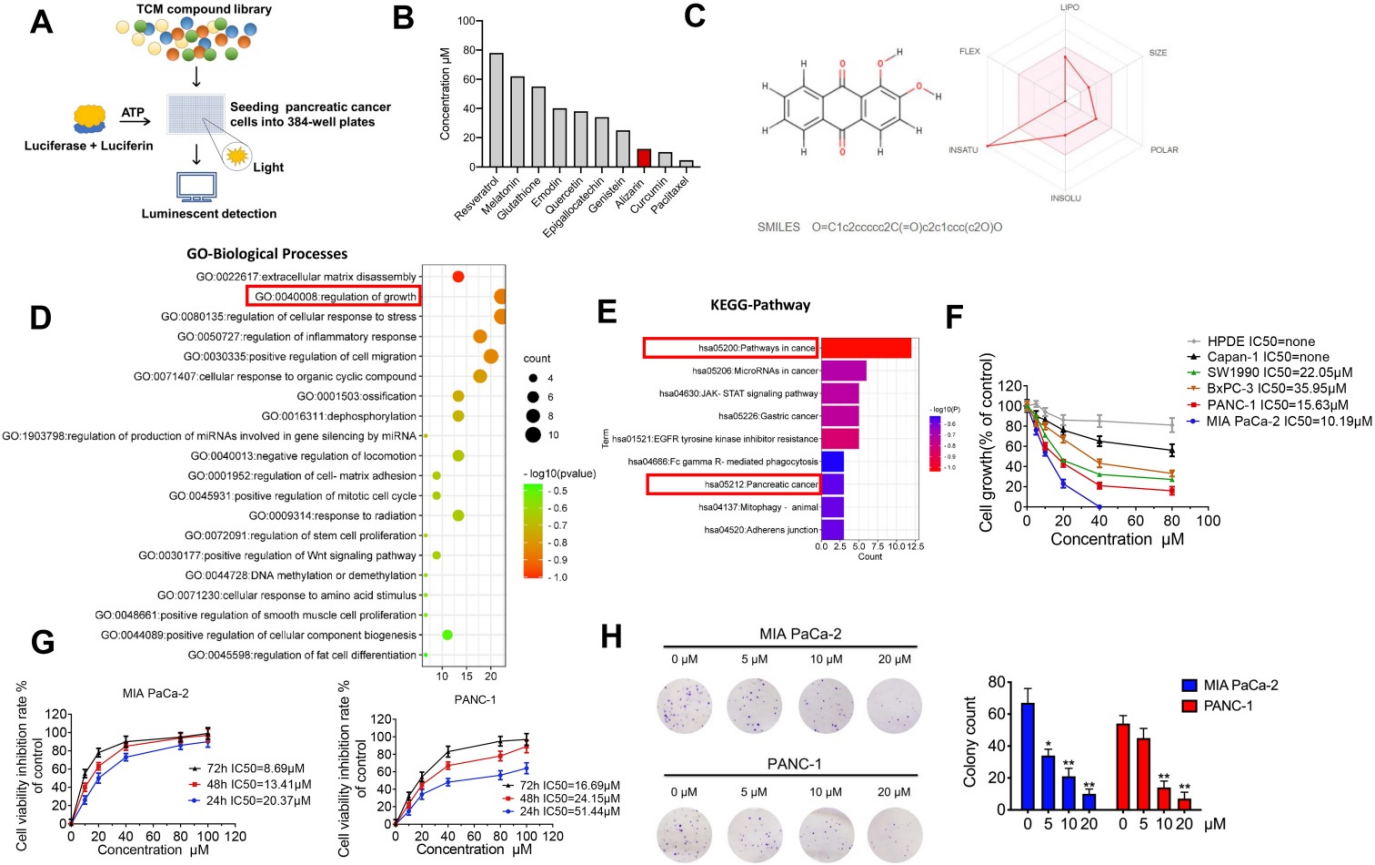
** The inhibitory effect of alizarin, a Chinese medicine, on the growth of pancreatic cancer. A-B.** 10 nature compounds with significant inhibitory effects on pancreatic cancer were selected from a chemical library containing 2951 candidates by cell viability assay. The IC50 of 10 natural compounds was shown and this experiment was repeated 3 times. **C.** Chemical formula of Chinese medicine monomer alizarin (left) and its ADME parameters (figure made in http://www.swissadme.ch/index.php) (right). **D.** GO enrichment analysis of alizarin. **E.** KEGG enrichment analysis of signaling pathway of alizarin. **F.** The inhibitory effect of different concentrations of alizarin (0μM, 5μM, 10μM, 20μM, 40μM and 80μM) on 5 types of pancreatic cancer cells (PANC-1, MIAPaCa-2, Capan-1, SW1990, BxPC3 cells) and normal human pancreatic ductal epithelial cell (HPDE). **G.** CCK8 was used to detect the time- and dose-dependent inhibitory effect of alizarin on PANC-1 and MIA PaCa-2. **H.** The clone formation experiment was used to detect the effect of different concentrations of alizarin (0μM, 5μM, 10μM, 20μM) on the clonogenic ability of MIA PaCa-2 and PANC-1 cells. *P <0.05, **P <0.01.

Previously, we established a novel luminescence-based high-throughput screening system 9 that can identify natural products with antitumor activities (Fig. 1A). After screening 2951 candidate products (Supplementary table 1), ten active ingredients caught our attention: paclitaxel, curcumin, resveratrol, glutathione, melatonin, genistein, emodin, quercetin, epigallocatechin and alizarin, which possess potential anti-pancreatic cancer activity (Fig. 1B). Among them, paclitaxel, curcumin and alizarin showed better inhibitory effects than the others. There have already been many studies about paclitaxel and curcumin in the pancreatic cancer field, and these agents have shown potent tumor-inhibitory capability 13, 14, consistent with our work. However, to date, no study has uncovered a suppressive effect of alizarin on pancreatic cancer cells. Therefore, we focused on alizarin, an anthraquinone compound that is the active ingredient of the Chinese herb Radix rubiae (Qian cao) and we predicted the ADME parameters of alizarin to support drug discovery (Fig. 1B&C). In addition, based on network pharmacology, GO function and KEGG pathway enrichment analysis also showed that the targets and pathways of alizarin are closely related to the progression of pancreatic cancer (Fig. 1D&E). To further investigate the antitumor properties of alizarin, we used CCK8 assays to evaluate its inhibitory effects on different pancreatic cancer cell lines (Capan1, SW1990, BxPC3, PANC-1 and MIA PaCa-2) and normal human pancreatic ductal epithelial (HPDE) cells after treatment at various concentrations (0, 20, 40, 60, and 80 μM) for 48 h. We found that PANC-1 and MIA PaCa-2 cells were more sensitive to alizarin, and its antitumor activity was dose- and time-dependent. Alizarin showed minimal cytotoxicity against HDPE cells (Fig. 1F&G). The results of the clone formation assay further confirmed that alizarin's suppressive effect on pancreatic cancer cells occurred in a dose-dependent fashion, and the effective concentration was determined to be 10 μM (Fig. 1H).

### Alizarin arrested the cell cycle and induced apoptosis of pancreatic cancer cells

**Figure 2:**
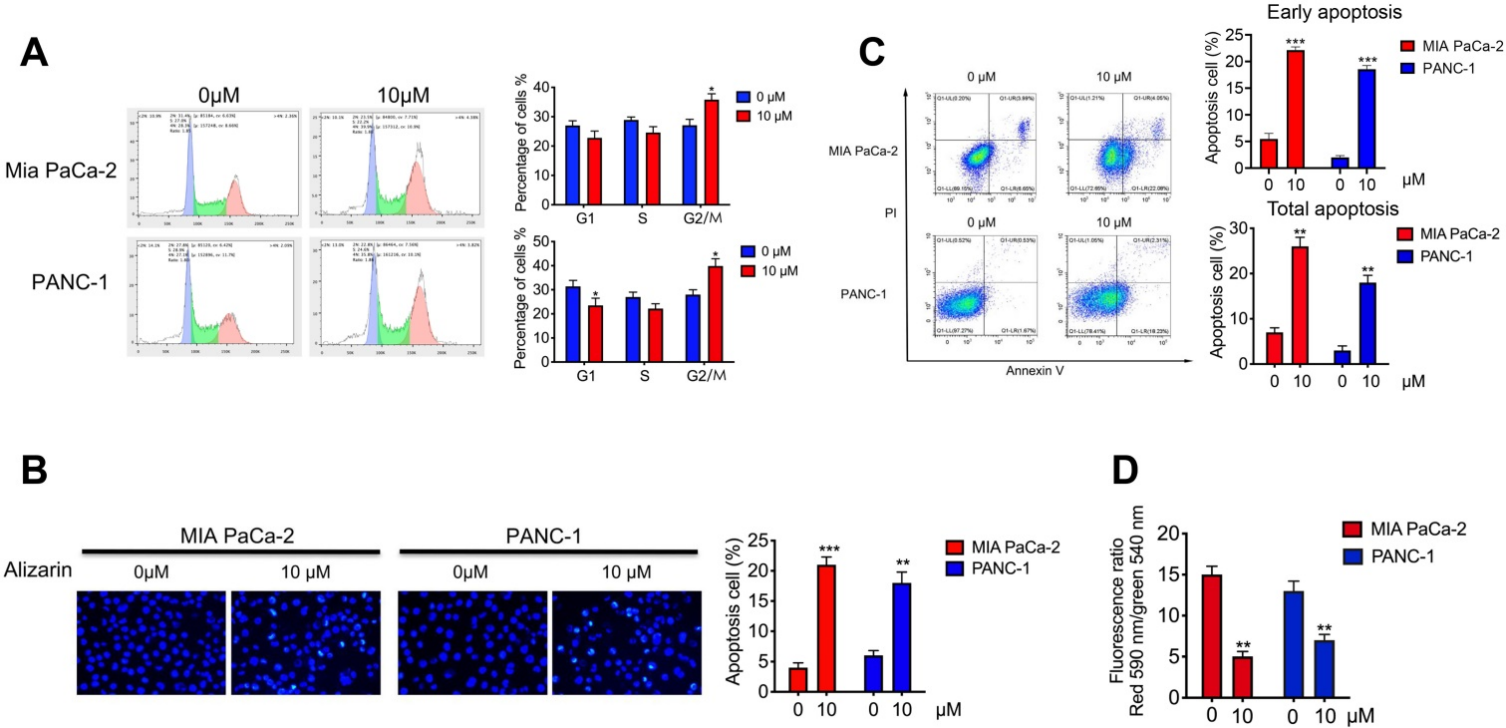
** The effect of alizarin on the cycle progression and apoptosis of pancreatic cancer cells. A.** Flow cytometry was used to detect the cell cycle progression of PANC-1 and MIA PaCa-2 after incubated with alizarin (0, 10 μM) for 48 h. **B-C.** Annexin V/PI staining and Hoechst 33342 staining were used to detect cell apoptosis efficacy of PANC-1 and Miapaca-2 cells after incubated with alizarin (0,10μM) for 48 h. **D.** Changes of mitochondrial membrane potential of PANC-1 and MIA PaCa-2 cells treated with alizarin (0, 10μM) for 48 h. *P <0.05, **P <0.01.

To further study the effect of alizarin on the growth inhibition of pancreatic cancer cells (PANC-1 and MIA PaCa-2), we assessed the cell cycle by flow cytometry after alizarin treatment (0 or 10 μM). We found that pancreatic cancer cells were arrested in the G2/M phase after the application of alizarin; subsequently, the number of cells in the G1 phase and S phase was decreased (Fig. 2A), indicating that alizarin can inhibit the progression of the pancreatic cancer cell cycle. Next, we used Hoechst 33342 staining to detect whether pancreatic cancer cells had undergone apoptosis. Based on the results, we found that the proportion of condensed and fragmented nuclei (indicating apoptotic cells), was significantly increased after the usage of alizarin (Fig. 2B). In addition, annexin V/PI double staining was carried out to further verify the induction of pancreatic cancer cellular apoptosis by alizarin. Flow cytometry analysis showed that the proportion of apoptotic cells (Annexin V+/PI-; representing early apoptotic cells) was obviously increased (Fig. 2C). Furthermore, the remarkable decrease in cell membrane potential after alizarin treatment confirmed the apoptosis of these pancreatic cancer cells (Fig. 2D).

### Alizarin induced cellular apoptosis by inhibiting NF-κB activation and TNF-α-triggered nuclear translocation

**Figure 3:**
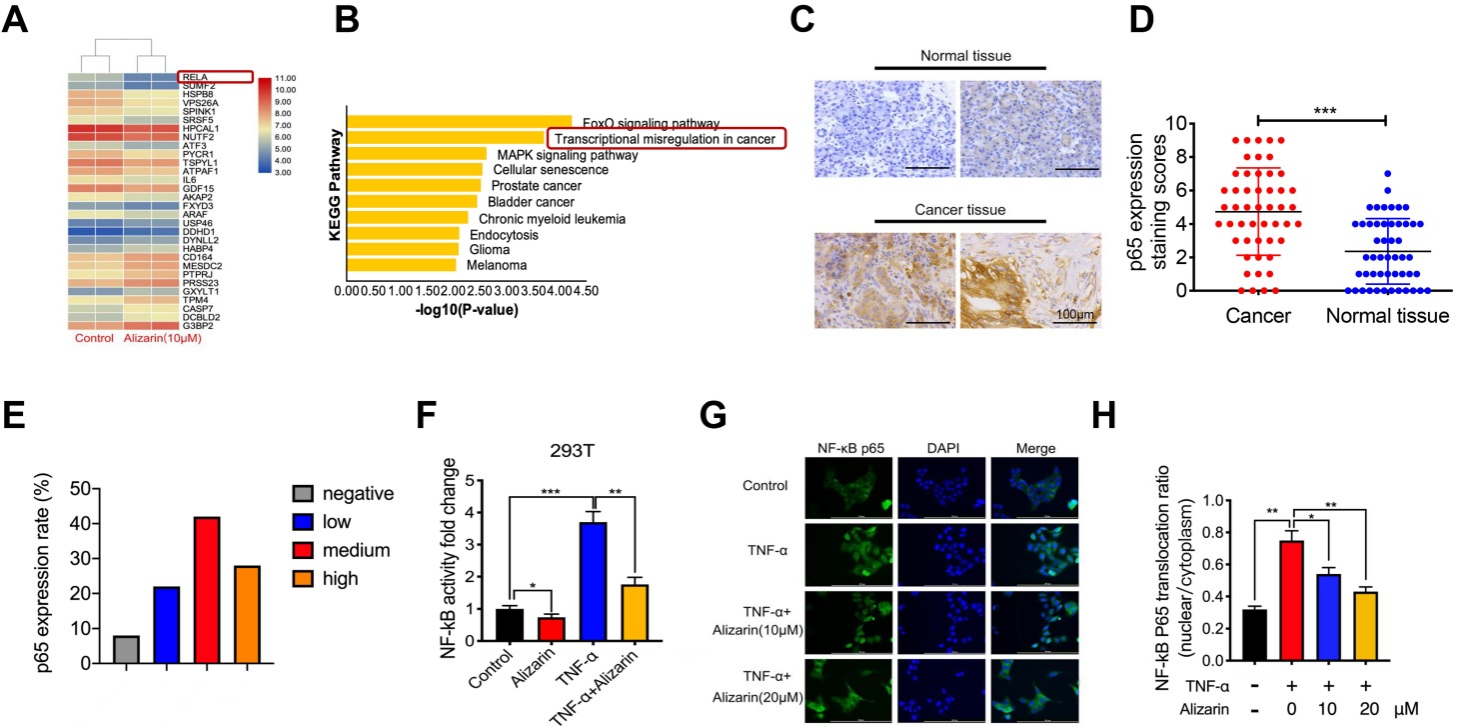
** Alizarin inhibits NF-κB activation and TNF-α-induced nuclear translocation of NF-κB in pancreatic cancer cells. A.** Gene chip was used to screen the differentially expressed genes of PANC-1 after incubated with alizarin (0,10 μM) for 48 h. **B.** The KEGG pathway was applied to analyze the significantly changed signaling pathways in PANC-1 treated with alizarin. **C-E.** Immunohistochemistry of p65 in tumor tissues of 50 patients. The results were observed under a microscope (× 400). **F.** NF-κB reporter assay for 293T cells treated with alizarin. The Renilla luciferase control vector was co-expressed with the NF-κB reporter luciferase construct in 293T cells. 293T cells were incubated with alizarin (10μM) for 24 hours followed by treatment with or without TNF-α (10 ng/mL), and luciferase activity was measured and normalized by use of a dual luciferase reporter assay. **G.** Immunofluorescence staining of NF-κB p65 in 293T cells after treatment with the indicated alizarin (10, 20 μM) for 48 h, followed by stimulation with or without TNF-α (10 ng/mL). Blue indicated nuclei stained with DAPI and green signaling represented p65 staining. Scale bar, 200 μm. **H.** Thermo Scientifific ArrayScan VTI HCS software was applied to analyze the cytoplasmic-nuclear translocatio of NF-κB p65. *P <0.05, **P <0.01, ***P < 0.001.

To further explore the molecular mechanism of alizarin's inhibitory effect on pancreatic cancer cells, an Affymetrix Gene Chip Human Genome Array was applied to detect the mRNA expression profiles in PANC-1 cells with or without alizarin treatment. The heat map and bioinformatics gene expression array analyses showed that there were significant alterations in the NF-κB signaling pathway at the mRNA level in PANC-1 cells treated with alizarin (Fig. 3A&B). NF-κB and p65 signaling plays a crucial role in the occurrence and development of many types of cancers, and various NF-κB target genes are involved in cell cycle progression and apoptosis 15, 16. In addition, our clinical data also showed that p65 expression in tumor tissues from 50 pancreatic patients was significantly higher than that in paracarcinomatous tissues, as detected by IHC (Fig. 3C-E). In unstimulated cells such as 293T cells, NF-κB binds to IκBα (an inhibitory protein of NF-κB) and remains in the cytoplasm. Several stimuli, including TNF-α, can activate NF-κB signaling by modulating downstream molecules. A hallmark of TNF-α stimulation is nuclear translocation of NF-κB and transcription of target genes 17. Thus, to clarify the effect of alizarin on the NF-κB signaling cascade, 293T cells were stimulated by tumor necrosis factor α (TNF-α), and the activity of NF-κB in 293T cells was detected by dual luciferase reporter assay. NF-κB activity was significantly upregulated after TNF-α stimulation compared with the control, while alizarin obviously antagonized this increase even after stimulation with TNF-α (Figure 3F). To further clarify whether the decrease in NF-κB activity was due to reduced nuclear translocation of NF-κB after alizarin treatment, laser-scanning confocal microscopy was used to observe the nuclear translocation of NF-κB after the administration of TNF-α, TNF-α+alizarin (10 μM), and TNF-α+alizarin (20 μM). The results showed that TNF-α stimulation significantly promoted the nuclear translocation of NF-κB; in contrast, alizarin inhibited this nuclear translocation induced by TNF-α stimulation in a dose-dependent fashion (Figure 3G&H). Taken together, these finding showed that alizarin inhibited the activity of NF-κB and reduced its nuclear translocation.

### Alizarin inactivated TNF-α-mediated TAK1-NF-κB signaling cascades in pancreatic cancer cells

**Figure 4:**
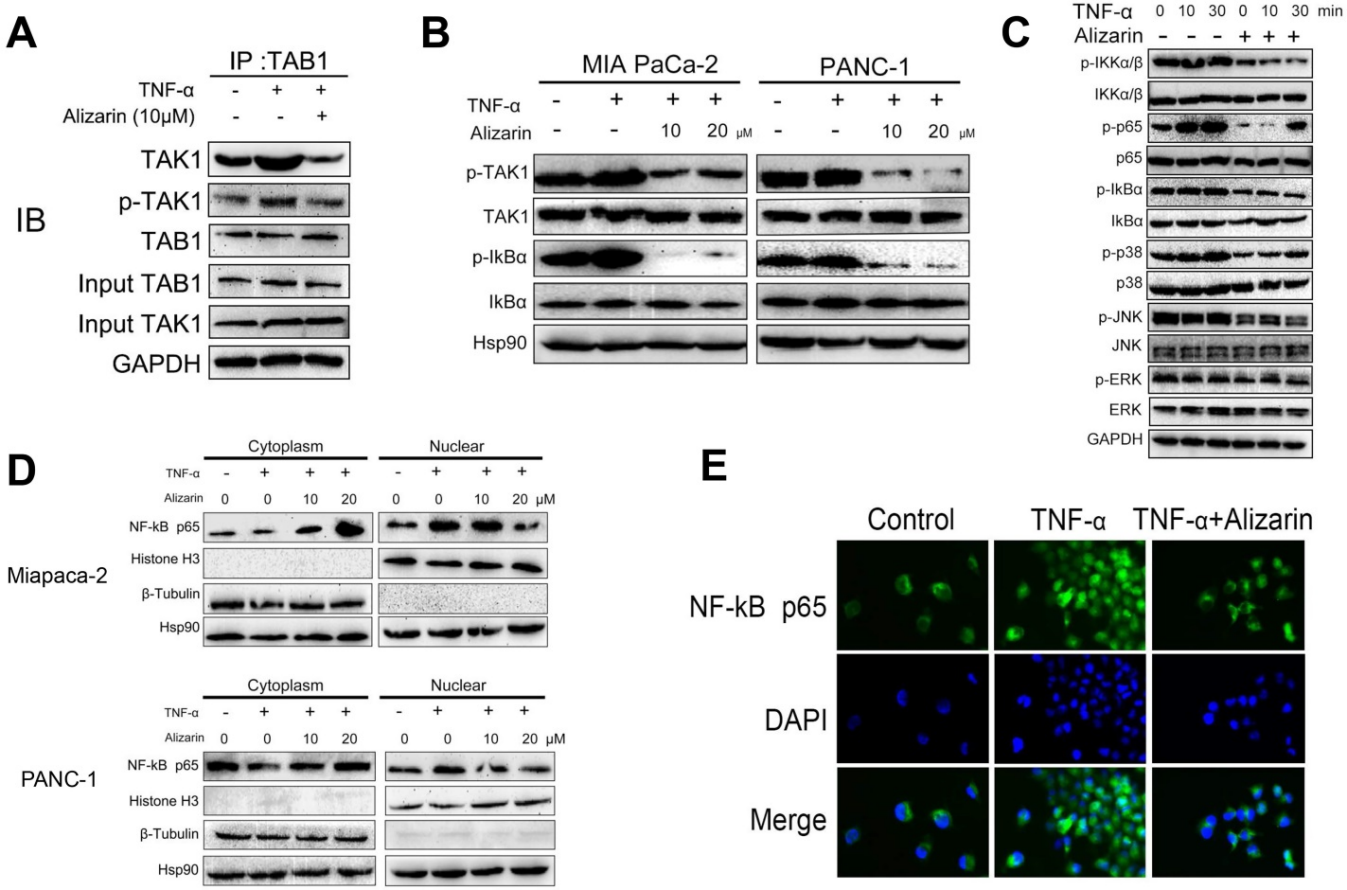
** The regulatory effect of alizarin on the TNF-α-TAK1-NF-κB signal cascade in pancreatic cancer cells. A.** MIA PaCa-2 cells were exposed to alizarin (10 μM) for 48 hours, then stimulated with TNF-α (10 ng/mL) for 30 minutes. Immunoprecipitation assays were conducted to evaluate the effect of alizarin on the binding affinity of total and phosphorylated TAK1 and TAB1 stimulated with TNF-α. **B.** Western blotting was used to detected the dose-dependent (10, 20 μM) effect of alizarin on activation of TAK1 and IKBα in MIA PaCa-2 and PANC-1 cells. **C.** Western blotting was applied to detect the alteration in the downstream (phosphorylated and total IKKα/β, IkBα, p38, JNK, ERK, and p65) of NF-κB signaling cascade of TAK1 after alizarin treatment (10 μM) and TNF-α (10 ng/mL) for different time intervals. **D.** The MIA PaCa-2 and PANC-1 cells were pretreated with alizarin (10, 20 μM) at the specified concentration for 48h and stimulated with TNF-α (10 ng/mL) and NF-κB p65 expression in the cytoplasm and nucleus were detected. **E.** Immunofluorescence staining was used to detect NF-κB p65 after MIA PaCa-2 cells were treated with alizarin and TNF-α.

It is well known that TGF-β-activated kinase 1 (TAK1) binds to the adaptor protein TAB1 to mediate the activation of downstream NF-κB signaling cascades 18. In addition, studies have shown that the TAK1-NF-κB signaling pathway plays a vital role in the progression of lymphoma and mediates tumor cell apoptosis through this signaling 19, 20. Based on our data in Figure 3, we found that alizarin could reduce the activity of NF-κB through TNF-α. Thus, we hypothesized that alizarin can also inhibit pancreatic cancer cells by affecting TNF-α-mediated TAK1-NF-κB signaling cascades. To test this hypothesis, a coimmunoprecipitation assay was carried out to detect the affinity between TAK1 and TAB1 induced by TNF-α. The binding ability of TAK1 to TAB1 was increased after TNF-α stimulation, but alizarin treatment reversed the effects on total TAK1 and the binding ability of phosphorylated TAK1 to TAB1 that were stimulated by TNF-α (Figure 4A). Next, pancreatic cancer cells (PANC-1 and MIA PaCa-2) were treated with different concentrations of alizarin (10 and 20 μM), and alizarin inhibited the phosphorylation of TAK1 and IKBα in a dose-dependent manner (Figure 4B). These results suggested that alizarin not only attenuated the TNF-α-induced interaction between TAB1 and TAK1 but also inhibited the recruitment of the TAK1/TAB1 complex, thereby mediating TAK1 inactivation and blocking downstream signaling. Then, Western blotting was used to detect alterations in the downstream (NF-κB) signaling cascade of TAK1 after alizarin treatment (10 μM) and TNF-α stimulation. Our results showed that the phosphorylation levels of IKKα/β, NF-κB, p65, IkBα, p38, JNK and ERK increased in a time-dependent manner after TNF-α (10 min and 30 min) stimulation in pancreatic cancer cells (MIA PaCa-2) (Fig. 4C). However, the enhanced phosphorylation of these proteins was reversed after the application of alizarin, indicating that alizarin can inhibit the TAK1-NF-κB signaling pathway. In addition, nuclear and cytoplasmic extraction assays (Fig. 4D) and immunofluorescence assays (Fig. 4E) were conducted to determine whether alizarin could inhibit the nuclear translocation of NF-κB. The results show that after TNF-α stimulated pancreatic cancer cells, the cytoplasmic p65 was decreased and the nuclear p65 expression was increased significantly. On this basis, alizarin prevented the nuclear translocation of NF-κB, resulting in an increase in cytoplasmic p65 and a decrease in nuclear p65, which is consistent with our results 3. According to the above data, alizarin not only inactivated the TNF-α-mediated TAK1-NF-κB signaling cascade but also significantly antagonized the nuclear translocation of NF-κB in pancreatic cancer cells.

### Alizarin induced cell cycle arrest and cellular apoptosis via NF-κB target genes

**Figure 5:**
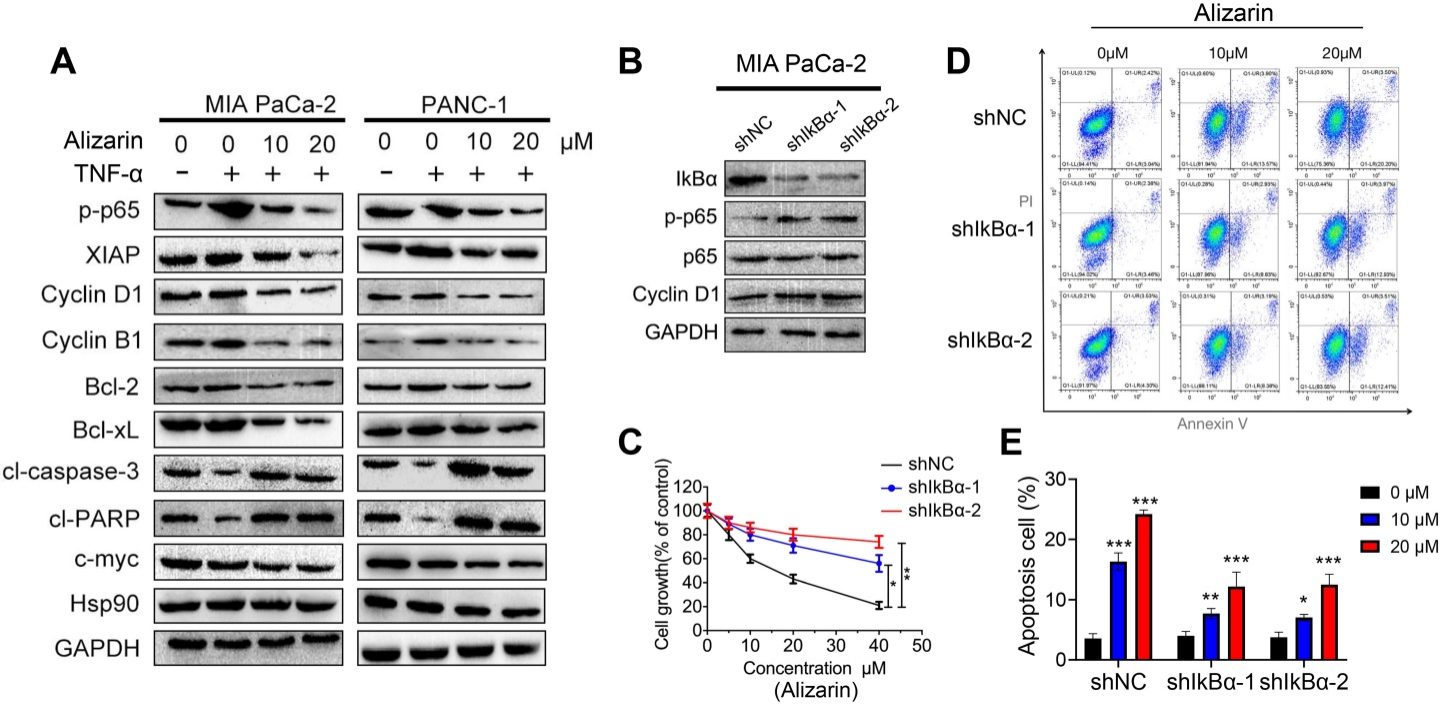
** Alizarin induced cell-cycle arrest and cellular apoptosis via NF-κB transcriptional target genes. A.** Western blotting was applied to detect protein expression levels in MIA PaCa-2 and PANC-1 cells treated with alizarin (10, 20 μM) and TNF-α (10 ng/mL). **B.** Western blotting was used to detect lentiviral infection-mediated IkBα knockdown and the protein expression of p65, phosphorylated p65 and NF-κB target genes Cyclin D1. **C.** MIA PaCa-2 cells in the IkBα knockdown group and the control group were treated with different concentrations of alizarin (0 μM, 5 μM, 10 μM, 20 μM and 40 μM) for 48 hours, and the CCK-8 experiment was applied to detect cell viability. **D-E.** MIA PaCa-2 cells were treated with alizarin (1, 10, 20μM) for 48 hours after IkBα knockdown, and the apoptosis of each group was detected by flow cytometry. *P <0.05, **P <0.01.

NF-κB target genes are the intersection point of multiple signal transduction pathways and important transcription factors in a variety of tumor cells that can regulate downstream inflammatory factors and other related proteins that are widely involved in the cell cycle and apoptosis, thus monitoring tumor development 15. In our study, we found that alizarin could lead to pancreatic cancer cell cycle arrest in the G2/M phase and induce apoptosis (Fig. 2). In addition, we also found that alizarin inhibited the expression of NF-κB (Fig. 3). Therefore, we hypothesized that alizarin-induced inhibition of NF-κB can decrease the expression of antiapoptotic proteins and cyclins and increase the expression of proapoptotic proteins. To test our hypothesis, the proteins mentioned above were detected by Western blotting. In addition to the antiapoptotic protein p65, another classic antiapoptotic protein, XIAP, Bcl-2 and Bcl-xL, which are downstream of NF-κB, were also downregulated by alizarin treatment (Fig. 5A). In contrast, the proapoptotic proteins cleaved caspase-3 and cleaved PARP were significantly upregulated due to the administration of alizarin (Fig. 5A). Moreover, cyclins (cyclin B1 and cyclin D1) were remarkably reduced after alizarin treatment, which was consistent with the flow cytometry results (shown in Fig. 2), showing the inhibitory effect of alizarin on cell growth. The expression of c-myc, which is closely associated with cell apoptosis, was also consequently reduced after the administration of alizarin (Fig. 5A). All of these proteins changed more with TNF-α stimulation than without, and all these changes were induced by alizarin + TNF-α in a concentration-dependent manner (Fig. 5A). In general, alizarin promoted cell cycle arrest and triggered cellular apoptosis by inhibiting NF-κB signaling, consistent with the data shown in Figure 2. To determine whether NF-κB plays a dominant role in alizarin-mediated anti-pancreatic cancer activity, and given that there was a reduction in IkBα, an NF-κB inhibitor was required for canonical NF-κB activation 21. Therefore, lentivirus-mediated short hairpins RNAs (shRNA) were employed to efficiently knockdown IkBα in MIA PaCa-2 cells. Then, we hypothesized that constitutive activation of NF-κB through RNAi-mediated IkBα downregulation would reduce the inhibitory effect of alizarin on cell growth. The results showed that the decrease in IkBα contributed to an increase in NF-κB p65 expression and phosphorylation (Fig. 5B) as well as an increase in the expression of the NF-κB target gene cyclin D1 (Fig. 5B), showing activation of the NF-κB signal cascade. MIA PaCa-2 cells with activation of NF-κB induced by either of 2 independent shRNAs exhibited attenuated sensitivity to alizarin and had less apoptosis after alizarin treatment than control (sh NC) cells, as detected by annexin V+/PI+ staining (Fig. 5C-E). These data indicated that alizarin exerted an inhibitory effect on cell growth and a promoting effect on apoptosis via inactivation of NF-κB signaling.

### Alizarin inhibited tumorigenesis in an orthotopic xenograft tumor model of pancreatic cancer

**Figure 6:**
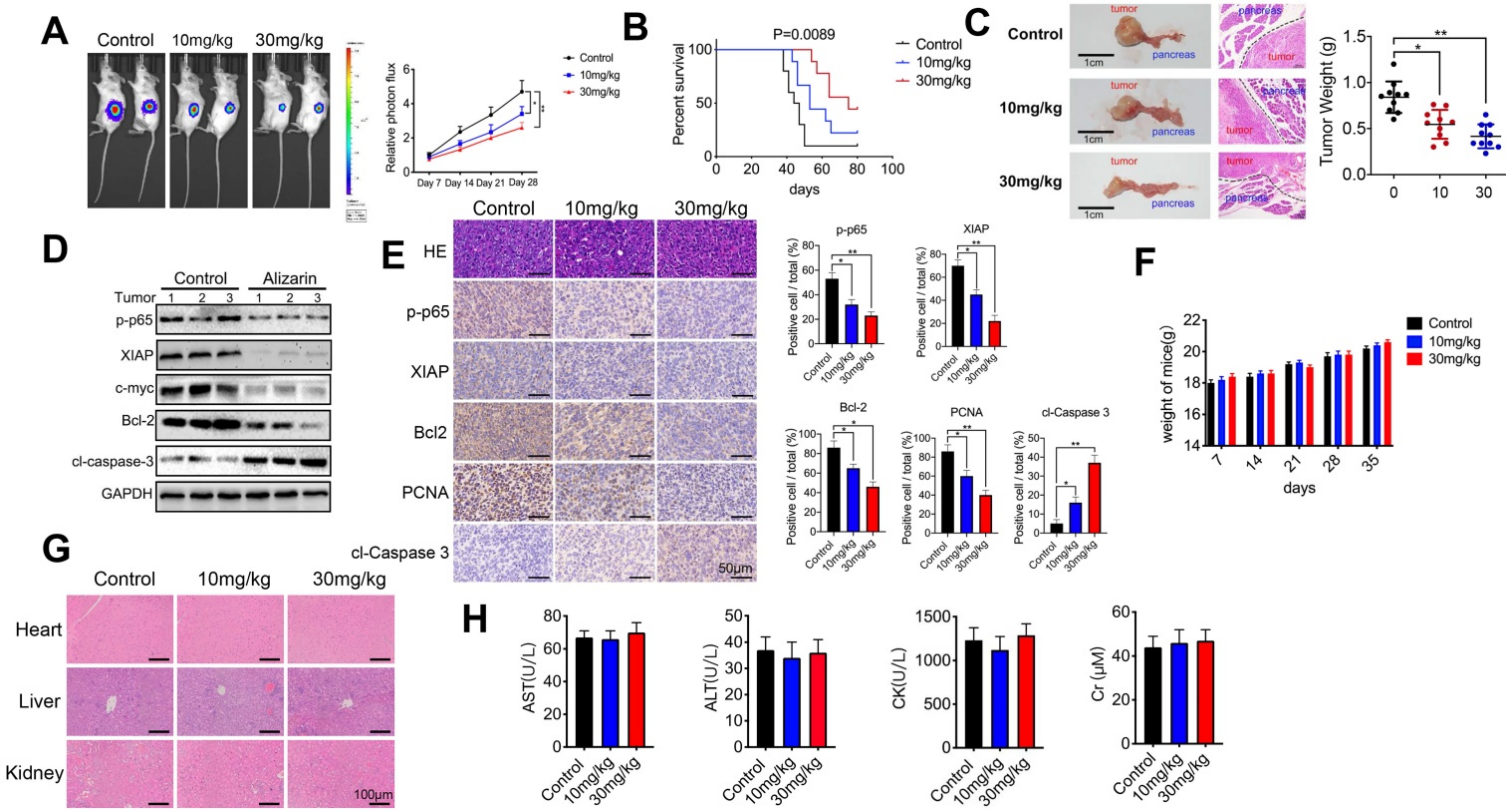
** Alizarin inhibited tumorigenesis in an orthotopic xenograft tumor model with pancreatic cancer. A.** Different concentrations of alizarin (10, 30mg/kg) were injected intraperitoneally one week after the mouse pancreatic cancer was inoculated *in situ*, and the bioluminescence images of mice were performed on day 7, 14, 21, and 28 after tumor cell injection to observe the tumor growth after treatment in different treatment groups. **B.** The kaplan-Meier survival analysis was used to monitor the survival time of mice treated with different concentrations of alizarin. **C.** Tumor size and weight in mice after treatment with different concentrations of alizarin were detected. **D.** Western blotting was constructed to detect the expression levels of p-p65, XIAP, c-myc, Bcl-2 and cl-caspase-3 in pancreatic tumor tissues treated with alizarin (30mg/kg). **E.** Immunohistochemistry was used to detect the expression of p-p65, XIAP, Bcl-2, PCNA and cl-caspase-3 in pancreatic tumor tissues after alizarin treatment. The results were observed under a microscope (× 400). **F.** Changes in body weight of mice after alizarin treatment were recorded. **G.** H&E staining of mouse liver, kidney and heart tissues treated with alizarin was constructed. The results were observed under a microscope (× 100). **H.** The heart, liver and kidney function indexes of mice treated with alizarin were detected by ELISA. *P < 0.05, **P < 0.01.

As mentioned above, alizarin-induced apoptosis of pancreatic cancer cells through the NF-κB signaling pathway was preliminarily confirmed *in vitro*. Next, we further examined the inhibitory effect mediated by alizarin in tumor-bearing mice. An orthotopic xenograft tumor mouse model with pancreatic cancer was successfully established with MIA PaCa-2-Luc cells (1×10^6^) through pancreas injection (Fig. 6A). Alizarin (10 or 30 mg/kg) was administered once daily for 28 days one week after inoculation. The tumor growth of mice was monitored by *in vivo* imaging once a week for 4 weeks. According to the data, although 10 mg/kg alizarin showed a good inhibitory effect compared with the control treatment, the antitumor capability of 30 mg/kg alizarin was more remarkable (Fig. 6A). The survival of tumor-bearing mice after alizarin treatment (10 or 30 mg/kg) was consistent (Fig. 6B). Pancreatic tissues were collected from the model mice, and the tumor weight results were consistent with the optical image data (Fig. 6C). In addition, Western blotting was carried out to detect the protein expression levels of p-p65, c-myc, XIAP, Bcl-2 and cleaved caspase-3 in tumor tissues. After the administration of alizarin (30 mg/kg), the antiapoptotic-related proteins p-p65, XIAP, Bcl-2 and c-myc were downregulated, while the proapoptotic protein cleaved caspase 3 was upregulated (Fig. 6D). IHC was employed to further confirm the Western blotting results (Fig. 6E), and the IHC results were in line with the *in vitro* results shown in Figure 5. Furthermore, we found that the administration of alizarin (10 or 30 mg/kg) not only had no obvious effect on the body weight of the tumor-bearing mice but also had no overt impact on the tissue structure and biochemical function of the liver, kidney and heart (Fig. 6F-H). In addition, as seen in Figure 1D, alizarin had almost no inhibitory effect on HDPE cells. All of these results indicated that alizarin possessed minimal cytotoxicity. Taken together, the abovementioned data showed that alizarin inhibited pancreatic tumor growth via induction of cellular apoptosis through the NF-κB signaling pathway, with almost no obvious side effects.

### Alizarin combined with Gem exerted synergistic effect to inhibit pancreatic cancer by inactivating NF-κB signaling

**Figure 7:**
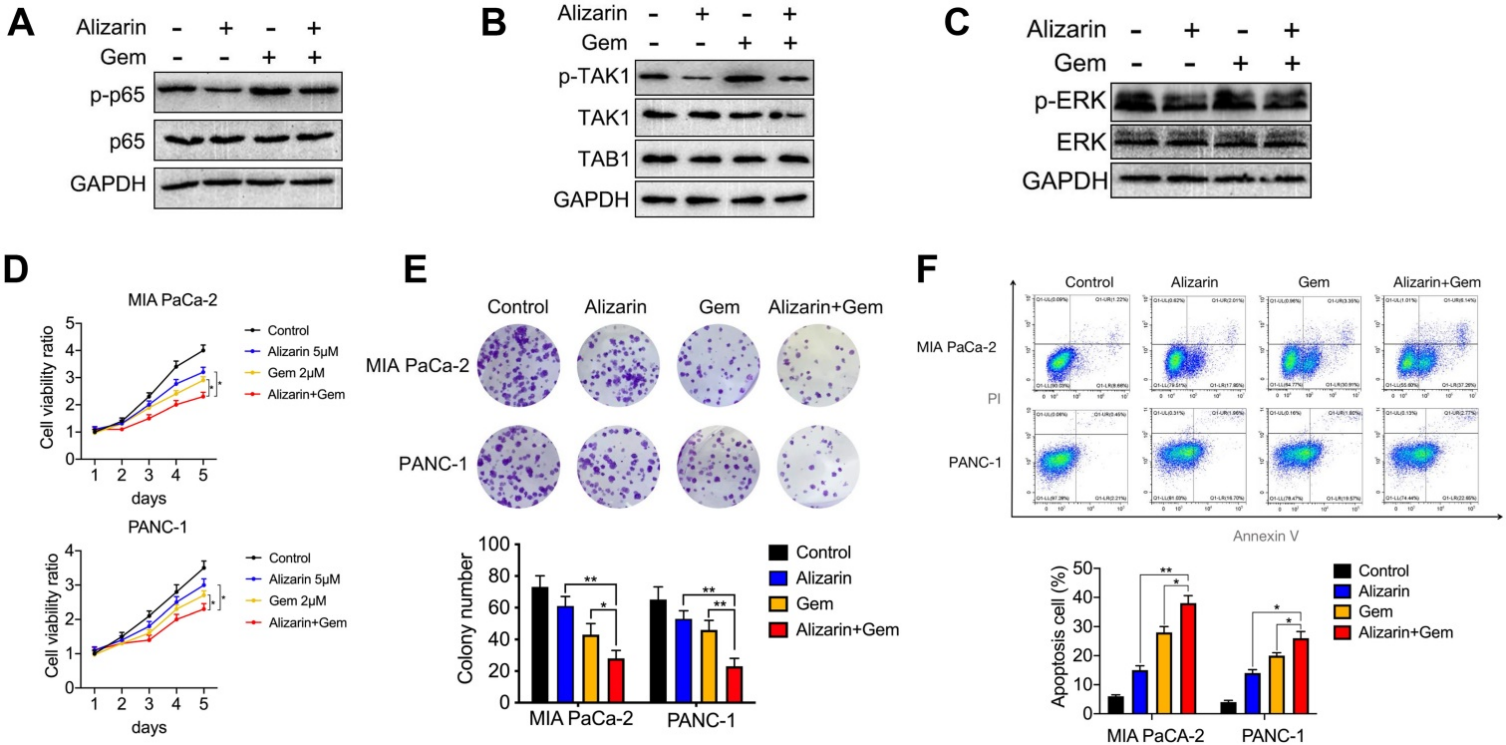
** Gem combined with alizarin inhibits the growth of pancreatic cancer cells *in vitro*. A-C.** Alizarin (5 μM) and Gem (2 μM) were used to treat pancreatic cancer MIA PaCa-2 cells for 48h. Western blotting was used to detect the expression of p-p65, p65, p-TAK1, TAK1, TAB1, p-ERK, and ERK proteins. **D.** The cell proliferation in the alizarin group, Gem group and the combined treatment was detected by CCK-8 experiment. **E.** MIA PaCa-2 and PANC-1 cells were planted into 6-well plates and treated with alizarin (5 μM) and Gem (2 μM) for 48h, and then the cell clone formation was detected after 2 weeks. **F.** MIA PaCa-2 and PANC-1 cells were treated with alizarin (5 μM) and Gem (2 μM) for 48h, and the apoptosis of each group was detected by flow cytometry. *P < 0.05, **P < 0.01.

**Figure 8:**
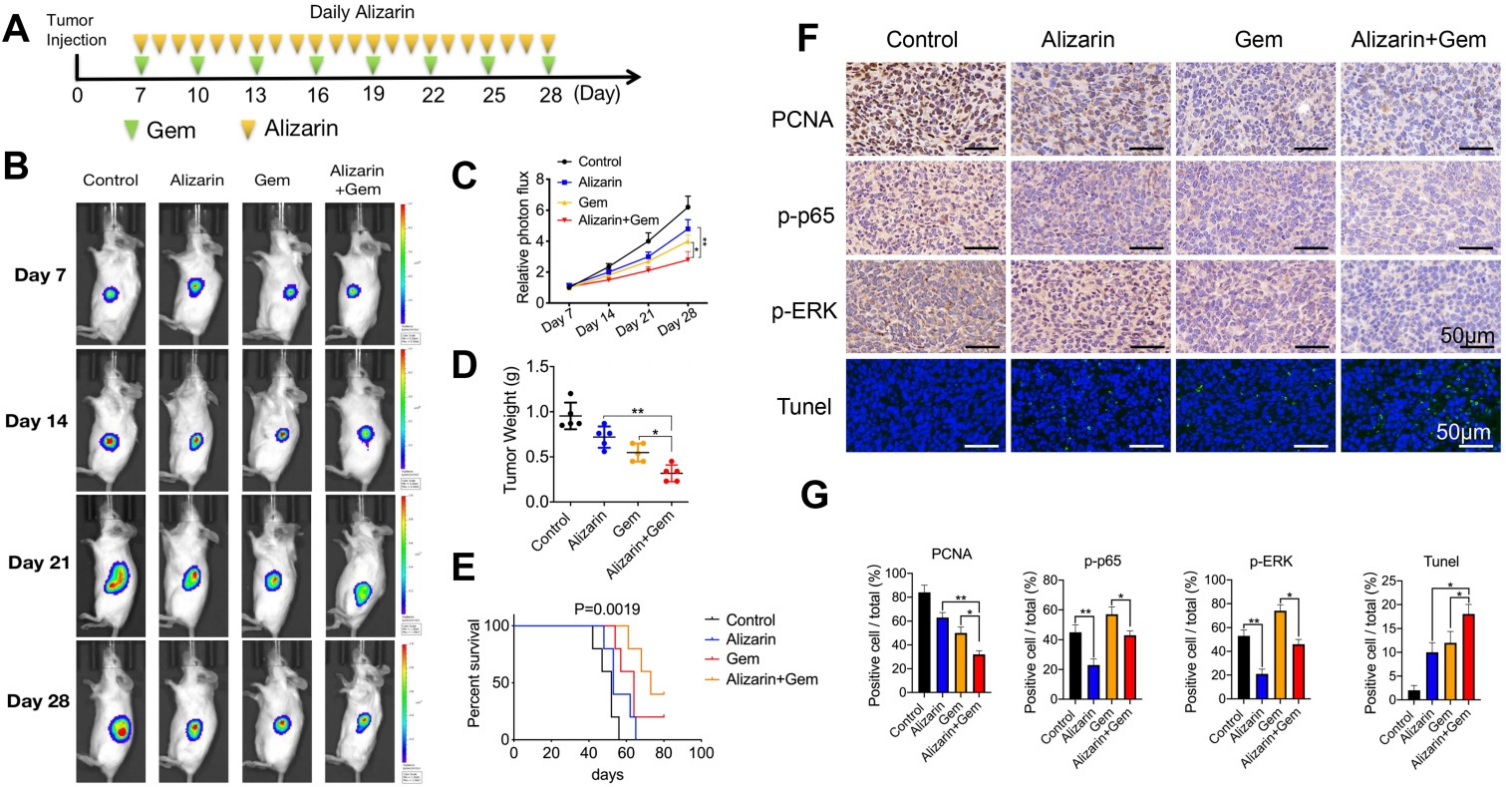
** Gem combined with alizarin inhibits the growth of pancreatic cancer *in vivo*. A.** 7 days after the mouse pancreatic cancer was inoculated *in situ*, alizarin (10 mg/kg) was administered intraperitoneally every day, Gem (5 mg/kg) was injected every 3 days, and Gem combined with alizarin was administered simultaneously. **B-C.** The bioluminescence images of mice were performed on day 7, 14, 21, and 28 after tumor cell injection to observe the tumor growth after treatment in different treatment groups. **D.** Tumor weight in mice after drug treatment in each group. **E.** Kaplan-Meier survival analysis was used to detect the survival time of mice treated with different drug treatment groups. **F-G.** Immunohistochemistry was constructed to detect the positive expression of p-p65, p-ERK and PCNA in pancreatic tumor tissue after treatment with different drug treatment groups and tumor tissue apoptosis was detected by TUNEL. The results were observed under a microscope (× 400). *P < 0.05, **P < 0.01.

Gem has been widely utilized as a first-line drug for advanced pancreatic cancer for many years 6, 22. Although it has been shown to improve survival, many patients suffer from treatment failure due to severe drug resistance, in which an increase in NF-κB activity is a major chemoresistance mechanism 10, 11. Our above data show that alizarin could effectively inhibit the activity of NF-κB. Therefore, we sought to determine whether the combination of alizarin and Gem has a synergistic effect. Western blotting was employed to detect the expression of p65, p-p65, p-TAK1, TAK1, TAB1, p-ERK, and ERK in pancreatic cancer cells (MIA PaCa-2) after exposure to alizarin, Gem and Gem + alizarin. We found that the expression of p-p65, p-TAK1, and p-ERK was significantly increased after Gem treatment, but they were decreased by administration of Gem + alizarin (Fig. 7A-C). In addition, CCK-8 assays and cell cloning assays were used to detect the effect of Gem, alizarin and Gem + alizarin on cell proliferation. The results also showed that the combination of Gem and alizarin possessed a superior cell growth inhibitory effect than Gem or alizarin alone (Fig. 7 D&E). In addition, this combination also induced more apoptosis of pancreatic cancer cells (Fig. 7 F), which further supports that the combination may induce a synergistic effect. The above data indicated that alizarin combined with Gem could effectively enhance increase the inhibition of tumor cell growth and promote apoptosis by blocking NF-κB signaling *in vitro*. Therefore, we next wanted to determine whether there was a similar suppressive effect in an orthotopic xenograft tumor mouse model of pancreatic cancer. Starting on the seventh day after the mouse pancreas was inoculated with MIA PaCa-2 cells *in situ*, Gem (5 mg/kg) was given intraperitoneally every three days, while alizarin (10 mg/kg) was intraperitoneally injected every day for 21 consecutive days (Fig. 8 A). An *in vivo* imaging system was used to monitor the tumor growth of the PBS (control), alizarin, Gem and alizarin + Gem treatment groups. According to the fluorescence signal data, we found that the combination treatment (alizarin + Gem) had a significantly stronger inhibitory effect on pancreatic tumor growth than either Gem or alizarin alone. The pancreatic tissue of tumor-bearing mice was removed and weighed, and the results were consistent with those of optical imaging (Fig. 8 B-D). Consistent results were also found in the analysis of survival of tumor-bearing mice after the different therapies (Fig. 8 E). Next, IHC was carried out to detect the protein expression of proliferating cell nuclear antigen (PCNA), p-p65, and p-ERK in pancreatic tissues after the above treatments (PBS, alizarin, Gem and alizarin + Gem). The results in Fig. 8F&G show that PCNA, a classic marker of cell proliferation, was significantly reduced in the combination (alizarin + Gem) group compared with the Gem or alizarin alone group. In addition, the expression levels of p-p65 and p-ERK were also significantly downregulated, which was consistent with the *in vitro* data in Figure 7A-C. Furthermore, the combination treatment (alizarin + Gem) significantly increased the number of apoptotic cells detected by the TUNEL assay (Fig. 8F&G). In general, the data in tumor-bearing mice provided further evidence that alizarin combined with Gem therapy inhibited tumor development by reducing NF-κB activity and was superior to alizarin or Gem alone, indicating a synergistic antitumor effect.

## Discussion

The combination of systemic chemotherapy including FOLFIRINOX (5-fluorouracil, leucovorin, irinotecan, and oxaliplatin) and Gem plus nerbu paclitaxel is still the main method for treating advanced pancreatic cancer patients 13, 23, 24. However, the response to chemotherapy remains poor because of the histologically dense proliferative matrix of pancreatic cancer and other factors, including drug resistance and patient tolerance 5, 25. Therefore, in-depth exploration of the molecular mechanism of pancreatic cancer and the development of new potent drugs are the key to improving its prognosis.

Recently, an increasing number of researchers have begun to pay attention to nature compounds which have good antitumor capability and almost no obvious side effects 26. A growing body of studies has shown that both Chinese herbs and their active components can directly inhibit tumors by inhibiting angiogenesis 27, blocking the tumor cell growth cycle 28-30, or inducing tumor cell apoptosis 31, 32 and indirectly inhibit tumors by regulating the tumor microenvironment; for example, by increasing the activity of NK cells 33, downregulating the immunosuppressive activity of myeloid-derived suppressor cells 28, 34, and improving the immune response of dendritic cells 35. Our lab has been committed to revealing the mechanism of the antitumor activity of nature compounds for years 28, 30, 31, 33.

**Figure 9:**
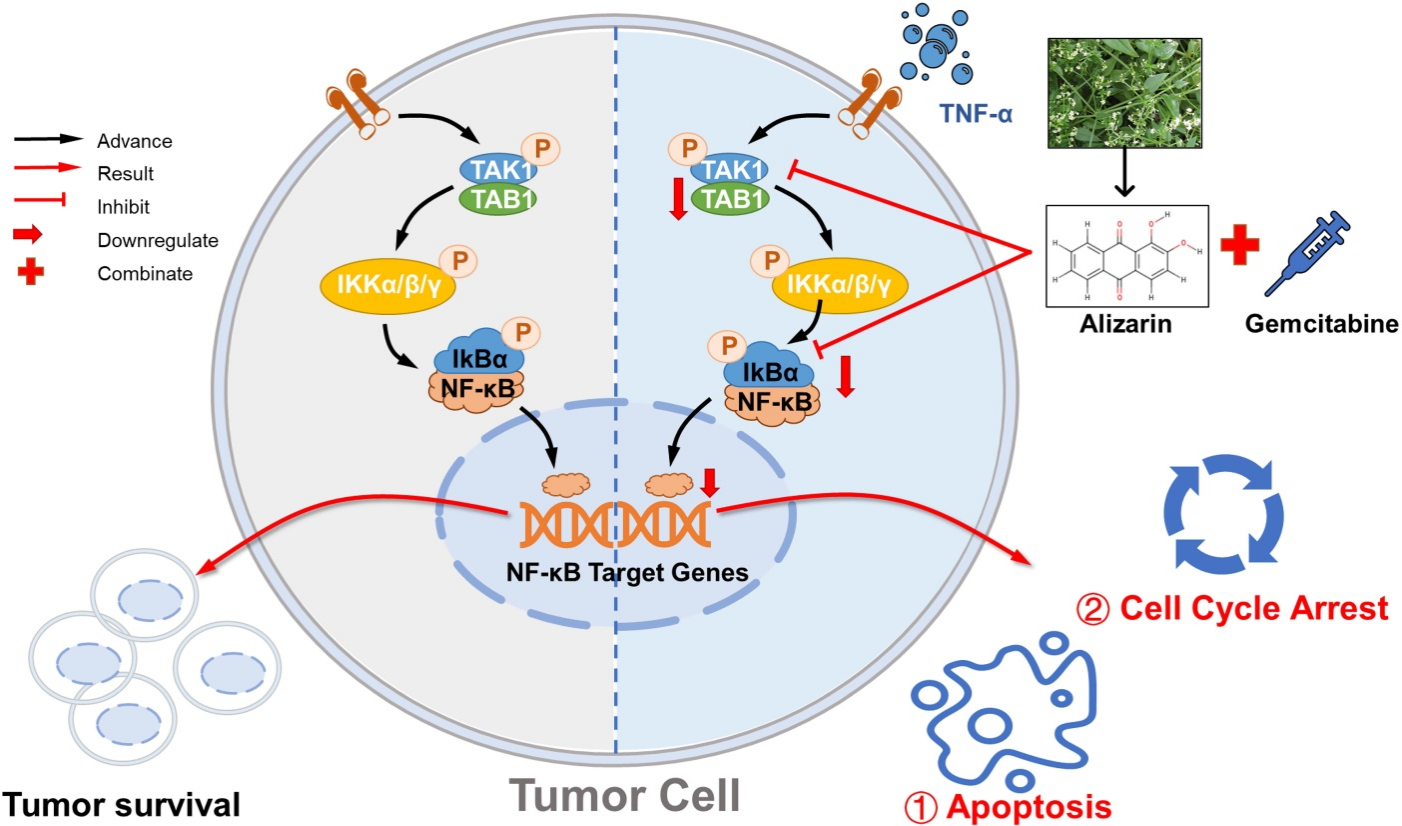
** Alizarin inhibits growth of pancreatic cancer cells by abrogating NF-κB activation.** Alizarin, the active ingredient of Chinese herb, can significantly inhibit the growth of pancreatic cancer cells. Alizarin arrests cell-cycle and induces cellular apoptosis through inactivation of the TNF-α-TAK1-NF-κB signal axis with minimal toxicity. In addition, due to the elimination of NF-κB activity, alizarin combined with Gem plays a synergistic anti-pancreatic cancer effect.

Previously, we established a luminescence-based high-throughput screening system to identify the effective components for natural products with activity against non-small-cell lung cancer (NSCLC). A total of 2880 kinds of compounds were screened; of these, thevebioside, the active ingredient of Thevetia peruviana (Pers) K. Schum (TPKS), an ornamental shrub belonging to the Gentianales order, was identified to inhibit SRC3-mediated signaling by ubiquitination to induce NSCLC cell apoptosis 31. Here, we used a similar method to screen a variety of natural candidates with actions against pancreatic tumor cells; these candidates are active components and are mainly distributed in the roots, stems, branches, leaves and fruits of Rubiaceae, Polygonaceae, Leguminaceae, Rhamnaceae, Liliaceae and other commonly used Chinese herbs, with laxative, antibacterial, anti-inflammatory, antitumor, antiviral, antioxidant and other pharmacological characteristics 31, 36. In our current study, we initially identified 10 nature compounds (paclitaxel, curcumin, resveratrol, glutathione, melatonin, genistein, emodin, quercetin, epigallocatechin and alizarin) with anti-pancreatic cancer activity via the abovementioned high-throughput screening system; of these, three (paclitaxel, curcumin and alizarin) were found to possess superior antitumor effects in the inhibition of cell growth and proliferation. Then, CCK-8 and clone formation assays were employed to further confirm the anticancer capability and compare these three active ingredients: pancreatic tumor cells exhibited higher sensitivity to paclitaxel (IC50: 4.6 μM) and curcumin (IC50: 10.4 μM) than to alizarin (IC50: 12.3 μM) (Fig. 1). However, there have already been many studies on paclitaxel and curcumin 13, 14, which have reported that these agents have potent inhibitory effects on pancreatic tumor cells, and our results are consistent with these existing studies. Nevertheless, there has not been any proof of the antitumor activity of alizarin in pancreatic cancer. Therefore, we focused on the capability of alizarin, not paclitaxel or curcumin, and to the best of our knowledge, this is the first report that alizarin can induce a potent inhibitory effect against pancreatic cancer (Fig. 9). We further revealed that alizarin triggered pancreatic tumor cell apoptosis by inhibiting nuclear translocation and weakening the activity of NF-κB after TNF-α stimulation (Fig. 3). Moreover, alizarin interrupted the upstream TAK1/TAB1 complex of NF-κB (Fig. 4), which inhibited apoptosis and suppressed the IKKα/β-IkBα-NF-κB signal cascade, leading to the reduction of the transcription of NF-κB target genes involved in cell growth, such as Bcl-2, Bcl-xl, XIAP, cyclin D, and c-myc (Fig. 5). These results were also further verified in an orthotopic xenograft tumor mouse model of pancreatic cancer (Fig. 6), indicating that alizarin exerts its antitumor activity by targeting the canonical NF-κB pathway. Of note, our clinical data also showed that p65 was overactivated in the tumor tissues compared with tumor-adjacent tissues of pancreatic cancer patients, which strongly supported our laboratory findings (Fig. 3C&D). Another interesting finding in our study was that the combination of alizarin and Gem showed potent synergistic antitumor effects in the orthotopic pancreatic cancer mouse model by inactivating NF-κB signaling (Fig. 7 & Fig. 8).

Gem, a cytotoxic nucleoside analog that is used alone or in combination, remains the current first-line chemotherapy regimen for pancreatic cancer. However, the overall response rate to Gem treatment in pancreatic cancer is less than 20%, and approximately 80% of patients die of tumor metastasis within 1 year 37. The resistance of pancreatic cancer to Gem is one of the most significant limitations of its application and leads to poor prognosis. The mechanism of Gem resistance is complex and may involve both congenital and acquired mechanisms, but the regulation of intracellular signaling pathways is the ultimate means leading to Gem resistance 6. Among the pathways, the NF-κB signaling pathway, which is associated with multiple pathophysiological processes, including the immune response, inflammation, proliferation, apoptosis, and metastasis, is overactivated, and this is one of the most common mechanisms of Gem resistance reported in many studies 11, 38. In addition, it has been documented in multiple articles that NF-κB p65 is highly expressed in pancreatic cancer patients 10, 39, which is also consistent with the results from our analysis of clinical samples (Fig. 3C). In our study, we collected tumor samples from 50 pancreatic cancer patients. According to the IHC results, the expression of p65 in the tumor tissues of pancreatic cancer patients was significantly higher than that in normal pancreatic tissues (Fig. 3D). These results may also partially explain why pancreatic cancer cells are sensitive to alizarin, since the earlier data (Fig. 2-Fig. 5) showed that alizarin can effectively reduce NF-κB activity. In addition, we also found that the application of Gem could overactivate NF-κB, in line with other studies 10, 11, but treatment with alizarin not only significantly inhibited the activity of NF-κB in pancreatic cancer cells but also obviously downregulated the increase in NF-κB caused by Gem when used in combination. In addition, the combination of alizarin and Gem suppressed cell growth and induced apoptosis of tumor cells more significantly than alizarin or Gem alone, which indicated that the combination had synergistic effects, in line with the *in vivo* data (Fig. 7-Fig. 8).

Due to its antitumor activity mediated by targeting of the NF-κB pathway, alizarin was found to have a dual therapeutic effect in our study. On the one hand, it induces a direct suppressive effect on the proliferation of pancreatic cancer cells and induces apoptosis by weaking NF-κB activity, and on the other hand, alizarin in combination with Gem can effectively achieve synergistic treatment effects (Fig. 9).

## Conclusion

In general, we found that alizarin, which is the active ingredient of the Chinese herb Radix rubiae, induces potent anti-pancreatic cancer effects with minimal toxicity by arresting the cell cycle and inducing cellular apoptosis through inactivation of the TNF-α-TAK1-NF-κB signaling axis. In addition, the combination of alizarin and the traditional chemotherapy drug Gem can significantly achieve a synergistic effect. In summary, alizarin is a potential effective complementary and alternative agent for pancreatic cancer.

## Supplementary Material

Supplementary table.

## Abbreviations

IARC: International Agency for Research on Cancer
DMSO: dimethyl sulfoxide
Luc: luciferace
H&E: hematoxylin-eosin
IHC: Immunohistochemical
ELISA: Enzyme linked immunosorbent assay
TNF-α: Tumor necrosis factor α
TAK1: TGF-β-activated kinase 1
shRNA: short hairpin RNAs
Gem: gemcitabine
NSCLC: non-small cell lung cancer
