# Supplementary material for: Alizarin, a nature compound, inhibits the growth of pancreatic cancer cells by abrogating NF-κB activation
Source: Int J Biol Sci. 2022 Mar 28;18(7):2759–74. doi: 10.7150/ijbs.70567 (PMC9066114; doi:10.7150/ijbs.70567)
Supplement: Supplementary file 1 — Supplementary table. [file ijbsv18p2759s1.pdf]

CAS#	NAME	M.W.	Conc.	Solvent
509-15-9	Gelsemine	322.401	10mM	DMSO
1358-76-5	Koumine	306.401	10mM	DMSO
82354-38-9	Humantenmine	326.39	10mM	DMSO
483-04-5	Ajmalicine	352.427	10mM	DMSO
84847-50-7	Vasicinolone	218.209	10mM	DMSO
1016983-51-9	Sulfocostunolide A	312.381	10mM	DMSO
1059671-65-6	Sulfocostunolide B	312.381	10mM	DMSO
1748-81-8	Carabrone	248.318	10mM	DMSO
68832-39-3	4-Epi-isoinuviscolide	248.318	10mM	DMSO
325686-49-5	Ascleposide E	388.453	10mM	DMSO
82375-29-9	Humantenine	354.443	10mM	DMSO
113973-31-2	(Z)-Akuammidine	352.427	10mM	DMSO
72715-02-7	Ilicol	238.366	10mM	DMSO
4290-13-5	Santamarine	248.318	10mM	DMSO
1139-30-6	Caryophyllene oxide	220.35	10mM	DMSO
553-21-9	Costunolide	232.318	10mM	DMSO
477-43-0	Dehydrocostus lactone	230.302	10mM	DMSO
6159-55-3	Vasicine	188.226	10mM	DMSO
87441-73-4	11(13)-Dehydroivaxillin	264.317	10mM	DMSO
57458-57-8	Stigmast-4-ene-3,6-dione	426.674	10mM	DMSO
22149-69-5	Stigmastane-3,6-dione	428.69	10mM	DMSO
295803-03-1	Gynuramide II	682.112	10mM	DMSO
2182-14-1	Vindoline	456.531	10mM	DMSO
115404-57-4	Niloticin	456.7	10mM	DMSO
355143-38-3	6'-O-p-Hydroxybenzoylcatalposide	602.54	10mM	DMSO
1016987-87-3	6-O-p-Hydroxybenzoylaucubin	466.435	10mM	DMSO
1615-94-7	Simiarenol	426.717	10mM	DMSO
3122-88-1	Eucalyptin	326.343	10mM	DMSO
177602-14-1	Glycerol 1-(26-hydroxyhexacosanoate)	486.768	10mM	DMSO
142698-60-0	Macrocarpal B	472.614	10mM	DMSO
142628-53-3	Macrocarpal C	454.598	10mM	DMSO
172617-99-1	Eucalyptone	486.597	10mM	DMSO
145382-68-9	Sideroxylonal A	500.495	10mM	DMSO
96087-10-4	Massoniresinol	392.4	10mM	DMSO
118-34-3	Syringin	372.367	10mM	DMSO
92-61-5	Scopoletin	192.168	10mM	DMSO
77-52-1	Ursolic acid	456.7	10mM	DMSO
35959-08-1	3-Acetoxy-11-ursen-28,13-olide	496.721	10mM	DMSO
35959-05-8	3-Hydroxy-11-ursen-28,13-olide	454.684	10mM	DMSO
13849-91-7	Pomolic acid	472.7	10mM	DMSO
473-98-3	Betulin	442.717	10mM	DMSO
472-15-1	Betulinic acid	456.7	10mM	DMSO
149751-81-5	Tereticornate A	630.853	10mM	DMSO
7372-30-7	Acetylursolic acid	498.737	10mM	DMSO
55722-32-2	Stigmasta-4,22-dien-3-one	410.675	10mM	DMSO
83-48-7	Stigmasterol	412.691	10mM	DMSO
123690-76-6	Bongardol	544.892	10mM	DMSO
65-85-0	Benzoic acid	122.121	10mM	DMSO
552-41-0	Paeonol	166.174	10mM	DMSO
99173-00-9	No	182.216	10mM	DMSO
25279-15-6	20S,24R-Epoxy-dammar-12,25-diol-3-one	474.716	10mM	DMSO
22549-21-9	Ocotillone	458.716	10mM	DMSO
6474-90-4	Tetrahydroalstonine	352.427	10mM	DMSO
474-58-8	Daucosterol	576.847	10mM	DMSO
121-34-6	Vanillic acid	168.147	10mM	DMSO
21978-49-4	Bombiprenone	603.015	10mM	DMSO
771-50-6	1H-Indole-3-carboxylic acid	161.157	10mM	DMSO

145-13-1	Pregnenolone	316.478	10mM	DMSO
111-02-4	Squalene	410.718	10mM	DMSO
479-43-6	Canthin-6-one	220.226	10mM	DMSO
28594-00-5	22-Dehydroclerosteryl acetate	452.712	10mM	DMSO
26187-80-4	Foliamenthoic acid	184.232	10mM	DMSO
38990-03-3	Gelsevirine	352.427	10mM	DMSO
6882-99-1	Sempervirine	272.344	10mM	DMSO
36450-02-9	6-Hydroxystigmast-4-en-3-one	428.69	10mM	DMSO
5081-51-6	Vasicinol	204.225	10mM	DMSO
51419-51-3	Odonicin	430.491	10mM	DMSO
66178-02-7	Malic acid 4-Me ester	148.114	10mM	DMSO
1058-61-3	Sitostenone	412.691	10mM	DMSO
110414-77-2	Gelsemiol	200.232	10mM	DMSO
22255-40-9	Loganic acid	376.356	10mM	DMSO
122872-03-1	2-Desoxy-4-epi-pulchellin	250.333	10mM	DMSO
No	4-Acetoxy-11(13)-pseudoguaien-12,8-olide	292.37	10mM	DMSO
1187925-31-0	Carabrolactone B	266.333	10mM	DMSO
124111-47-3	2-Hydroxytetracosanoic acid ethyl ester	412.689	10mM	DMSO
5231-60-7	Vindorosine	426.505	10mM	DMSO
126724-95-6	Tilifodiolide	336.338	10mM	DMSO
21671-00-1	Shoreic acid	474.716	10mM	DMSO
20283-92-5	Rosmarinic acid	360.315	10mM	DMSO
62218-55-7	4-Hydroxy-2,6,6-trimethyl-1-cyclohexenecarboxylic acid	184.232	10mM	DMSO
1187925-30-9	Carabrolactone A	282.332	10mM	DMSO
115321-32-9	Isosalvipuberulin	334.322	10mM	DMSO
2061-64-5	Ergosterol peroxide	428.647	10mM	DMSO
41590-29-8	4,R-ajmalicine N-oxide	368.426	10mM	DMSO
263844-80-0	3-Acetoxy-27-hydroxy-20(29)-lupen-28-oic acid methyl este	528.763	10mM	DMSO
486-64-6	Vasicinone	202.209	10mM	DMSO
80787-59-3	1-Hydroxycanthin-6-one	236.226	10mM	DMSO
84745-95-9	Eriocalyxin B	344.402	10mM	DMSO
28593-92-2	Docosyl caffeate	488.742	10mM	DMSO
1159579-44-8	Alstonic acid A	456.7	10mM	DMSO
115334-05-9	Dihydroniloticin	458.716	10mM	DMSO
60796-64-7	Norbraylin	244.243	10mM	DMSO
26585-14-8	Crenatine	226.274	10mM	DMSO
442-51-3	Harmine	212.247	10mM	DMSO
18786-24-8	Serpentine hydrochloride	384.856	10mM	DMSO
480-10-4	Astragalin	448.377	10mM	DMSO
No	7-Geranyloxy-5-methoxycoumarin	328.402	10mM	DMSO
89915-39-9	Beta-Carboline-1-propanoic acid	240.257	10mM	DMSO
96850-29-2	Maoecrystal B	388.454	10mM	DMSO
304642-94-2	Xerophilusin G	422.469	10mM	DMSO
2239-24-9	Serratenediol	442.717	10mM	DMSO
3984-73-4	Methyllinderone	300.306	10mM	DMSO
No	8-Geranyloxy-5,7-dimethoxycoumarin	358.428	10mM	DMSO
210108-87-5	2,5,14-Triacetoxy-3-benzoyloxy-8,15-dihydroxy-7-isobutyro	807.879	10mM	DMSO
981-15-7	Ailanthone	376.4	10mM	DMSO
60796-65-8	5,7,8-Trimethoxycoumarin	236.221	10mM	DMSO
1782-79-2	Linderone	286.279	10mM	DMSO
82467-50-3	R(+)-Gomisin M1	386.438	10mM	DMSO
210108-89-7	2,5,7,14-Tetraacetoxy-3-benzoyloxy-8,15-dihydroxy-9-nicot	779.826	10mM	DMSO
42438-78-8	Pashanone	300.306	10mM	DMSO
480-37-5	Pinostrobin	270.28	10mM	DMSO
96917-26-9	Artanin	290.311	10mM	DMSO
137182-37-7	Toddalosin	562.607	10mM	DMSO
4547-24-4	Corosolic acid	472.7	10mM	DMSO
27741-01-1	Geniposidic acid	374.34	10mM	DMSO

19956-54-8	Methyllucidone	270.28	10mM	DMSO
1158845-78-3	N^1-methoxymethyl picrinine	382.453	10mM	DMSO
480-39-7	Pinocembrin	256.253	10mM	DMSO
3162-45-6	5,7-Dihydroxy-6,8-dimethoxyflavone	314.289	10mM	DMSO
480-20-6	Aromadendrin	288.252	10mM	DMSO
102519-34-6	2,3,23-Trihydroxy-12-oleanen-28-oic acid	488.699	10mM	DMSO
66322-34-7	Dihydroguaiaretic acid	330.418	10mM	DMSO
1169805-98-4	Sculponeatin N	404.583	10mM	DMSO
20045-06-1	Picralinal	366.41	10mM	DMSO
530-53-0	Deoxyvasicinone	186.21	10mM	DMSO
85287-60-1	Sculponeatin A	360.401	10mM	DMSO
19956-53-7	Lucidone	256.253	10mM	DMSO
56324-22-2	19,20-(E)-Isovallesamine	340.416	10mM	DMSO
545-24-4	5-Glutinen-3-ol	426.717	10mM	DMSO
1195233-59-0	Ethyllucidone	284.307	10mM	DMSO
670257-89-3	Pepluanin A	821.863	10mM	DMSO
191545-24-1	Epieriocalyxin A	344.402	10mM	DMSO
140-10-3	Cinnamic acid	148.159	10mM	DMSO
18956-15-5	Pinostrobin chalcone	270.28	10mM	DMSO
41059-80-7	Lipiferolide	306.354	10mM	DMSO
128397-09-1	Hyptadienic acid	470.684	10mM	DMSO
210108-86-4	2,5,7,8,9,14-Hexaacetoxy-3-benzoyloxy-15-hydroxy-jatroph	758.805	10mM	DMSO
919120-78-8	19-[(beta-D-glucopyranosyl)oxy]-19-oxo-ent-labda-8(17),13	494.575	10mM	DMSO
475-75-2	Liriodenine	275.258	10mM	DMSO
99882-10-7	Kaempferol 3-O-alfa-L-arabinoside	418.351	10mM	DMSO
210108-88-6	2,5,9,14-Tetraacetoxy-3-benzoyloxy-8,15-dihydroxy-7-isob	744.822	10mM	DMSO
117-10-2	Dantron	240.211	10mM	DMSO
61597-83-9	13-Hydroxy-8,11,13-podocarpatrien-18-oic acid	274.355	10mM	DMSO
304-21-2	Harmaline	214.263	10mM	DMSO
4684-32-6	Picrinine	338.4	10mM	DMSO
28254-53-7	Reynosin	248.318	10mM	DMSO
477529-70-7	Sculponeatin K	346.417	10mM	DMSO
36417-86-4	N-p-trans-Coumaroyltyramine	283.322	10mM	DMSO
1177-14-6	DL-Syringaresinol	418.437	10mM	DMSO
263844-79-7	3,27-Dihydroxy-20(29)-lupen-28-oic acid methyl ester	486.726	10mM	DMSO
1159579-45-9	Alstonic acid B	454.684	10mM	DMSO
19013-03-7	Eupatoriochromene	218.248	10mM	DMSO
No	3,4-O-Isopropylidene-2-methylbutane-1,2,3,4-tetrol	176.21	10mM	DMSO
210108-91-1	5,8,9,10,14-Pentaacetoxy-3-benzoyloxy-15-hydroxypepluan	700.769	10mM	DMSO
123-08-0	4-Hydroxybenzaldehyde	122.121	10mM	DMSO
866111-14-0	Clausine Z	227.215	10mM	DMSO
No	2-methylbutane-1,2,3,4-tetrol	136.146	10mM	DMSO
6601-62-3	Cirsimaritin	314.289	10mM	DMSO
6483-15-4	Sophocarpine	246.348	10mM	DMSO
67667-62-3	De-O-methylacetovanillochromene	218.248	10mM	DMSO
59979-57-6	Tagitinin F	348.39	10mM	DMSO
20628-09-5	Encecalin	232.275	10mM	DMSO
94285-22-0	Acetyldihydromicromelin A	332.305	10mM	DMSO
109794-97-0	Harmalidine	254.327	10mM	DMSO
623-05-2	4-Hydroxybenzyl alcohol	124.137	10mM	DMSO
93710-27-1	Leuconolam	326.39	10mM	DMSO
1169806-02-3	Sculponeatic acid	470.684	10mM	DMSO
30484-88-9	Mearnsitrin	478.403	10mM	DMSO
521-34-6	Sciadopitysin	580.538	10mM	DMSO
54835-70-0	Roseoside	386.437	10mM	DMSO
1570-09-8	5,7-Dihydroxy-3,4',8-trimethoxyflavone	344.315	10mM	DMSO
52932-74-8	Neosophoramine	244.332	10mM	DMSO
77784-22-6	Dehydrocrebanine	337.369	10mM	DMSO

570-72-9	Stigmasta-5,8-dien-3-ol	412.691	10mM	DMSO
26904-64-3	Sophocarpidine	262.347	10mM	DMSO
30244-37-2	1-Hydroxybaccatin I	652.683	10mM	DMSO
10284-63-6	D-Pinitol	194.182	10mM	DMSO
7374-79-0	Acanthoside B	580.578	10mM	DMSO
100201-57-8	2,3-dihydroxy-3-(4-hydroxyphenyl)propanoic acid	198.173	10mM	DMSO
1169806-00-1	Sculponeatin O	440.615	10mM	DMSO
59979-61-2	Tagitinin A	368.421	10mM	DMSO
57627-75-5	3-Hydroxy-4-methoxy-benzenepropanol	182.216	10mM	DMSO
550-90-3	Lupanine	248.364	10mM	DMSO
125288-25-7	2,4,6-Trimethoxyphenol 1-O-beta-D-glucopyranoside	346.33	10mM	DMSO
69251-96-3	(+)-Pinoresinol-4-O-beta-D-glucopyranoside	520.526	10mM	DMSO
6882-68-4	Sophoridine	248.364	10mM	DMSO
531-29-3	Coniferin	342.341	10mM	DMSO
10210-17-0	3-(4-Hydroxyphenyl)-1-propanol	152.19	10mM	DMSO
27548-93-2	Baccatin III	586.627	10mM	DMSO
61152-62-3	4-(3,4-Dihydroxyphenyl)-2-butanone	180.2	10mM	DMSO
3602-54-8	2,4-Dihydroxy-6-methoxyacetophenone	182.173	10mM	DMSO
479-90-3	Artemetin	388.368	10mM	DMSO
39815-40-2	Epitulipinolide diepoxide	322.353	10mM	DMSO
99694-90-3	Scholaricine	356.416	10mM	DMSO
17912-87-7	Myricitrin	464.376	10mM	DMSO
517-63-5	Stephanine	309.359	10mM	DMSO
705973-69-9	14beta-Benzoyloxy-2-deacetylbaccatin VI	730.752	10mM	DMSO
203524-64-5	2,4,6,6-Tetramethyl-3(6H)-pyridinone	151.206	10mM	DMSO
154-23-4	Catechin	290.268	10mM	DMSO
153229-31-3	Taxayunnansin A	672.716	10mM	DMSO
603-56-5	Chrysosplenetin	374.341	10mM	DMSO
13408-56-5	Ponasterone A	464.635	10mM	DMSO
15085-71-9	Micromelin	288.252	10mM	DMSO
25127-29-1	Crebanine	339.385	10mM	DMSO
99-96-7	4-Hydroxybenzoic acid	138.121	10mM	DMSO
34086-50-5	Alpinumisoflavone	336.338	10mM	DMSO
5257-08-9	Glycozolinine	197.233	10mM	DMSO
4090-18-0	Sinoacutine	327.374	10mM	DMSO
6054-10-0	Braylin	258.269	10mM	DMSO
23624-21-7	Putraflavone	566.511	10mM	DMSO
126594-73-8	Vallesamine N-oxide	356.416	10mM	DMSO
501-36-0	Resveratrol	228.243	10mM	DMSO
625096-18-6	Viniferol D	680.698	10mM	DMSO
32981-86-5	10-Deacetylbaccatin III	544.59	10mM	DMSO
62218-08-0	Viniferin	454.471	10mM	DMSO
No	No	416.378	10mM	DMSO
98770-70-8	3-(4-Hydroxy-3-methoxyphenyl)propyl tetracosanoate	532.838	10mM	DMSO
18457-44-8	Taxinine B	664.739	10mM	DMSO
191547-12-3	2-Deacetoxytaxinine B	606.703	10mM	DMSO
62218-13-7	alfa-Viniferin	678.682	10mM	DMSO
85699-62-3	alfa-Conidendrin	356.369	10mM	DMSO
82425-45-4	Gomisin M2	386.438	10mM	DMSO
129724-83-0	3-Hydroxylanost-9(11),24-dien-26-oic acid	456.7	10mM	DMSO
107783-45-9	Coccinic acid	454.684	10mM	DMSO
29700-22-9	Oxyresveratrol	244.243	10mM	DMSO
10173-01-0	Jaceidin	360.315	10mM	DMSO
78432-78-7	19-Hydroxybaccatin III	602.626	10mM	DMSO
50461-86-4	Araneosol	374.341	10mM	DMSO
6750-60-3	Spathulenol	220.35	10mM	DMSO
35796-71-5	Zapoterin	470.512	10mM	DMSO
17635-59-5	Dihydropinosylvin methyl ether	228.286	10mM	DMSO

35302-70-6	(E)-3-Hydroxy-5-methoxystilbene	226.27	10mM	DMSO
1189362-86-4	1,3-Dihydroxy-4-methoxy-10-methylacridin-9(10H)-one	271.268	10mM	DMSO
3443-28-5	Prudomestin	330.289	10mM	DMSO
740810-42-8	Cudratricusxanthone A	396.433	10mM	DMSO
4586-68-9	Ilicic acid	252.349	10mM	DMSO
520-18-3	Kaempferol	286.236	10mM	DMSO
120-47-8	Ethylparaben	166.174	10mM	DMSO
83864-70-4	Angeloylisogomisin O	498.565	10mM	DMSO
220935-39-7	13-O-Deacetyltaxumairol Z	604.642	10mM	DMSO
97931-41-4	Methyl 3-carbazolecarboxylate	225.243	10mM	DMSO
62393-88-8	Kadsuric acid	470.684	10mM	DMSO
5085-72-3	Friedelanol	428.733	10mM	DMSO
508-71-4	Rosenonolactone	316.435	10mM	DMSO
480-40-0	Chrysin	254.238	10mM	DMSO
95135-98-1	Artemetin acetate	430.405	10mM	DMSO
115028-67-6	Pseudolaric acid D	318.45	10mM	DMSO
150-19-6	m-Methoxyphenol	124.137	10mM	DMSO
354553-35-8	Caraphenol A	676.666	10mM	DMSO
56421-13-7	Eichlerianic acid	474.716	10mM	DMSO
62615-63-8	4,6,7-Trimethoxy-5-methylcoumarin	250.247	10mM	DMSO
24808-04-6	(-)-Epiafzelechin	274.269	10mM	DMSO
82508-33-6	Methyl pseudolarate A	402.481	10mM	DMSO
84955-05-5	Cudraxanthone B	394.417	10mM	DMSO
82508-34-7	Methylpseudolarate B	446.49	10mM	DMSO
1016260-22-2	Kadsuracoccinic acid A	468.668	10mM	DMSO
20554-84-1	Parthenolide	248.318	10mM	DMSO
33708-72-4	Pachypodol	344.315	10mM	DMSO
548-82-3	Pinobanksin	272.253	10mM	DMSO
211799-56-3	2',4',5'-Trimethoxy-2",2"-dimethylpyrano[5",6":6,7]isoflavor	394.417	10mM	DMSO
93888-59-6	9-Oxo-2,7-bisaboladien-15-oic acid	250.333	10mM	DMSO
482-39-3	Afzelin	432.378	10mM	DMSO
870456-88-5	3,23-dioxo-9,19-Cyclolanost-24-en-26-oic acid	468.668	10mM	DMSO
250293-31-3	Excavatin M	360.358	10mM	DMSO
42830-48-8	Catechin 7-xyloside	422.383	10mM	DMSO
89-84-9	2',4'-Dihydroxyacetophenone	152.147	10mM	DMSO
128255-08-3	Pachyaximine A	359.588	10mM	DMSO
3484-61-5	12-Hydroxyabietic acid	318.45	10mM	DMSO
101959-37-9	Octacosyl (E)-ferulate	586.928	10mM	DMSO
2196-18-1	beta-Hydroxypropiovanillone	196.2	10mM	DMSO
1859-87-6	Echinulin	461.639	10mM	DMSO
35833-62-6	Cabraleadiol 3-acetate	502.769	10mM	DMSO
57672-77-2	Baccatin IV	652.683	10mM	DMSO
62394-00-7	Alboctalol	488.485	10mM	DMSO
99-93-4	4'-Hydroxyacetophenone	136.148	10mM	DMSO
289054-34-8	Ergosta-5,24(28)-diene-3,7,16-triol	430.663	10mM	DMSO
51551-29-2	Neoechinulin A	323.389	10mM	DMSO
37126-91-3	Murrangatin	276.285	10mM	DMSO
60125-23-7	3-(2-Hydroxyphenyl)-2-propenal	148.159	10mM	DMSO
132342-55-3	Methyl isocostate	248.361	10mM	DMSO
522-12-3	Quercetin 3-O-alpha-L-rhamnoside	448.377	10mM	DMSO
1033288-92-4	Pre-schisanartanin B	590.659	10mM	DMSO
7432-28-2	Schizandrin	432.507	10mM	DMSO
97938-31-3	Leachianone A	438.513	10mM	DMSO
13850-16-3	Tormentic acid	488.699	10mM	DMSO
252333-71-4	9,9'-O-isopropyllidene-isolariciresinol	400.465	10mM	DMSO
3650-43-9	beta-Costic acid	234.334	10mM	DMSO
135820-80-3	Blumenol C glucoside	372.453	10mM	DMSO
114297-20-0	Soyacerebroside I	714.025	10mM	DMSO

5128-43-8	5,7-diacetoxy-3,4',8-trimethoxyflavone	428.389	10mM	DMSO
185845-89-0	Pterodontic acid	234.334	10mM	DMSO
135541-40-1	Cudraxanthone L	396.433	10mM	DMSO
109592-60-1	Pinocembrin 7-acetate	298.29	10mM	DMSO
132185-83-2	5alpha-Hydroxycostic acid	250.333	10mM	DMSO
548-83-4	Galangin	270.237	10mM	DMSO
No	No	262.344	10mM	DMSO
18422-83-8	Dihydromorin	304.252	10mM	DMSO
101312-79-2	Formosanol	372.412	10mM	DMSO
128255-16-3	Axillaridine A	462.667	10mM	DMSO
482-36-0	Hyperin	464.376	10mM	DMSO
69586-96-5	Tupichilignan A	402.438	10mM	DMSO
22798-98-7	Ecdysterone 2,3:20,22-diacetonide	560.762	10mM	DMSO
2035-15-6	(-)-Maackiain	284.263	10mM	DMSO
52117-69-8	3-O-Acetylpinobanksin	314.289	10mM	DMSO
76754-24-0	Lupalbigenin	406.471	10mM	DMSO
No	Cudraxanthone L triacetate	522.543	10mM	DMSO
2545-00-8	(+)-Afzelechin	274.269	10mM	DMSO
94285-06-0	Dihydromicromelin B	290.268	10mM	DMSO
120-08-1	Scoparone	206.195	10mM	DMSO
58865-88-6	9-Oxonerolidol	236.35	10mM	DMSO
6674-40-4	5-hydroxy-7-acetoxyflavone	296.274	10mM	DMSO
226561-02-0	Amaronol B	334.278	10mM	DMSO
27200-12-0	Ampelopsin	320.251	10mM	DMSO
132185-84-3	5beta-Hydroxycostic acid	250.333	10mM	DMSO
117-39-5	Quercetin	302.236	10mM	DMSO
705-15-7	2'-Hydroxy-5'-methoxyacetophenone	166.174	10mM	DMSO
25488-59-9	2,3-Bis(3,4-dimethoxybenzyl)butyrolactone	386.438	10mM	DMSO
73281-83-1	1,4-Dihydro-1,2-dimethyl-4-oxo-3-quinolinecarboxylic acid	217.221	10mM	DMSO
6665-78-7	5,7-Diacetoxyflavone	338.311	10mM	DMSO
22798-96-5	Ecdysterone 20,22-monoacetonide	520.698	10mM	DMSO
22139-77-1	Pinosylvin	212.244	10mM	DMSO
No	No	552.524	10mM	DMSO
632-85-9	Wogonin	284.263	10mM	DMSO
226560-96-9	Amaronol A	320.251	10mM	DMSO
659738-08-6	Micranoic acid A	344.488	10mM	DMSO
14531-52-3	Dihydropinosylvin	214.26	10mM	DMSO
3420-57-3	3-Methoxyfuran	98.0999	10mM	DMSO
60132-35-6	Pterodondiol	240.382	10mM	DMSO
132351-58-7	5-Acetoxy-7-hydroxyflavone	296.274	10mM	DMSO
No	No	270.323	10mM	DMSO
487-36-5	(+)-Pinoresinol	358.385	10mM	DMSO
152243-70-4	Uncargenin C	488.699	10mM	DMSO
23246-80-2	5,7-Diacetoxy-8-methoxyflavone	368.337	10mM	DMSO
95480-80-1	5-Hydroxy-7-acetoxy-8-methoxyflavone	326.3	10mM	DMSO
480-41-1	Naringenin	272.253	10mM	DMSO
74892-45-8	5-Acetoxymatairesinol dimethyl ether	444.474	10mM	DMSO
103553-98-6	3,7-O-Diacetylpinobanksin	356.326	10mM	DMSO
62394-04-1	Cinnzeylanol	384.464	10mM	DMSO
84-26-4	Rutaecarpine	287.315	10mM	DMSO
2215-96-5	2,6-Dimethoxy-1-acetonylquinol	226.226	10mM	DMSO
520-36-5	Apigenin	270.237	10mM	DMSO
180961-65-3	Spiramilactone B	330.418	10mM	DMSO
24164-13-4	Epitulipinolide	290.354	10mM	DMSO
1909-91-7	Isocupressic acid	320.466	10mM	DMSO
619326-74-8	Deoxycalyciphylline B	341.487	10mM	DMSO
150-86-7	Phytol	296.531	10mM	DMSO
18196-13-9	Naringenin-4',7-diacetate	356.326	10mM	DMSO

486-28-2	Fraxinol	222.194	10mM	DMSO
6545-99-9	Phebalosin	258.269	10mM	DMSO
32971-25-8	(+)-Pinoresinol diacetate	442.458	10mM	DMSO
484-12-8	Osthol	244.286	10mM	DMSO
3682-04-0	Naringenin triacetate	398.363	10mM	DMSO
154801-30-6	2-(2'-Hydroxytetracosanoylamino)-octadecane-1,3,4-triol	684.128	10mM	DMSO
82508-36-9	Demethoxydeacetoxypseudolaric acid B	376.4	10mM	DMSO
881388-87-0	Daphniyunnine A	369.497	10mM	DMSO
99026-99-0	Limonexic acid	502.51	10mM	DMSO
20248-08-2	13(18)-Oleanen-3-one	424.702	10mM	DMSO
134476-74-7	Richenoic acid	456.7	10mM	DMSO
96552-41-9	Cudraxanthone D	410.46	10mM	DMSO
40433-82-7	Pinusolidic acid	332.434	10mM	DMSO
520-28-5	Tectochrysin	268.264	10mM	DMSO
559-74-0	Friedelin	426.717	10mM	DMSO
501-98-4	trans-4-Hydroxycinnamic acid	164.158	10mM	DMSO
16198-01-9	Catechin pentaacetate	500.451	10mM	DMSO
53846-49-4	Flavaprin	502.51	10mM	DMSO
488-44-8	Allitol	182.172	10mM	DMSO
750649-07-1	Daphnilongeranin C	355.471	10mM	DMSO
53377-61-0	1,3,5-Trihydroxy-4-prenylxanthone	312.317	10mM	DMSO
480-18-2	Taxifolin	304.252	10mM	DMSO
114531-28-1	Spiramine A	399.523	10mM	DMSO
127-40-2	Lutein	568.871	10mM	DMSO
246868-97-3	Aglinin A	490.715	10mM	DMSO
23971-42-8	Meranzin	260.285	10mM	DMSO
15051-81-7	epi-Eudesmol	222.366	10mM	DMSO
21040-64-2	Spiradine F	399.523	10mM	DMSO
27832-84-4	Serratenediol diacetate	526.79	10mM	DMSO
67253-01-4	Cabraleadiol	460.732	10mM	DMSO
548-89-0	Gyrophoric acid	468.41	10mM	DMSO
80604-16-6	2',5,6',7-Tetrahydroxyflavanone	288.252	10mM	DMSO
64165-98-6	Echinoynethiophene A	230.282	10mM	DMSO
185821-32-3	2,3-Dihydroxypterodontic acid	266.333	10mM	DMSO
70110-60-0	5-Tricosyl-1,3-benzenediol	432.722	10mM	DMSO
138-59-0	Shikimic acid	174.151	10mM	DMSO
38302-15-7	Naringenin trimethyl ether	314.333	10mM	DMSO
17245-25-9	Coumurrayin	274.312	10mM	DMSO
1990-77-8	Syringaresinol diacetate	502.51	10mM	DMSO
56795-51-8	Scopoletin acetate	234.205	10mM	DMSO
34427-61-7	Ikshusterol	430.706	10mM	DMSO
568-72-9	Tanshinone II	294.344	10mM	DMSO
482-91-7	Aricine	382.453	10mM	DMSO
No	2',6',7-Triacetoxy-5-hydroxyflavanone	414.362	10mM	DMSO
109741-38-0	Murraol	260.285	10mM	DMSO
530-55-2	2,6-Dimethoxy-1,4-benzoquinone	168.147	10mM	DMSO
25532-45-0	Mayumbine	352.427	10mM	DMSO
113557-95-2	Magnoloside A	624.587	10mM	DMSO
17245-30-6	3',4',5',3,5,6,7-Heptamethoxyflavone	432.421	10mM	DMSO
508-04-3	13(18)-Oleanen-3-ol	426.717	10mM	DMSO
186374-63-0	enantio-7(11)-Eudesmen-4-ol	222.366	10mM	DMSO
67560-68-3	Lariciresinol dimethyl ether	388.454	10mM	DMSO
17391-09-2	Isocarapanaubine	428.478	10mM	DMSO
221666-27-9	(+)-Conocarpan	266.334	10mM	DMSO
1486-70-0	3-O-Methylquercetin	316.262	10mM	DMSO
260397-58-8	Dihydroisotanshinone II	278.302	10mM	DMSO
97399-93-4	Paniculidine A	231.29	10mM	DMSO
16274-11-6	Kaempferol tetraacetate	454.383	10mM	DMSO

520-26-3	Hesperidin	610.561	10mM	DMSO
35825-57-1	Cryptotanshinone	296.36	10mM	DMSO
160436-10-2	3',5,5',7-Tetrahydroxyflavanone	288.252	10mM	DMSO
143724-69-0	Kaempferol 3,4',7-triacetate	412.346	10mM	DMSO
92519-91-0	Viscidulin III	346.288	10mM	DMSO
110-17-8	Fumaric acid	116.072	10mM	DMSO
35833-69-3	Cabraleahydroxylactone	416.636	10mM	DMSO
80604-17-7	2',5,6',7-Tetraacetoxyflavanone	456.399	10mM	DMSO
69978-82-1	Isocostic acid	234.334	10mM	DMSO
53851-13-1	Caboxine A	398.452	10mM	DMSO
99946-04-0	Isoboonein	170.206	10mM	DMSO
41514-64-1	Koaburaside monomethyl ether	346.33	10mM	DMSO
20835-91-0	Stigmastane-3,5,6-triol	448.721	10mM	DMSO
82508-37-0	Deacetylpseudolaric acid A	346.417	10mM	DMSO
73354-15-1	Lariciresinol-4,4'-dimethyl ether-9-acetate	430.491	10mM	DMSO
2202-01-9	Eichlerialactone	430.62	10mM	DMSO
6426-43-3	Taraxasterol acetate	468.754	10mM	DMSO
119400-87-2	Przewalskin	272.382	10mM	DMSO
86838-54-2	2,2,5,5-Tetramethylcyclohexane-1,4-dione	168.233	10mM	DMSO
96684-81-0	Viscidulin III tetraacetate	514.435	10mM	DMSO
97399-95-6	Paniculidine C	203.28	10mM	DMSO
21634-52-6	3',4',5',3,5,7,8-Heptamethoxyflavone	432.421	10mM	DMSO
No	2alpha-Hydroxy pterodontic acid methyl ester	264.36	10mM	DMSO
102054-39-7	Arctinol B	264.363	10mM	DMSO
530-57-4	Syringic acid	198.173	10mM	DMSO
630057-39-5	Neoprzewaquinone A	556.604	10mM	DMSO
26315-07-1	22-Dehydroclerosterol	410.675	10mM	DMSO
88546-96-7	Minumicrolin	276.285	10mM	DMSO
No	3',5,5',7-Tetraacetoxyflavanone	456.399	10mM	DMSO
1186496-68-3	11-Hydroxycodaphniphylline	485.698	10mM	DMSO
71144-78-0	(E)-3-Acetoxy-5-methoxystilbene	268.307	10mM	DMSO
13241-28-6	Chrysophanol 8-O-glucoside	416.378	10mM	DMSO
No	No	414.452	10mM	DMSO
56121-42-7	Asperglaucide	444.522	10mM	DMSO
491-70-3	Luteolin	286.236	10mM	DMSO
10388-48-4	Cycloart-25-ene-3,24-diol	442.717	10mM	DMSO
13364-94-8	Exoticin	462.447	10mM	DMSO
88478-44-8	Murraxocin	304.338	10mM	DMSO
14004-35-4	Eucalyptin acetate	368.38	10mM	DMSO
No	No	468.453	10mM	DMSO
57576-31-5	(24S)-Cycloartane-3,24,25-triol 24,25-acetonide	500.796	10mM	DMSO
97399-94-5	Paniculidine B	233.306	10mM	DMSO
568-73-0	Tanshinone I	276.286	10mM	DMSO
143815-99-0	22-Dehydroclerosterol glucoside	572.816	10mM	DMSO
2308-85-2	Sitosteryl palmitate	653.116	10mM	DMSO
99-76-3	Methyl 4-hydroxybenzoate	152.147	10mM	DMSO
51650-59-0	Murrangatin diacetate	360.358	10mM	DMSO
56319-04-1	(+)-Conocarpan acetate	308.371	10mM	DMSO
51361-60-5	delta-Amyrin acetate	468.754	10mM	DMSO
80366-15-0	2',3,5,6',7-Pentahydroxyflavanone	304.252	10mM	DMSO
92519-95-4	Viscidulin I	302.236	10mM	DMSO
58546-54-6	Gomisin A	416.464	10mM	DMSO
83916-76-1	Isogomisin O	416.464	10mM	DMSO
114916-00-6	No	282.809	10mM	DMSO
121700-26-3	Moluccanin	386.352	10mM	DMSO
131652-35-2	No	294.73	10mM	DMSO
95360-22-8	Hainanmurpanin	318.321	10mM	DMSO
1486-69-7	3-O-Methylquercetin tetraacetate	484.409	10mM	DMSO

32179-18-3	1,10:4,5-Diepoxy-7(11)-germacren-8-one	250.333	10mM	DMSO
5875-49-0	Meranzin hydrate	278.3	10mM	DMSO
88585-86-8	Isomexoticin	308.326	10mM	DMSO
No	Quercitrin hexaacetate	700.597	10mM	DMSO
121700-27-4	Moluccanin diacetate	470.425	10mM	DMSO
6665-67-4	4',5-Dihydroxyflavone	254.238	10mM	DMSO
61276-17-3	Acteoside	624.587	10mM	DMSO
202846-95-5	8-Hydroxy-3,5,7,3',4',5'-hexamethoxyflavone	418.394	10mM	DMSO
105351-70-0	Tanshinlactone	264.275	10mM	DMSO
92233-55-1	Syringin pentaacetate	582.55	10mM	DMSO
99891-77-7	Isoboonein acetate	212.242	10mM	DMSO
165074-00-0	6-Epidemethylesquirolin D	348.433	10mM	DMSO
78876-52-5	No	248.728	10mM	DMSO
26905-70-4	2-(4-Chloro-3-hydroxybuten-1-yl)-5-(pentadien-1,3-yl)thio[illegible]	248.728	10mM	DMSO
78876-53-6	No	234.701	10mM	DMSO
14397-69-4	Jaceidin triacetate	486.425	10mM	DMSO
5309-35-3	9,13-Epidioxy-8(14)-abieten-18-oic acid	334.45	10mM	DMSO
17397-93-2	Tanshinone IIB	310.344	10mM	DMSO
19626-92-7	Indole-3-acrylic acid methyl ester	201.221	10mM	DMSO
95188-34-4	Sibiricin	290.311	10mM	DMSO
79955-41-2	Quinovic acid 3-O-beta-D-glucoside	648.824	10mM	DMSO
27003-73-2	(+)-Lariciresinol	360.401	10mM	DMSO
54113-95-0	15-Hydroxydehydroabietic acid	316.435	10mM	DMSO
53011-72-6	Murralongin	258.269	10mM	DMSO
483-09-0	Isorauhimbine	354.443	10mM	DMSO
53155-25-2	Euscaphic acid	488.699	10mM	DMSO
87440-75-3	Viteralone	242.27	10mM	DMSO
107870-05-3	Quinovin	632.824	10mM	DMSO
189264-47-9	Cleroindicin F	154.163	10mM	DMSO
102227-61-2	Epoxyparvinolide	250.333	10mM	DMSO
885315-96-8	Kongensin A	374.471	10mM	DMSO
59219-65-7	Darutoside	484.623	10mM	DMSO
52589-11-4	Phellamurin	518.51	10mM	DMSO
111441-88-4	Pinocembrin diacetate	340.327	10mM	DMSO
87205-99-0	15,16-Dihydrotanshinone I	278.302	10mM	DMSO
107389-91-3	Cleroindicin B	158.195	10mM	DMSO
61263-49-8	Vitexilactone	378.502	10mM	DMSO
289056-24-2	3,7,16-Trihydroxystigmast-5-ene	446.705	10mM	DMSO
118916-57-7	Balanophonin	356.369	10mM	DMSO
2880-49-1	Heraclenin	286.279	10mM	DMSO
7437-55-0	8-Geranyloxypsoralen	338.397	10mM	DMSO
53600-24-1	1-Hydroxyrutaecarpine	303.315	10mM	DMSO
32383-76-9	Medicarpin	270.28	10mM	DMSO
520-12-7	Pectolinarigenin	314.289	10mM	DMSO
5940-00-1	Darutigenol	322.482	10mM	DMSO
125072-68-6	No	306.354	10mM	DMSO
No	Kongensin A acetate	416.507	10mM	DMSO
124727-10-2	Quinovic acid 3-O-(6-deoxy-beta-D-glucopyranoside) 28-O[illegible]	794.965	10mM	DMSO
1447-88-7	Hispidulin	300.263	10mM	DMSO
531-44-2	Scopolin	354.309	10mM	DMSO
63543-52-2	24,25-Epoxy-dammar-20(21)-en-3-one	440.701	10mM	DMSO
98755-25-0	Isothymusin	330.289	10mM	DMSO
482-44-0	Imperatorin	270.28	10mM	DMSO
581-31-7	Suberosin	244.286	10mM	DMSO
160927-81-1	Villosin C	360.401	10mM	DMSO
143839-02-5	2,24-Dihydroxyursolic acid	488.699	10mM	DMSO
42438-89-1	Pinostilbene	242.27	10mM	DMSO
55050-69-6	Dammar-20(21)-en-3,24,25-triol	460.732	10mM	DMSO

37921-38-3	Cimifugin	306.311	10mM	DMSO
145918-59-8	7,8,9,9-Tetradehydroisolariciresinol	356.369	10mM	DMSO
99353-00-1	Methyl rosmarinate	374.341	10mM	DMSO
464-98-2	Pseudotaraxasterol	426.717	10mM	DMSO
483-66-9	Sphondin	216.19	10mM	DMSO
189264-44-6	Cleroindicin C	156.179	10mM	DMSO
559-70-6	beta-Amyrin	426.717	10mM	DMSO
482-27-9	Isopimpinellin	246.215	10mM	DMSO
56317-15-8	5,6,7,8-Tetramethoxycoumarin	266.247	10mM	DMSO
1059-14-9	Taraxasterol	426.717	10mM	DMSO
624-45-3	Methyl levulinate	130.142	10mM	DMSO
1188282-01-0	16-O-Acetyldarutigenol	364.519	10mM	DMSO
22318-10-1	Evodol	484.495	10mM	DMSO
1188281-98-2	ent-14,16-Epoxy-8-pimarene-3,15-diol	320.466	10mM	DMSO
545-47-1	Lupeol	426.717	10mM	DMSO
225110-25-8	Falcarindiol	260.371	10mM	DMSO
580-72-3	Matairesinol	358.385	10mM	DMSO
17388-39-5	Swertiamarin	374.34	10mM	DMSO
115458-73-6	Glaucin B	528.548	10mM	DMSO
6035-49-0	6,7,8-Trimethoxycoumarin	236.221	10mM	DMSO
142299-73-8	Teuvincenone H	356.369	10mM	DMSO
514-07-8	Taraxerone	424.702	10mM	DMSO
71933-54-5	Sarmentosin	275.255	10mM	DMSO
72755-20-5	Odorine	300.395	10mM	DMSO
298-81-7	Xanthotoxin	216.19	10mM	DMSO
83905-81-1	12-Acetoxyabietic acid	360.487	10mM	DMSO
989-61-7	Limonol	472.527	10mM	DMSO
482-38-2	Kaempferitrin	578.519	10mM	DMSO
52483-19-9	5-(Z-heptadec-8-enyl) resorcinol	346.547	10mM	DMSO
17020-04-1	Bauerenol acetate	468.754	10mM	DMSO
4439-68-3	Norkhellol	232.189	10mM	DMSO
28217-60-9	Phlorin	288.251	10mM	DMSO
72755-22-7	Odorinol	316.395	10mM	DMSO
52992-82-2	Acetylisocupressic acid	362.503	10mM	DMSO
151561-88-5	ent-3-Oxokauran-17-oic acid	318.45	10mM	DMSO
165197-71-7	Cleroindicin E	158.195	10mM	DMSO
189264-45-7	Cleroindicin D	172.178	10mM	DMSO
14050-92-1	Methyl chanofruticosinate	410.463	10mM	DMSO
57096-02-3	4'-Hydroxywogonin	300.263	10mM	DMSO
24022-13-7	Agathadiol diacetate	390.556	10mM	DMSO
150821-16-2	2,3,24-Trihydroxyolean-12-en-28-oic acid	488.699	10mM	DMSO
36519-42-3	Serpentinine	684.823	10mM	DMSO
144765-80-0	Brachynoside heptaacetate	946.897	10mM	DMSO
524-15-2	gamma-Fagarine	229.231	10mM	DMSO
89786-83-4	2,3,24-Trihydroxy-12-ursen-28-oic acid	488.699	10mM	DMSO
82-57-5	Visnagin	230.216	10mM	DMSO
439-89-4	Gentianine	175.184	10mM	DMSO
480-44-4	Acacetin	284.263	10mM	DMSO
32451-85-7	20(29)-Lupene-3,23-diol	442.717	10mM	DMSO
103974-74-9	Esculentic acid	488.699	10mM	DMSO
1188281-99-3	7-Hydroxydarutigenol	338.482	10mM	DMSO
135683-73-7	ent-3-Oxokaurane-16,17-diol	320.466	10mM	DMSO
121521-90-2	Salvianolic acid B	718.614	10mM	DMSO
93675-85-5	Rengyol	160.211	10mM	DMSO
65236-62-6	Dehydrocrenatidine	254.284	10mM	DMSO
76948-72-6	Cleomiscosin A	386.352	10mM	DMSO
31575-93-6	Heraclenol	304.295	10mM	DMSO
131-07-7	Serpentine	348.395	10mM	DMSO

508-02-1	Oleanolic acid	456.7	10mM	DMSO
189322-69-8	3'-Methoxyrocaglamide	535.585	10mM	DMSO
84573-16-0	Rocaglamide	505.559	10mM	DMSO
11027-63-7	Agnuside	466.435	10mM	DMSO
63543-53-3	24,25-Dihydroxydammar-20-en-3-one	458.716	10mM	DMSO
49624-66-0	Angelicain	292.284	10mM	DMSO
18642-44-9	Actein	676.834	10mM	DMSO
4373-41-5	Crategolic acid	472.7	10mM	DMSO
483-14-7	Tetrahydropalmatine	355.428	10mM	DMSO
18110-86-6	5-Hydroxy-4-methoxycanthin-6-one	266.252	10mM	DMSO
970-73-0	3,3',4',5,5',7-Hexahydroxyflavan	306.267	10mM	DMSO
26791-73-1	Xanthatin	246.302	10mM	DMSO
569-80-2	Penduletin	344.315	10mM	DMSO
66465-24-5	Kaempferol 3-O-(6"-O-acetyl)glucoside-7-O-rhamnoside	636.555	10mM	DMSO
52659-56-0	Kirenol	338.482	10mM	DMSO
3464-66-2	1-Methoxycarbonyl-beta-carboline	226.231	10mM	DMSO
41447-15-8	(-)-N-Methylsedridine	157.253	10mM	DMSO
41447-16-9	(+)-N-Methylallosedridine	157.253	10mM	DMSO
60761-79-7	ent-kauran-17,19-dioic acid	334.4	10mM	DMSO
21887-01-4	Horminone	332.434	10mM	DMSO
32207-10-6	Heraclenol 3'-O-beta-D-glucopyranoside	466.435	10mM	DMSO
1188282-00-9	9-Hydroxydarutigenol	338.482	10mM	DMSO
606125-07-9	Momor-cerebroside I	844.253	10mM	DMSO
1782-65-6	Hardwickiic acid	316.435	10mM	DMSO
60807-25-2	4-Methoxy-1-methoxycarbonyl-beta-carboline	256.257	10mM	DMSO
5973-06-8	beta-Amyrin palmitate	665.126	10mM	DMSO
171674-89-8	Nitidanin	404.41	10mM	DMSO
2239-88-5	3,3'-Di-O-methylellagic acid	330.246	10mM	DMSO
19103-54-9	Salvigenin	328.316	10mM	DMSO
31008-18-1	Magnolin	416.464	10mM	DMSO
26585-13-7	Dehydrocrenatine	224.258	10mM	DMSO
52213-27-1	2,3-Dihydroxy-12-ursen-28-oic acid	472.7	10mM	DMSO
340702-68-3	No	852.275	10mM	DMSO
1617-49-8	2,3,8-Tri-O-methylellagic acid	344.272	10mM	DMSO
131-03-3	alpha-Yohimbine	354.443	10mM	DMSO
189322-67-6	3'-Hydroxyrocaglamide	521.558	10mM	DMSO
549-84-8	beta-Yohimbine	354.443	10mM	DMSO
82-02-0	Khellin	260.242	10mM	DMSO
87592-77-6	Smyrindioloside	424.399	10mM	DMSO
1188282-02-1	15,16-Di-O-acetyldarutoside	568.696	10mM	DMSO
41653-72-9	Lacinilene C	246.302	10mM	DMSO
57457-97-3	p-Menth-8-ene-1,2-diol	170.249	10mM	DMSO
6610-56-6	Glochidiol	442.717	10mM	DMSO
147059-46-9	Rocaglaol	434.481	10mM	DMSO
390362-53-5	Buxbodine D	426.678	10mM	DMSO
80151-89-9	Methyl demethoxycarbonylchanofruticosinate	352.427	10mM	DMSO
927812-23-5	Glochicoccin D	436.409	10mM	DMSO
111-20-6	Decanedioic acid	202.248	10mM	DMSO
960589-81-5	ent-17-Hydroxykauran-3-one	304.467	10mM	DMSO
153483-31-9	Xanthinin	306.354	10mM	DMSO
100079-34-3	7-Hydroxy-2',5,8-trimethoxyflavanone	330.332	10mM	DMSO
80554-58-1	1-Methyl-2-heptyl-4(1H)-quinolinone	257.371	10mM	DMSO
22048-98-2	1-Methyl-2-pentyl-4(1H)-quinolinone	229.317	10mM	DMSO
76792-94-4	3-Hydroxy-4',5,7-trimethoxyflavanone	330.332	10mM	DMSO
1007125-14-5	3-(Hydroxymethyl)cyclopentanol	116.158	10mM	DMSO
18110-87-7	4,5-Dimethoxycanthin-6-one	280.278	10mM	DMSO
140631-27-2	12-Hydroxyjasmonic acid	226.269	10mM	DMSO
193969-08-3	10-O-Vanilloylaucubin	496.461	10mM	DMSO

24513-41-5	Monomethyl kolavate	348.476	10mM	DMSO
73815-21-1	2,6-Dimethyl-7-octene-2,3,6-triol	188.264	10mM	DMSO
19716-66-6	Pseudopalmatine	352.404	10mM	DMSO
18524-94-2	Loganin	390.382	10mM	DMSO
63399-37-1	3-Acetoxy-8(17),13E-labdadien-15-oic acid	362.503	10mM	DMSO
23526-45-6	Vomifoliol	224.296	10mM	DMSO
19833-13-7	Erythrodiol 3-palmitate	681.126	10mM	DMSO
41756-77-8	Dihydrophaseic acid	282.332	10mM	DMSO
131-12-4	Pimpinellin	246.215	10mM	DMSO
582315-55-7	Betulin palmitate	681.126	10mM	DMSO
113681-11-1	3-(hydroxymethyl)cyclopentanone	114.142	10mM	DMSO
86654-26-4	7-Hydroxy-3-prenylcoumarin	230.259	10mM	DMSO
488-82-4	D-arabinitol	152.146	10mM	DMSO
1617-70-5	Lupenone	424.702	10mM	DMSO
100234-62-6	Picrasidine J	242.273	10mM	DMSO
155348-06-4	p-Menthan-1,3,8-triol	188.264	10mM	DMSO
58493-71-3	ent-16beta,17-Isopropylidenedioxykaurane	346.547	10mM	DMSO
64998-19-2	7alpha-Hydroxystigmasterol	428.69	10mM	DMSO
831-61-8	Ethyl gallate	198.173	10mM	DMSO
853267-91-1	Hythiemoside A	526.659	10mM	DMSO
20649-43-8	Sinapaldehyde	208.211	10mM	DMSO
28587-43-1	7-Geranyloxy-6-methoxycoumarin	328.402	10mM	DMSO
100079-39-8	2',4'-Dihydroxy-2,3',6'-trimethoxychalcone	330.332	10mM	DMSO
942-24-5	Methyl 3-indolecarboxylate	175.184	10mM	DMSO
112503-87-4	Picrasidine S	509.576	10mM	DMSO
638203-32-4	Phyllostadimer A	810.837	10mM	DMSO
4382-56-3	Perakine	350.411	10mM	DMSO
61012-31-5	Ferulamide	193.199	10mM	DMSO
7559-04-8	alpha-Tocopherolquinone	446.705	10mM	DMSO
1207181-63-2	Scutebata G	679.755	10mM	DMSO
29424-96-2	4',7-Di-O-methylnaringenin	300.306	10mM	DMSO
84812-00-0	3,4-Dimethoxyphenyl beta-D-glucoside	316.304	10mM	DMSO
25522-33-2	Isoferulic acid	194.184	10mM	DMSO
132915-47-0	9-Hydroxy-13E-labden-15-oic acid	322.482	10mM	DMSO
268214-50-2	Coronalolide methyl ester	496.678	10mM	DMSO
64790-68-7	Heraclenol acetonide	344.358	10mM	DMSO
3301-61-9	ent-16beta,17-Dihydroxy-19-kauranoic acid	336.466	10mM	DMSO
126176-79-2	Icariside E5	522.542	10mM	DMSO
3489-06-3	Vineridine	398.452	10mM	DMSO
132339-37-8	4-Epialyxialactone	200.232	10mM	DMSO
70897-14-2	No	340.37	10mM	DMSO
194145-29-4	Rivulobirin B	432.336	10mM	DMSO
19013-07-1	Demethoxyencecalin	202.249	10mM	DMSO
256925-92-5	Cimiracemoside C	620.814	10mM	DMSO
53823-02-2	Onitin	248.318	10mM	DMSO
63399-38-2	Alepterolic acid	320.466	10mM	DMSO
884-35-5	Methyl syringate	212.199	10mM	DMSO
170809-24-2	No	318.45	10mM	DMSO
17650-84-9	Nicotiflorin	594.518	10mM	DMSO
24480-45-3	Bryonolic acid	456.7	10mM	DMSO
55732-36-0	No	202.248	10mM	DMSO
94942-49-1	6-Methoxynaringenin	302.279	10mM	DMSO
437-64-9	Genkwanin	284.263	10mM	DMSO
121748-11-6	threo-1-C-Syringylglycerol	244.241	10mM	DMSO
1207181-62-1	Scutebata F	555.616	10mM	DMSO
2009-24-7	Xanthotoxol	202.163	10mM	DMSO
425680-98-4	8-Hydroxy-5-O-beta-D-glucopyranosylpsoralen	380.303	10mM	DMSO
299159-90-3	7-Hydroxy-6-methoxy-3-prenylcoumarin	260.285	10mM	DMSO

132237-63-9	Alyxialactone	200.232	10mM	DMSO
35761-54-7	Cabraleone	458.716	10mM	DMSO
55481-86-2	Isosaxalin	322.74	10mM	DMSO
22934-99-2	Desmethoxycentaureidin	330.289	10mM	DMSO
471-69-2	Dipterocarpol	442.717	10mM	DMSO
90-01-7	Salicyl alcohol	124.137	10mM	DMSO
93-35-6	Umbelliferone	162.142	10mM	DMSO
78510-19-7	N-trans-Feruloyl-3-methoxytyramine	343.374	10mM	DMSO
87827-55-2	Alismol	220.35	10mM	DMSO
58-96-8	Uridine	244.201	10mM	DMSO
888482-17-5	11,12-De(methylenedioxy)danuphylline	426.462	10mM	DMSO
68799-38-2	4-Oxobedfordiaic acid	250.333	10mM	DMSO
145163-97-9	3beta-Hydroxyporiferast-5-en-7-one	428.69	10mM	DMSO
310433-44-4	Echinophyllin C	331.449	10mM	DMSO
111917-59-0	Adenanthin	490.543	10mM	DMSO
84575-10-0	Cleomiscosin C	416.378	10mM	DMSO
929881-46-9	Cucumegastigmane I	240.296	10mM	DMSO
53319-52-1	Isogosferol	286.279	10mM	DMSO
27994-11-2	Cimigenoside	620.814	10mM	DMSO
149252-87-9	1-Dehydroxy-23-deoxojessic acid	470.727	10mM	DMSO
85889-15-2	prim-O-Glucosylangelicain	454.425	10mM	DMSO
123135-05-7	Uncaric acid	488.699	10mM	DMSO
931114-98-6	(S,E)-Deca-2,9-dien-4,6-diyn-1,8-diol	162.185	10mM	DMSO
35214-82-5	Neobyakangelicol	316.305	10mM	DMSO
112408-71-6	2',5,7-Trihydroxy-8-methoxyflavanone	302.279	10mM	DMSO
925932-10-1	Secaubrytriol	490.715	10mM	DMSO
476-66-4	Ellagic acid	302.193	10mM	DMSO
1207181-58-5	Scutebata B	633.685	10mM	DMSO
765316-44-7	Heraclenol 3'-O-[beta-D-apiofuranosyl-(1-6)-beta-D-glucopy	598.55	10mM	DMSO
102919-76-6	Scutellaric acid	472.7	10mM	DMSO
38710-26-8	Seneciphylline N-oxide	349.378	10mM	DMSO
2465-11-4	Stellasterol	398.664	10mM	DMSO
268214-52-4	Coronalolic acid	470.684	10mM	DMSO
108124-75-0	6-(beta-D-glucopyranosyloxy)-Salicylic acid methyl ester	330.287	10mM	DMSO
117591-81-8	Coronarin E	284.436	10mM	DMSO
205115-75-9	Lansiumarin C	354.396	10mM	DMSO
114567-47-4	Ganoderiol F	454.684	10mM	DMSO
72021-23-9	Henryoside	584.523	10mM	DMSO
6871-44-9	Echitamine	385.477	10mM	DMSO
480-56-8	Lecanoric acid	318.278	10mM	DMSO
18747-42-7	Methylisopelletierine	155.237	10mM	DMSO
10351-88-9	Phyllanthin	418.523	10mM	DMSO
6519-26-2	(16R)-Dihydrositsirikine	356.459	10mM	DMSO
147-85-3	Proline	115.13	10mM	DMSO
56755-22-7	Phenylalanine betaine	207.269	10mM	DMSO
104759-35-5	Ganoderic acid S	452.669	10mM	DMSO
58115-31-4	Aurantiamide	402.486	10mM	DMSO
106623-23-8	3,10-Dihydroxy-5,11-dielmenthadiene-4,9-dione	332.434	10mM	DMSO
113626-76-9	Stigmast-4-ene-3,6-diol	430.706	10mM	DMSO
50656-77-4	Niranthin	432.507	10mM	DMSO
2571-22-4	Tutin	294.3	10mM	DMSO
6832-60-6	Imbricatolic acid	322.482	10mM	DMSO
151121-39-0	(E)-8-(6-Hydroperoxy-3,7-dimethylocta-2,7-dienyloxy)psora	370.396	10mM	DMSO
33676-00-5	Hypophyllanthin	430.491	10mM	DMSO
268214-51-3	Coronalolide	482.651	10mM	DMSO
106518-63-2	Ganodermanontriol	472.7	10mM	DMSO
176665-78-4	Ducheside A	448.334	10mM	DMSO
283174-18-5	11beta-Hydroxycedrelone	438.513	10mM	DMSO

138-52-3	Salicin	286.278	10mM	DMSO
194027-11-7	6,19-Dihydroxyurs-12-en-3-oxo-28-oic acid	486.683	10mM	DMSO
139-85-5	3,4-Dihydroxybenzaldehyde	138.121	10mM	DMSO
390362-51-3	Buxbodine B	399.609	10mM	DMSO
20649-42-7	Coniferaldehyde	178.185	10mM	DMSO
35481-77-7	Corianin	294.3	10mM	DMSO
91653-75-7	Coriatin	296.316	10mM	DMSO
481-74-3	Chrysophanol	254.238	10mM	DMSO
173075-45-1	Ganoderic acid DM	468.668	10mM	DMSO
518-82-1	Emodin	270.237	10mM	DMSO
87797-84-0	6beta-Hydroxyipolamiide	422.381	10mM	DMSO
1207181-61-0	Scutebata E	520.612	10mM	DMSO
67650-47-9	Clerodermic acid methyl ester	346.461	10mM	DMSO
905929-95-5	Scutebarbatine B	557.634	10mM	DMSO
925932-08-7	Secaubryenol	456.7	10mM	DMSO
104700-97-2	Ganoderol A	438.685	10mM	DMSO
480-81-9	Seneciphylline	333.379	10mM	DMSO
167875-39-0	Rubelloside B	794.965	10mM	DMSO
942582-15-2	Walsuronoid B	438.513	10mM	DMSO
89498-91-9	Picrasinol B	392.486	10mM	DMSO
No	Ducheside A pentaacetate	658.517	10mM	DMSO
16929-95-6	Tyromycic acid	452.669	10mM	DMSO
No	15-Ethoxychinensine A	344.488	10mM	DMSO
113808-03-0	Picrasidine T	481.522	10mM	DMSO
23141-25-5	Strictosamide	498.525	10mM	DMSO
41983-91-9	Glabranin	324.37	10mM	DMSO
237407-59-9	Rivulobirin E	590.574	10mM	DMSO
83015-88-7	1,6-Dioxaspiro[4.5]decan-2-methanol	172.222	10mM	DMSO
No	No	214.258	10mM	DMSO
76497-69-3	15,16-Dinor-8(17),11-labdadien-13-one	260.414	10mM	DMSO
2957-21-3	Sakuranetin	286.279	10mM	DMSO
160598-92-5	Villosin	300.435	10mM	DMSO
107900-76-5	Ganodermanondiol	456.7	10mM	DMSO
25018-67-1	1,2:4,5-Di-O-isopropylidene-beta-D-fructopyranose	260.284	10mM	DMSO
855-96-9	Eupatorin	344.315	10mM	DMSO
215609-93-1	23-deoxojessic acid	486.726	10mM	DMSO
70110-61-1	5-Pentacosylresorcinol	460.775	10mM	DMSO
176520-13-1	Scutebarbatine A	558.622	10mM	DMSO
481-73-2	Citreorosein	286.236	10mM	DMSO
97465-82-2	Dehydrodiconiferyl alcohol	358.385	10mM	DMSO
19417-00-6	Isohyenanchin	312.315	10mM	DMSO
152685-91-1	Cimiside B	752.928	10mM	DMSO
213905-35-2	11(S),16(R)-Dihydroxyoctadeca-9Z,17-diene-12,14-diyn-1-[illegible]	332.434	10mM	DMSO
211238-60-7	9(Z),17-Octadecadiene-12,14-diyne-1,11,16-triol	290.397	10mM	DMSO
2749-28-2	2-O-Acetyltutin	336.337	10mM	DMSO
37831-70-2	Phaseollidin	324.37	10mM	DMSO
99-50-3	3,4-Dihydroxybenzoic acid	154.12	10mM	DMSO
607-80-7		354.353	10mM	DMSO
88191-14-4	3,5,9-Trihydroxyergosta-7,22-dien-6-one	444.647	10mM	DMSO
21302-79-4	Ceanothic acid	486.683	10mM	DMSO
14858-07-2	3,5-Dihydroxyergosta-7,22-dien-6-one	428.647	10mM	DMSO
119188-38-4	Coronarin B	334.45	10mM	DMSO
1399-49-1	Globularin	492.473	10mM	DMSO
2415-24-9	Catalpol	362.329	10mM	DMSO
516-37-0	Cerevisterol	430.663	10mM	DMSO
104700-96-1	Ganoderol B	440.701	10mM	DMSO
86989-18-6	No	378.375	10mM	DMSO
No	6-O-Acetylcoriatin	338.352	10mM	DMSO

131984-82-2	3,6,19-Trihydroxy-23-oxo-12-ursen-28-oic acid	502.683	10mM	DMSO
155759-02-7	Hierochin D	344.358	10mM	DMSO
119188-33-9	Coronarin A	300.435	10mM	DMSO
18749-71-8	Sitoindoside I	815.256	10mM	DMSO
288248-46-4	Phlorigidoside B	464.418	10mM	DMSO
3681-93-4	Vitexin	432.378	10mM	DMSO
71035-06-8	Griselinoside	432.376	10mM	DMSO
465-74-7	Quinovic acid	486.683	10mM	DMSO
17306-46-6	Rhoifolin	578.519	10mM	DMSO
572-30-5	Avicularin	434.35	10mM	DMSO
60263-06-1	Jacaranone ethyl ester	196.2	10mM	DMSO
260393-05-3	Zamanic acid	618.842	10mM	DMSO
80489-65-2	Rubifolic acid	472.7	10mM	DMSO
120722-04-5	12alpha-Hydroxyevodol	500.495	10mM	DMSO
125537-92-0	3',5,5',7-Tetrahydroxy-4',6-dimethoxyflavone	346.288	10mM	DMSO
165338-27-2	Hemiphroside A	668.64	10mM	DMSO
No	No	416.4	10mM	DMSO
604-99-9	Tombozine	294.391	10mM	DMSO
931116-24-4	(R,E)-Deca-2-ene-4,6-diyn-1,8-diol	164.201	10mM	DMSO
154461-65-1	Sinapaldehyde glucoside	370.351	10mM	DMSO
63034-29-7	Hexacosyl (E)-ferulate	558.875	10mM	DMSO
165338-28-3	Hemiphroside B	682.623	10mM	DMSO
1093207-99-8	3-Hydroxy-4,15-dinor-1(5)-xanthen-12,8-olide	224.296	10mM	DMSO
3301-49-3	Kumatakenin	314.289	10mM	DMSO
1207181-57-4	Scutebata A	632.697	10mM	DMSO
No	2,6-Di-O-acetylisohyenanchin	396.388	10mM	DMSO
51225-30-0	Wighteone	338.354	10mM	DMSO
261768-88-1	3,19-Dihydroxy-6,23-dioxo-12-ursen-28-oic acid	500.667	10mM	DMSO
47418-70-2	N-Methylsarpagine methosalt	339.451	10mM	DMSO
890317-92-7	erythro-Guaiacylglycerol beta-coniferyl ether	376.4	10mM	DMSO
1254-85-9	Cedrelone	422.513	10mM	DMSO
511-01-3	alpha-Onocerol	442.717	10mM	DMSO
112137-81-2	Ikshusterol 3-O-beta-D-glucopyranoside	592.847	10mM	DMSO
849245-34-7	Chinensine B	316.435	10mM	DMSO
104055-76-7	Quinovic acid 3-O-.alpha.-L-rhamnopyranoside	632.824	10mM	DMSO
115783-35-2	15,16-Dihydro-15-methoxy-16-oxohardwickiic acid	362.46	10mM	DMSO
No	15-Methoxychinensine A	330.461	10mM	DMSO
486-66-8	Daidzein	254.238	10mM	DMSO
108887-44-1	13-O-Acetylcorianin	336.337	10mM	DMSO
66648-43-9	N-trans-Feruloyltyramine	313.348	10mM	DMSO
54087-32-0	No	274.269	10mM	DMSO
869799-76-8	threo-Guaiacylglycerol beta-coniferyl ether	376.4	10mM	DMSO
7562-61-0	Usnic acid	344.315	10mM	DMSO
132302-25-1	No	1060.95	10mM	DMSO
61665-08-5	11-Methoxyuncarine C	398.452	10mM	DMSO
1021945-29-8	3-O-Methyltirotundin	366.449	10mM	DMSO
4429-63-4	Tabersonine	336.427	10mM	DMSO
1156-78-1	2'-Hydroxygenistein	286.236	10mM	DMSO
145400-03-9	Homalomenol A	238.366	10mM	DMSO
97399-91-2	Aristolactam AIIIa	281.263	10mM	DMSO
13902-62-0	Oplodiol	238.366	10mM	DMSO
No	No	1004.93	10mM	DMSO
256445-68-8	Schleicheol 2	444.733	10mM	DMSO
28449-62-9	Tomentin	222.194	10mM	DMSO
61597-55-5	15,16-Epoxy-12R-hydroxylabda-8(17),13(16),14-triene	302.451	10mM	DMSO
125124-68-7	26-Nor-8-oxo-alpha-onocerin	444.69	10mM	DMSO
38953-85-4	Isovitexin	432.378	10mM	DMSO
56377-67-4	Tirotundin	352.422	10mM	DMSO

27773-39-3	Ervamycine	366.453	10mM	DMSO
182132-59-8	Quinovic acid 3-O-(3',4'-O-isopropylidene)-beta-D-fucopyra	672.888	10mM	DMSO
63807-85-2	Erythrinin C	354.353	10mM	DMSO
90341-45-0	Seneciphyllinine	375.416	10mM	DMSO
60547-63-9	1-Acetyltagitinin A	410.458	10mM	DMSO
66900-93-4	1,2-O-Isopropylidene-beta-D-fructopyranose	341.401	10mM	DMSO
30413-84-4	Corydalmine	256.381	10mM	DMSO
99933-32-1	Bullatantriol	362.417	10mM	DMSO
110382-37-1	3-O-Methyltagitinin F	424.49	10mM	DMSO
No	19(S)-Acetoxy-11-methoxytabersonine	424.702	10mM	DMSO
508-09-8	Glutinone	325.358	10mM	DMSO
86537-66-8	N-Methylcalycinine	538.458	10mM	DMSO
1617-53-4	Amentoflavone	268.35	10mM	DMSO
133453-58-4	Heudelotinone	300.435	10mM	DMSO
162762-93-8	Yunnancoronarin A	474.588	10mM	DMSO
1207181-35-8	Psidial A	472.7	10mM	DMSO
93372-87-3	20-Hydroxy-3-oxo-28-lupanoic acid	352.337	10mM	DMSO
221150-19-2	Erysubin B	320.385	10mM	DMSO
28645-27-4	Meloscandonine	274.398	10mM	DMSO
769140-74-1	13-Methyl-8,11,13-podocarpatriene-3,12-diol	154.163	10mM	DMSO
10597-60-1	2-(3,4-Dihydroxyphenyl)ethanol	440.744	10mM	DMSO
1449-09-8	24-Methylenecycloartan-3-ol	504.699	10mM	DMSO
91095-51-1	3,6,19,23-Tetrahydroxy-12-ursen-28-oic acid	228.2	10mM	DMSO
529-61-3	Euxanthone	252.308	10mM	DMSO
17676-24-3	trans-Hinokiresinol	529.579	10mM	DMSO
1207181-59-6	Scutebata C	484.753	10mM	DMSO
1260-05-5	Phlegmanol C	464.55	10mM	DMSO
659747-28-1	Isogarciniaxanthone E	464.6	10mM	DMSO
14031-37-9	Squalene-2,3-diol	444.733	10mM	DMSO
51276-34-7	2,6-Dimethyl-3,7-octadiene-2,6-diol	170.249	10mM	DMSO
160623-47-2	1,4,6-Trihydroxy-5-methoxy-7-prenylxanthone	342.343	10mM	DMSO
23417-92-7	7-Isopentenyloxy-gamma-fagarine	313.348	10mM	DMSO
162473-22-5	Subelliptenone G	244.2	10mM	DMSO
16503-32-5	Brevilin A	346.417	10mM	DMSO
103744-84-9	Rehmaglutin D	220.65	10mM	DMSO
25368-01-8	Litseglutine B	341.401	10mM	DMSO
14464-90-5	Lyoniresinol	420.453	10mM	DMSO
2169-44-0	Lauroscholtzine	341.401	10mM	DMSO
1449-06-5	21-Episerratenediol	442.717	10mM	DMSO
83324-51-0	8(17),13-Labdadien-15,16-olide	302.451	10mM	DMSO
136055-64-6	No	506.499	10mM	DMSO
1013-69-0	Noreugenin	192.168	10mM	DMSO
130837-92-2	7-Oxohinokinin	368.337	10mM	DMSO
3155-43-9	1,18-Octadecanediol	286.493	10mM	DMSO
216011-55-1	15,16-Epoxy-12S-hydroxylabda-8(17),13(16),14-triene	302.451	10mM	DMSO
2034-74-4	7-Oxo-beta-sitosterol	428.69	10mM	DMSO
156767-69-0	3beta-Hydroxyergost-5-en-7-one	414.664	10mM	DMSO
74161-25-4	2,3-Dehydrokievitone	354.353	10mM	DMSO
41743-56-0	Luteone	354.353	10mM	DMSO
5890-28-8	Cassythicine	325.358	10mM	DMSO
57498-96-1	Carpachromene	336.338	10mM	DMSO
476-70-0	Boldine	327.374	10mM	DMSO
522-11-2	Evoxine	347.362	10mM	DMSO
776325-66-7	1,4,5,6-Tetrahydroxy-7,8-diprenylxanthone	396.433	10mM	DMSO
56218-46-3	Methyl lycernuate A	486.726	10mM	DMSO
50838-11-4	14,15-Didehydroisoeburnamine	294.391	10mM	DMSO
22255-13-6	Guaijaverin	434.35	10mM	DMSO
22149-28-6	11-Hydroxytabersonine	352.427	10mM	DMSO

57576-41-7	Norcepharadione B	307.3	10mM	DMSO
41530-90-9	2,6,16-Kauranetriol	322.482	10mM	DMSO
53948-09-7	Aristolactam BII	279.29	10mM	DMSO
112501-42-5	Aristolactam FI	265.263	10mM	DMSO
112219-48-4	14,15-Didehydrovincamenine	276.375	10mM	DMSO
219861-73-1	2,2',3'-Trihydroxy-4,6-dimethoxybenzophenone	290.268	10mM	DMSO
97456-49-0	Cycloart-22-ene-3,25-diol	442.717	10mM	DMSO
50656-92-3	Vandrikidine	382.453	10mM	DMSO
61135-91-9	3,9-Dihydroxypterocarpan	256.253	10mM	DMSO
36151-01-6	Blumenol B	226.312	10mM	DMSO
22841-42-5	9-Epiblumenol B	226.312	10mM	DMSO
520-32-1	Tricin	330.289	10mM	DMSO
24314-59-8	Scandine	350.411	10mM	DMSO
55604-88-1	1-Oxo-4-hydroxy-2-en-4-ethylcyclohexa-5,8-olide	168.147	10mM	DMSO
672336-50-4	10(14)-Cadinene-4,5-diol	238.366	10mM	DMSO
2141-09-5	Magnoflorine	342.409	10mM	DMSO
23455-44-9	alpha-Spinasterone	410.675	10mM	DMSO
53823-03-3	Onitisin	264.317	10mM	DMSO
150710-72-8	Calyxamine B	195.301	10mM	DMSO
129724-43-2	2',4'-Dihydroxy-3',6'-dimethoxychalcone	300.306	10mM	DMSO
3561-81-7	Mesuaxanthone A	258.226	10mM	DMSO
57566-47-9	Isofuranodiene	216.319	10mM	DMSO
184046-40-0	Dimeric coniferyl acetate	442.458	10mM	DMSO
213769-80-3	6,8-Cyclo-1,4-eudesmanediol	238.366	10mM	DMSO
14028-97-8	N-Methyllindcarpine	327.374	10mM	DMSO
34302-37-9	2,16-Kauranediol	306.483	10mM	DMSO
33973-59-0	Triacetonamine hydrochloride	191.698	10mM	DMSO
No	No	752.779	10mM	DMSO
227471-20-7	Mucrolidin	256.381	10mM	DMSO
119188-37-3	Coronarin D	318.45	10mM	DMSO
20086-07-1	Diosbulbin C	362.374	10mM	DMSO
19533-92-7	Alphitolic acid	472.7	10mM	DMSO
67884-12-2	Martynoside	652.64	10mM	DMSO
833-52-3	7-Hydroxycoumarin-6-carboxylic acid	206.152	10mM	DMSO
53948-10-0	Aristolactam BIII	309.316	10mM	DMSO
4707-47-5	Atraric acid	196.2	10mM	DMSO
1154518-97-4	Sootepin D	484.71	10mM	DMSO
35286-59-0	Ziyuglycoside II	604.814	10mM	DMSO
19309-14-9	Cardamonin	270.28	10mM	DMSO
119533-63-0	Ceanothic acid acetate	528.72	10mM	DMSO
13956-51-9	Lycoclavanol	458.716	10mM	DMSO
214150-74-0	1-Decarboxy-3-oxo-ceanothic acid	440.658	10mM	DMSO
157528-81-9	Coronarin D methyl ether	332.477	10mM	DMSO
111537-41-8	3',5-Dihydroxy-4',5',6,7-tetramethoxyflavone	374.341	10mM	DMSO
2649-68-5	Clovanediol diacetate	322.439	10mM	DMSO
155488-34-9	1,9-Caryolanediol 9-acetate	280.402	10mM	DMSO
145400-02-8	1,4,7-Eudesmanetriol	256.381	10mM	DMSO
5986-49-2	Palustrol	222.366	10mM	DMSO
481-18-5	alpha-Spinasterol	412.691	10mM	DMSO
No	No	500.776	10mM	DMSO
120-80-9	1,2-Benzenediol	110.111	10mM	DMSO
310888-07-4	5,7,3'-Trihydroxy-6,4',5'-trimethoxyflavanone	362.331	10mM	DMSO
260968-11-4	Reneilmol	256.381	10mM	DMSO
183075-03-8	3,4-O-Isopropylidene shikimic acid	214.215	10mM	DMSO
57-87-4	Ergosterol	396.648	10mM	DMSO
No	1,6-Diacetoxy-4(15),11(13)-eudesmadien-12-oic acid methy	364.433	10mM	DMSO
1001424-68-5	1,4,5,6-Tetrahydroxy-7-prenylxanthone	328.316	10mM	DMSO
81053-14-7	4-Hydroxy-4-(methoxycarbonylmethyl)cyclohexanone	186.205	10mM	DMSO

97914-19-7	3,4'-Dihydroxy-3',5,7-trimethoxyflavan	332.348	10mM	DMSO
1911-78-0	Oplopanone	238.366	10mM	DMSO
119188-47-5	10-Hydroxyscandine	366.41	10mM	DMSO
691009-85-5	16-Hydroxy-8(17),13-labdadien-15,16-olid-19-oic acid	348.433	10mM	DMSO
517883-38-4	Rengynic acid	174.194	10mM	DMSO
28757-27-9	Apigenin 5-O-beta-D-glucopyranoside	432.378	10mM	DMSO
490-46-0	Epicatechin	290.268	10mM	DMSO
68097-13-2	4',5,7-Trihydroxy-6-prenylflavone	338.354	10mM	DMSO
53452-32-7	16-Kaurene-2,6,15-triol	320.466	10mM	DMSO
155485-76-0	3,6-Caryolanediol	238.366	10mM	DMSO
2649-64-1	Clovanediol	238.366	10mM	DMSO
4407-36-7	3-Phenyl-2-propen-1-ol	134.175	10mM	DMSO
No	10-Acetoxyscandine	408.447	10mM	DMSO
2955-23-9	Olivil	376.4	10mM	DMSO
486430-93-7	O-Acetylcyclocalopin A	338.352	10mM	DMSO
87-66-1	Pyrogallol	126.11	10mM	DMSO
40768-81-8	1,7-Diepi-8,15-cedranediol	238.366	10mM	DMSO
138965-88-5	Isocoronarin D	318.45	10mM	DMSO
4728-30-7	8(14),15-Isopimaradien-3-ol	288.467	10mM	DMSO
136133-08-9	Ellagic acid 7-O-beta-D-xylopyranoside-2,3,8-trimethyl ethe	476.387	10mM	DMSO
486430-94-8	Cyclocalopin A	296.316	10mM	DMSO
30828-09-2	4-Oxododecanedioic acid	244.284	10mM	DMSO
88642-46-0	Cerebroside B	728.051	10mM	DMSO
66-22-8	Uracil	112.087	10mM	DMSO
6890-88-6	Eburicol	440.744	10mM	DMSO
138965-89-6	Coronarin D ethyl ether	346.504	10mM	DMSO
No	Isocoronarin D methylthiomethyl ether	378.569	10mM	DMSO
143120-46-1	Neotuberostemonine	375.502	10mM	DMSO
173294-74-1	Garciniaxanthone E	464.55	10mM	DMSO
484-20-8	Bergapten	216.19	10mM	DMSO
No	No	323.427	10mM	DMSO
58-61-7	Adenosine	267.241	10mM	DMSO
446-72-0	Genistein	270.237	10mM	DMSO
90-24-4	Xanthoxylin	196.2	10mM	DMSO
20086-06-0	Diosbulbin B	344.358	10mM	DMSO
474893-07-7	2,16-Kauranediol 2-O-beta-D-allopyranoside	468.623	10mM	DMSO
523-50-2	Angelicin	186.164	10mM	DMSO
67-47-0	5-Hydroxymethylfurfural	126.11	10mM	DMSO
144424-80-6	3-O-(E)-p-Coumaroylbetulin	588.86	10mM	DMSO
10309-37-2	Bakuchiol	256.383	10mM	DMSO
83725-24-0	Pomolic acid 28-O-beta-D-glucopyranosyl ester	634.84	10mM	DMSO
943136-39-8	Chlorahololide D	676.749	10mM	DMSO
103630-03-1	Catechin 3-rhamnoside	436.409	10mM	DMSO
60048-88-6	N-Demethylechitamine	370.442	10mM	DMSO
66-97-7	Psoralen	186.164	10mM	DMSO
769928-72-5	15-Methoxypinusolidic acid	362.46	10mM	DMSO
58762-96-2	Pinostilbenoside	404.41	10mM	DMSO
958885-86-4	12-Hydroxy-8(17),13-labdadien-16,15-olide	318.45	10mM	DMSO
160047-56-3	Sambutoxin	453.614	10mM	DMSO
106861-40-9	Bisdehydrotuberostemonine	371.47	10mM	DMSO
521-61-9	Physcion	284.263	10mM	DMSO
826-36-8	Triacetonamine	155.237	10mM	DMSO
477953-07-4	Neostenine	277.402	10mM	DMSO
528-43-8	Magnolol	266.334	10mM	DMSO
153-18-4	Rutin	610.518	10mM	DMSO
2457-80-9	5'-S-Methyl-5'-thioadenosine	297.334	10mM	DMSO
112237-71-5	16-O-Methyl-14,15-didehydroisovincanol	308.417	10mM	DMSO
22255-10-3	alpha-Amyrin palmitate	665.126	10mM	DMSO

53755-76-3	Lyclaninol	474.716	10mM	DMSO
485-19-8	Reticuline	329.39	10mM	DMSO
19879-30-2	Bavachinin	338.397	10mM	DMSO
2086-83-1	Berberine	336.361	10mM	DMSO
81264-00-8	ent-6,9-Dihydroxy-15-oxo-16-kauren-19-oic acid	348.433	10mM	DMSO
24513-51-7	3,21-Dihydroxy-14-serraten-16-one	456.7	10mM	DMSO
27740-43-8	Erysotrine	313.391	10mM	DMSO
83011-43-2	Methyl 3-hydroxy-4,5-dimethoxybenzoate	212.199	10mM	DMSO
24352-51-0	3,5-Cycloergosta-6,8(14),22-triene	378.633	10mM	DMSO
221150-18-1	Erysubin A	352.337	10mM	DMSO
1014974-98-1	No	658.602	10mM	DMSO
642-71-7	Antiarol	184.189	10mM	DMSO
20784-60-5	4'-O-Methylbroussochalcone B	338.397	10mM	DMSO
53947-92-5	Corylin	320.339	10mM	DMSO
929637-35-4	Sessilifoline A	389.485	10mM	DMSO
573-44-4	Liriodendrin	742.718	10mM	DMSO
220880-90-0	2,3-O-Isopropylidenyl euscaphic acid	528.763	10mM	DMSO
1190225-47-8	Sarcandrone A	554.587	10mM	DMSO
14351-29-2	Dammarenediol II	444.733	10mM	DMSO
57586-98-8	Cycloartane-3,24,25-triol	460.732	10mM	DMSO
66107-60-6	14,17-Epidioxy-28-nor-15-taraxerene-2,3-diol	458.673	10mM	DMSO
24513-57-3	21-Episerratriol	458.716	10mM	DMSO
31524-62-6	Isobavachin	324.37	10mM	DMSO
19879-32-4	Bavachin	324.37	10mM	DMSO
13956-52-0	Serratriol	458.716	10mM	DMSO
1180-71-8	Limonin	470.512	10mM	DMSO
863-76-3	alpha-Amyrin acetate	468.754	10mM	DMSO
59219-64-6	8(14),15-Isopimaradiene-3,18-diol	304.467	10mM	DMSO
1187951-05-8	Diosbulbin I	506.544	10mM	DMSO
19942-04-2	3-Epicabraleadiol	460.732	10mM	DMSO
66756-57-8	Diosbulbin D	344.358	10mM	DMSO
6009-12-7	Confluentic acid	500.581	10mM	DMSO
126882-53-9	Ssioriside	554.583	10mM	DMSO
94410-22-7	Isomartynoside	652.64	10mM	DMSO
219649-95-3	Griffipavixanthone	652.6	10mM	DMSO
595-15-3	Soyasapogenol B	458.716	10mM	DMSO
149-91-7	Gallic acid	170.12	10mM	DMSO
55-21-0	Benzamide	121.137	10mM	DMSO
504-15-4	Orcinol	124.137	10mM	DMSO
751-03-1	Obacunone	454.512	10mM	DMSO
261351-23-9	Antiarol rutinoside	492.471	10mM	DMSO
35286-58-9	Ziyuglycoside I	766.955	10mM	DMSO
87562-76-3	Kelampayoside A	478.444	10mM	DMSO
No	3-Epiwightianol A tetraacetate	660.878	10mM	DMSO
478-01-3	Nobiletin	402.395	10mM	DMSO
23518-30-1	Licarin A	326.386	10mM	DMSO
465-00-9	Arjunolic acid	488.699	10mM	DMSO
144868-43-9	Garjasmin	224.21	10mM	DMSO
139682-36-3	Gardenine	223.225	10mM	DMSO
No	Diosbulbin C ethyl ester	390.427	10mM	DMSO
114916-05-1	N1,N10-Bis(p-coumaroyl)spermidine	437.531	10mM	DMSO
53452-34-9	Creticoside C	484.623	10mM	DMSO
60-35-5	Acetamide	59.0672	10mM	DMSO
3368-87-4	19,20-(E)-Vallesamine	340.416	10mM	DMSO
1466-76-8	2,6-Dimethoxybenzoic acid	182.173	10mM	DMSO
37239-47-7	Wilforgine	857.807	10mM	DMSO
11088-09-8	Wilforine	867.845	10mM	DMSO
1194-98-5	2,5-Dihydroxybenzaldehyde	138.121	10mM	DMSO

67567-15-1	Diosbulbin G	346.374	10mM	DMSO
83-74-9	Ibogaine	310.433	10mM	DMSO
24512-63-8	Geniposide	388.366	10mM	DMSO
525-21-3	Fraxidin	222.194	10mM	DMSO
115753-79-2	Galanolactone	318.45	10mM	DMSO
28619-41-2	Erythristemine	343.417	10mM	DMSO
1033747-78-2	Itol A	368.464	10mM	DMSO
134-96-3	4-Hydroxy-3,5-dimethoxybenzaldehyde	182.173	10mM	DMSO
22570-53-2	Zeorin	444.733	10mM	DMSO
10283-68-8	Dihydrocinchonamine	298.423	10mM	DMSO
564-73-8	Hinokiol	302.451	10mM	DMSO
No	Nyasicol 1,2-acetonide	356.369	10mM	DMSO
111518-95-7	Nyasicol	316.305	10mM	DMSO
142937-50-6	Triptoquinone B	330.418	10mM	DMSO
142741-24-0	Conophylline	794.889	10mM	DMSO
144735-57-9	2-(4-Hydroxy-3-methoxyphenyl)-7-methoxy-5-benzofuranpr	328.359	10mM	DMSO
195735-16-1	2,6,16-Kauranetriol 2-O-beta-D-allopyranoside	484.623	10mM	DMSO
491-54-3	Kaempferide	300.263	10mM	DMSO
56973-66-1	9,9'-Di-O-(E)-feruloylsecoisolariciresinol	714.754	10mM	DMSO
22031-64-7	Cinnamamide	147.174	10mM	DMSO
52358-58-4	Erysotramidine	327.374	10mM	DMSO
166322-14-1	9-O-Feruloyl-5,5'-dimethoxylariciresinol	596.622	10mM	DMSO
1187951-06-9	Diosbulbin J	378.373	10mM	DMSO
No	Glochionionol A	240.296	10mM	DMSO
1136932-34-7	Shizukanolide H	304.338	10mM	DMSO
828935-47-3	3-Oxo-24,25,26,27-tetranortirucall-7-en-23,21-olide	398.578	10mM	DMSO
163513-81-3	Triptoquinonide	326.386	10mM	DMSO
No	14-O-methylthiomethylitol A	428.583	10mM	DMSO
51666-26-3	Erythrartine	329.39	10mM	DMSO
20869-95-8	Ermanin	314.289	10mM	DMSO
131-11-3	Dimethyl phthalate	194.184	10mM	DMSO
464-92-6	Asiatic acid	488.699	10mM	DMSO
83133-17-9	4',5-Dihydroxy-3',5',6,7-tetramethoxyflavone	374.341	10mM	DMSO
625-04-7	Diacetonamine	115.174	10mM	DMSO
57-50-1	Sucrose	342.296	10mM	DMSO
31685-80-0	Pinusolide	346.461	10mM	DMSO
2033-89-8	3,4-Dimethoxyphenol	154.163	10mM	DMSO
41653-73-0	Koaburaside	332.303	10mM	DMSO
53755-77-4	Lycernuic acid A	472.7	10mM	DMSO
No	6-Ethoxygeniposide	432.419	10mM	DMSO
15486-33-6	3,5-Dihydroxy-4',7-dimethoxyflavone	314.289	10mM	DMSO
62470-46-6	p-Vinylphenyl O-beta-D-glucopyranoside	282.289	10mM	DMSO
426821-85-4	4-Hydroxy-3-methoxyphenyl O-beta-D-(6'-O-syringate)gluc	482.435	10mM	DMSO
73584-67-5	27-p-Coumaroyloxyursolic acid	618.842	10mM	DMSO
No	18-O-Demethyldiosbulbin F	362.374	10mM	DMSO
65597-42-4	Cerbinal	204.179	10mM	DMSO
548-29-8	Isolariciresinol	360.401	10mM	DMSO
No	8-Hydroxy-17-chloro-12-labden-16,15-olide	354.911	10mM	DMSO
26652-12-0	Salirepin	302.277	10mM	DMSO
136172-60-6	6-O-Caffeoylarbutin	434.393	10mM	DMSO
58822-47-2	Secoxyloganin	404.366	10mM	DMSO
23132-13-0	Heveaflavone	580.538	10mM	DMSO
111518-94-6	Nyasicoside	478.446	10mM	DMSO
66547-92-0	Dehydroheliobuphthalmin	412.389	10mM	DMSO
64421-27-8	Mussaenoside	390.382	10mM	DMSO
954379-68-1	Neotuberostemonone	405.485	10mM	DMSO
18463-25-7	Nigracin	406.383	10mM	DMSO
73002-86-5	15-Isopimarene-8,18-diol	306.483	10mM	DMSO

3187-58-4	Methyl orsellinate	182.173	10mM	DMSO
27530-67-2	Feretoside	404.366	10mM	DMSO
54377-24-1	7-Hydroxy-5,8-dimethoxyflavanone	300.306	10mM	DMSO
3650-31-5	Agatholal	304.467	10mM	DMSO
5128-44-9	5-Hydroxy-4',7-dimethoxyflavone	298.29	10mM	DMSO
69768-97-4	4-(3,4-Dimethoxyphenyl)-3-buten-1-ol	208.254	10mM	DMSO
145544-91-8	Eucamalol	168.233	10mM	DMSO
195723-38-7	2,16,19-Kauranetriol 2-O-beta-D-allopyranoside	484.623	10mM	DMSO
95416-25-4	15-Hydroxy-7-oxo-8,11,13-abietatrien-18-oic acid	330.418	10mM	DMSO
1038922-95-0	Mannioside A	738.902	10mM	DMSO
1107620-67-6	Garcinexanthone A	342.343	10mM	DMSO
149155-19-1	Homaloside D	544.504	10mM	DMSO
173429-83-9	Ficusin A	404.455	10mM	DMSO
81263-96-9	ent-9-Hydroxy-15-oxo-16-kauren-19-oic acid beta-D-glucop	494.575	10mM	DMSO
23407-76-3	Theviridoside	404.366	10mM	DMSO
469-39-6	Cycloeucalenol	426.717	10mM	DMSO
942480-13-9	Nemoralisin	332.434	10mM	DMSO
36150-04-6	Mullilam diol	188.264	10mM	DMSO
142279-42-3	Shizukaol D	578.649	10mM	DMSO
29080-58-8	5-Hydroxy-3',4',7-trimethoxyflavone	328.316	10mM	DMSO
50906-56-4	Arteannuin B	248.318	10mM	DMSO
74560-05-7	Isomedicarpin	270.28	10mM	DMSO
100198-09-2	Piscidinol A	474.716	10mM	DMSO
117-81-7	Bis(2-ethylhexyl) phthalate	390.556	10mM	DMSO
103917-26-6	Lupeol caffeate	588.86	10mM	DMSO
943989-68-2	Iriflophenone 2-O-alpha-L-rhamnopyranoside	392.357	10mM	DMSO
244204-40-8	Celaphanol A	288.338	10mM	DMSO
34444-37-6	Nortrachelogenin	374.384	10mM	DMSO
76-78-8	Quassin	388.454	10mM	DMSO
60129-64-8	Paniculoside II	496.59	10mM	DMSO
77658-45-8	ent-9-Hydroxy-15-oxo-19-kauranoic acid	334.45	10mM	DMSO
77658-39-0	ent-9-Hydroxy-15-oxo-16-kauren-19-oic acid	332.434	10mM	DMSO
68436-47-5	Isowighteone	338.354	10mM	DMSO
545-46-0	Uvaol	442.717	10mM	DMSO
93-07-2	3,4-Dimethoxybenzoic acid	182.173	10mM	DMSO
115334-04-8	Phellochin	488.742	10mM	DMSO
56324-54-0	Hedychenone	298.419	10mM	DMSO
144881-21-0	4',9,9'-Trihydroxy-3'-methoxy-3,7'-epoxy-4,8'-oxyneolignan	346.374	10mM	DMSO
100234-59-1	Picrasidine I	240.257	10mM	DMSO
20316-62-5	Tiliroside	594.52	10mM	DMSO
No	No	414.448	10mM	DMSO
No	No	442.458	10mM	DMSO
57719-76-3	11,15-Dihydroxy-16-kauren-19-oic acid	334.45	10mM	DMSO
226562-47-6	3-Hydroxy-12-oleanene-23,28-dioic acid	486.683	10mM	DMSO
23963-54-4	Glochidonol	440.701	10mM	DMSO
89130-86-9	Betulin caffeate	604.859	10mM	DMSO
72826-63-2	Deoxyartemisinin	266.333	10mM	DMSO
126882-76-6	3-Methoxy-5-heneicosylphenol	418.695	10mM	DMSO
39024-15-2	3-Epiwilsonine	343.417	10mM	DMSO
39024-12-9	Wilsonine	343.417	10mM	DMSO
80286-58-4	Artemisinic acid	234.334	10mM	DMSO
52591-10-3	Iriflophenone	246.215	10mM	DMSO
466-01-3	Hederagonic acid	470.684	10mM	DMSO
16962-90-6	Odoratone	472.7	10mM	DMSO
537-98-4	Ferulic acid	194.184	10mM	DMSO
33228-65-8	Glucosyringic acid	360.313	10mM	DMSO
20175-84-2	Isodiospyrin	374.343	10mM	DMSO
40716-66-3	Nerolidol	222.366	10mM	DMSO

140447-22-9	Ergosterol peroxide 3-O-beta-D-glucopyranoside	590.788	10mM	DMSO
51804-68-3	Oxoepistephamiersine	403.426	10mM	DMSO
51804-69-4	Dihydrooxoepistephamiersine	405.442	10mM	DMSO
209115-67-3	Mangochinine	328.382	10mM	DMSO
6985-35-9	Bourjotinolone A	472.7	10mM	DMSO
No	23-Nor-3-oxo-12-oleanen-28-oic acid	440.658	10mM	DMSO
578-74-5	Cosmosiin	432.378	10mM	DMSO
25739-41-7	Velutin	314.289	10mM	DMSO
73891-72-2	Hispidone	472.7	10mM	DMSO
30315-04-9	Picrasin B acetate	418.48	10mM	DMSO
97372-53-7	8-Hydroxy-4-cadinen-3-one	236.35	10mM	DMSO
58-95-7	alpha-Tocopherol acetate	472.743	10mM	DMSO
79491-71-7	9-Oxo-10,11-dehydroageraphorone	232.318	10mM	DMSO
74713-15-8	Secoxyloganin methyl ester	418.392	10mM	DMSO
337527-10-3	8,11,13-Abietatriene-7,15,18-triol	318.45	10mM	DMSO
24274-60-0	Acetylcephalotaxine	357.4	10mM	DMSO
No	Simiarenol methylthiomethyl ether	486.836	10mM	DMSO
29028-10-2	3-Epiglochidiol	442.717	10mM	DMSO
51095-85-3	2,7-Dihydrohomoerysotrine	329.433	10mM	DMSO
139726-29-7	Dunnianol	398.494	10mM	DMSO
104975-02-2	3-Acetoxy-4,7(11)-cadinadien-8-one	276.371	10mM	DMSO
33900-74-2	2,3,4'-Trihydroxy-3',5'-dimethoxypropiophenone	242.225	10mM	DMSO
55481-88-4	Mollugin	284.307	10mM	DMSO
61658-41-1	Furomollugin	242.227	10mM	DMSO
15291-75-5	Ginkgolide A	408.399	10mM	DMSO
15291-77-7	Ginkgolide B	424.399	10mM	DMSO
15291-76-6	Ginkgolide C	440.398	10mM	DMSO
6807-83-6	Trifolirhizin	446.404	10mM	DMSO
519-02-8	Matrine	248.364	10mM	DMSO
16837-52-8	Oxymatrine	264.363	10mM	DMSO
3621-38-3	Jatrorrhizine	338.377	10mM	DMSO
3486-67-7	Palmatine	352.404	10mM	DMSO
481-72-1	Aloeemodin	270.237	10mM	DMSO
2447-54-3	Sanguinarine	332.329	10mM	DMSO
7689-03-4	Camptothecin	348.352	10mM	DMSO
58316-41-9	Saikosaponin B2	780.982	10mM	DMSO
29883-15-6	Amygdalin	457.428	10mM	DMSO
50773-41-6	Chonglou Saponin I	855.017	10mM	DMSO
68124-04-9	Chonglou Saponin VII	1031.18	10mM	DMSO
21082-33-7	Sakakin	286.278	10mM	DMSO
19254-69-4	Ergosta-4,6,8(14),22-tetraen-3-one	392.617	10mM	DMSO
22144-77-0	Cytochalasin D	507.618	10mM	DMSO
477-90-7	Bergenin	328.271	10mM	DMSO
14531-47-6	Penta-acetate bergenin	538.455	10mM	DMSO
33815-57-5	Di-O-methylbergenin	356.325	10mM	DMSO
No	11-O-(3-nitrobenzoyl)bergenin	477.375	10mM	DMSO
85643-19-2	Curculigoside	466.435	10mM	DMSO
146905-24-0	1,2-Diacetoxy-4,7,8-trihydroxy-3-(4-hydroxyphenyl)dibenzo	424.357	10mM	DMSO
479-20-9	Atranorin	374.341	10mM	DMSO
63968-64-9	Artemisinin	282.332	10mM	DMSO
569-83-5	Xanthohumol	354.396	10mM	DMSO
274675-25-1	Xanthohumol D	370.396	10mM	DMSO
647853-82-5	No	478.446	10mM	DMSO
60-81-1	Phloridzin	436.409	10mM	DMSO
4192-90-9	p-Phlorizin	436.409	10mM	DMSO
80557-12-6	Grifolic acid	372.498	10mM	DMSO
329975-47-5	3,4-Secocucurbita-4,24-diene-3,26,29-trioic acid	502.683	10mM	DMSO
50-89-5	Thymidine	242.229	10mM	DMSO

38395-02-7	Caudatin	490.629	10mM	DMSO
84745-94-8	Qingyanshengenin	500.581	10mM	DMSO
152175-76-3	Forrestin A	578.648	10mM	DMSO
269742-39-4	16-Acetoxy-7-O-acetylhorminone	432.507	10mM	DMSO
120462-42-2	Lophanthoidin B	448.506	10mM	DMSO
120462-45-5	Lophanthoidin E	406.469	10mM	DMSO
120462-46-6	Lophanthoidin F	434.523	10mM	DMSO
125164-55-8	Rosthornin A	376.486	10mM	DMSO
125181-21-7	Rosthornin B	434.523	10mM	DMSO
64657-21-2	Coleonol B	410.501	10mM	DMSO
473981-11-2	Forskolin G	436.538	10mM	DMSO
81873-08-7	Forskolin J	452.538	10mM	DMSO
123043-54-9	Bulleyanin	534.595	10mM	DMSO
122717-54-8	Liangshanin A	330.418	10mM	DMSO
78536-36-4	Excisanin B	392.486	10mM	DMSO
39388-57-3	Kamebanin	334.45	10mM	DMSO
85329-59-5	Trichorabdal A	346.417	10mM	DMSO
959860-49-2	Guajadial	474.588	10mM	DMSO
491-67-8	Baicalein	270.237	10mM	DMSO
5508-58-7	Andrographolide	350.449	10mM	DMSO
28955-30-8	Cassiachromone	232.232	10mM	DMSO
No	Dihydrocassiachromone	234.248	10mM	DMSO
168254-95-3	Wilforol C	472.7	10mM	DMSO
116498-58-9	5,5'-Dimethoxylariciresinol	420.453	10mM	DMSO
2318-78-7	Simiarenone	424.702	10mM	DMSO
4965-99-5	Simiarenol acetate	468.754	10mM	DMSO
20831-76-9	Gentiopicroside	356.325	10mM	DMSO
890928-81-1	24,25-Epoxytirucall-7-en-3,23-dione	454.684	10mM	DMSO
No	Simplidin butyl ether	446.49	10mM	DMSO
458-37-7	Curcumin	368.38	10mM	DMSO
29388-59-8	Secoisolariciresinol	362.417	10mM	DMSO
305364-91-4	5'-Demethylaquillochin	402.352	10mM	DMSO
421583-14-4	Cephalocyclidin A	317.336	10mM	DMSO
57361-74-7	Dihydroepistephamiersine 6-acetate	433.495	10mM	DMSO
596799-30-3	Calyciphylline A	385.497	10mM	DMSO
7044-31-7	17alpha-Neriifolin	534.681	10mM	DMSO
52389-15-8	Epistephamiersine	389.442	10mM	DMSO
139122-81-9	Tripterifordin	318.45	10mM	DMSO
3772-56-3	Totaradiol	302.451	10mM	DMSO
63807-90-9	Dihydroalpinumisoflavone	338.354	10mM	DMSO
60761-00-4	2-Hydroxy-1,8-cineole	170.249	10mM	DMSO
55511-08-5	Biondinin C	300.392	10mM	DMSO
155709-41-4	Isomagnolone	282.334	10mM	DMSO
139726-30-0	Isodunnianol	398.494	10mM	DMSO
65388-03-6	alpha-Isowighteone	338.354	10mM	DMSO
28199-69-1	Dihydrodehydrodiconiferyl alcohol	360.401	10mM	DMSO
114586-47-9	Thevebioside	696.822	10mM	DMSO
21401-21-8	Taxiphyllin	311.287	10mM	DMSO
529-44-2	Myricetin	318.235	10mM	DMSO
16844-71-6	Epifriedelanol	428.733	10mM	DMSO
247036-52-8	6-Deoxy-9alpha-hydroxycedrodorin	502.553	10mM	DMSO
123621-00-1	Clerosterol glucoside	574.831	10mM	DMSO
262355-96-4	7,15-Dihydroxy-8(14)-podocarpen-13-one	278.387	10mM	DMSO
115074-93-6	Soyacerebroside II	714.025	10mM	DMSO
2761-77-5	Communic acid	302.451	10mM	DMSO
164661-12-5	4-(3,4-Dimethoxyphenyl)-3-butene-1,2-diol	224.253	10mM	DMSO
74683-19-5	3-Methoxy-4,5-methylenedioxycinnamaldehyde	206.195	10mM	DMSO
70389-88-7	Voleneol	238.366	10mM	DMSO

14694-15-6	Codaphniphylline	469.699	10mM	DMSO
6610-55-5	Glochidone	422.686	10mM	DMSO
52914-31-5	Dammaradienyl acetate	468.754	10mM	DMSO
84567-08-8	4-Hydroxycephalotaxine	331.363	10mM	DMSO
545-48-2	Erythrodiol	442.717	10mM	DMSO
1202-41-1	3,4-Dihydroxycinnamamide	179.173	10mM	DMSO
140447-14-9	11-Hydroxyjasmonic acid	226.269	10mM	DMSO
217810-46-3	9,16-Dioxo-10,12,14-octadecatrienoic acid	306.397	10mM	DMSO
1745-36-4	alpha-Spinasterol glucoside	574.831	10mM	DMSO
4651-46-1	alpha-Spinasterol acetate	454.728	10mM	DMSO
84104-71-2	Wilforlide A	454.684	10mM	DMSO
114613-59-1	17alpha-Thevebioside	696.822	10mM	DMSO
1092555-03-7	Paxiphylline E	399.48	10mM	DMSO
155051-85-7	4-Hydroxy-3-(3-methyl-2-butenoyl)-5-(3-methyl-2-butenyl)l	288.338	10mM	DMSO
81263-97-0	ent-6,11-Dihydroxy-15-oxo-16-kauren-19-oic acid beta-D-g	510.574	10mM	DMSO
65894-41-9	Daturabietatriene	302.451	10mM	DMSO
203455-81-6	18-nor-8,11,13-Abietatriene-4,15-diol	288.424	10mM	DMSO
127-27-5	Pimaric acid	302.451	10mM	DMSO
112244-29-8	Stigmastane-3,6-diol	432.722	10mM	DMSO
1259-94-5	24-Methylenecycloartanol acetate	482.781	10mM	DMSO
39729-21-0	Daphmacropodine	513.752	10mM	DMSO
97-59-6	Allantoin	158.115	10mM	DMSO
126594-64-7	Cerberidol	172.222	10mM	DMSO
380487-65-0	alpha-Epoxydihydroartemisinic acid	252.349	10mM	DMSO
59014-02-7	8-Hydroxyhyperforin 8,1-hemiacetal	552.784	10mM	DMSO
6246-46-4	Ursonic acid	454.684	10mM	DMSO
81263-98-1	ent-6,9-Dihydroxy-15-oxo-16-kauren-19-oic acid beta-D-glu	510.574	10mM	DMSO
37687-34-6	6-Acetonyldihydrosanguinarine	389.401	10mM	DMSO
13403-14-0	Methyl beta-D-fructofuranoside	194.182	10mM	DMSO
1820-84-4	Ethyl beta-D-fructofuranoside	208.209	10mM	DMSO
126594-66-9	Cyclocerberidol	188.221	10mM	DMSO
84108-17-8	Triptotriterpenic acid A	472.7	10mM	DMSO
217650-27-6	4-Cadinen-7-ol	222.366	10mM	DMSO
207446-92-2	Arteannuin N	250.333	10mM	DMSO
207446-89-7	Arteannuin L	250.333	10mM	DMSO
81371-54-2	Momordicoside G	632.867	10mM	DMSO
30273-62-2	3,4,5-Trimethoxycinnamyl alcohol	224.253	10mM	DMSO
640289-58-3	4(15)-Oppositene-1,7-diol	238.366	10mM	DMSO
38968-07-9	2'-O-Methylperlatolic acid	458.544	10mM	DMSO
22864-92-2	6-Acetonyldihydrochelerythrine	405.443	10mM	DMSO
66648-50-8	Ethyl caffeate	208.211	10mM	DMSO
60820-94-2	Phytolaccoside B	664.823	10mM	DMSO
104669-02-5	Iriflophenone 3-C-beta-D-glucopyranoside	408.356	10mM	DMSO
249916-07-2	Borreriagenin	214.215	10mM	DMSO
480-19-3	Isorhamnetin	316.262	10mM	DMSO
57576-34-8	(2,4-Dihydroxyphenyl)acetonitrile	149.147	10mM	DMSO
923950-05-4	3-Acetoxy-4-cadinen-8-one	278.387	10mM	DMSO
6872-88-4	Xanthoplanine	356.435	10mM	DMSO
7224-61-5	Laurifoline	342.409	10mM	DMSO
881388-88-1	Daphniyunnine B	343.46	10mM	DMSO
197018-71-6	5-Hydroxy-4',7-dimethoxyflavone 5-O-beta-D-glucopyranos	460.431	10mM	DMSO
28420-25-9	6-Hydroxy-2,6-dimethyl-2,7-octadienoic acid	184.232	10mM	DMSO
280565-85-7	Rediocide A	794.924	10mM	DMSO
81910-41-0	Momordicoside I aglycone	456.7	10mM	DMSO
213329-45-4	15,18-Dihydroxy-8,11,13-abietatrien-7-one	316.435	10mM	DMSO
85372-72-1	5,19-Epoxy-19,25-dimethoxycucurbita-6,23-dien-3-ol	500.753	10mM	DMSO
87095-74-7	5-Hydroxy-1,7-diphenyl-6-hepten-3-one	280.361	10mM	DMSO
912329-03-4	Karavilagenin A	486.769	10mM	DMSO

677277-98-4	Rediocide C	814.913	10mM	DMSO
1210347-50-4	Abiesadine I	402.524	10mM	DMSO
491-71-4	Chrysoeriol	300.263	10mM	DMSO
143519-04-4	3-Acetoxy-24-hydroxydammara-20,25-diene	484.753	10mM	DMSO
21040-45-9	Cinnamyl acetate	176.212	10mM	DMSO
127350-68-9	Coclauril	151.163	10mM	DMSO
2688-49-5	6-Hydroxybenzofuran-2(3H)-one	150.131	10mM	DMSO
No	alpha-Spinasterol methylthiomethyl ether	472.809	10mM	DMSO
155709-40-3	Simonsinol	398.494	10mM	DMSO
92466-31-4	4,5-Epoxyartemisinic acid	250.333	10mM	DMSO
24240-04-8	Allocryptopine	369.411	10mM	DMSO
33116-33-5	Stephavanine	497.494	10mM	DMSO
194940-15-3	4-Hydroxycinnamamide	163.173	10mM	DMSO
486-39-5	Coclaurine	285.338	10mM	DMSO
619-57-8	4-Hydroxybenzamide	137.136	10mM	DMSO
221289-20-9	Lethedoside A	490.457	10mM	DMSO
14260-99-2	Daphylloside	446.403	10mM	DMSO
109194-60-7	Tachioside	302.277	10mM	DMSO
58738-31-1	16-Oxoprometaphanine	373.4	10mM	DMSO
155205-65-5	7,15-Dihydroxydehydroabietic acid methyl ester	346.461	10mM	DMSO
40672-47-7	Taxifolin 3-O-beta-D-xylopyranoside	436.366	10mM	DMSO
14371-10-9	Cinnamaldehyde	132.159	10mM	DMSO
614-82-4	2,4-Dihydroxyphenylacetic acid	168.147	10mM	DMSO
130-86-9	Protopine	353.369	10mM	DMSO
213329-46-5	4,15-Dihydroxy-18-nor-8,11,13-abietatrien-7-one	302.408	10mM	DMSO
4481-62-3	Betulonic acid	454.684	10mM	DMSO
No	7-Geranyloxy-5-methoxycoumarin	328.402	10mM	DMSO
1228175-65-2	8-Geranyloxy-5,7-dimethoxycoumarin	358.428	10mM	DMSO
827319-50-6	Perforatumone	552.784	10mM	DMSO
189109-45-3	Byzantionoside B	372.453	10mM	DMSO
1138156-77-0	N-p-coumaroyl-N'-caffeoylputrescine	396.436	10mM	DMSO
21499-24-1	Agrimonolide	314.333	10mM	DMSO
132951-90-7	Macrocarpal A	472.614	10mM	DMSO
105181-06-4	9-Oxoageraphorone	234.334	10mM	DMSO
62356-47-2	Isotschimgin	274.355	10mM	DMSO
1042143-83-8	Yunnandaphninine G	469.699	10mM	DMSO
82442-48-6	Arteannuin A	206.281	10mM	DMSO
64929-59-5	Sendanolactone	466.652	10mM	DMSO
6858-85-1	Prometaphanine	359.416	10mM	DMSO
96552-59-9	5,7-Dihydroxy-2-isopropylchromone	220.221	10mM	DMSO
162602-04-2	3-Hydroxy-5,7-dimethoxy-3',4'-methylenedioxyflavan	330.332	10mM	DMSO
10083-24-6	Piceatannol	244.243	10mM	DMSO
No	No	510.575	10mM	DMSO
No	No	482.565	10mM	DMSO
509077-91-2	Taiwanhomoflavone B	568.527	10mM	DMSO
17622-86-5	Nervogenic acid	274.355	10mM	DMSO
26121-56-2	Picrasin B	376.443	10mM	DMSO
104777-61-9	3alpha-Akebonoic acid	440.658	10mM	DMSO
246266-38-6	3-Geranyl-4-methoxybenzoic acid	288.381	10mM	DMSO
No	No			
151731-50-9	2,2-Dimethyl-8-prenylchromene 6-carboxylic acid	272.339	10mM	DMSO
19775-48-5	Daphmacrine	511.736	10mM	DMSO
619326-75-9	Deoxyisocalyciphylline B	341.487	10mM	DMSO
341971-45-7	Actinidic acid	486.683	10mM	DMSO
149-32-6	Erythritol	122.12	10mM	DMSO
95732-59-5	Hedyotisol A	810.837	10mM	DMSO
60008-01-7	Oblongine	314.398	10mM	DMSO
104055-79-0	No	324.37	10mM	DMSO

206757-32-6	Pedatisectine F	214.218	10mM	DMSO
6147-11-1	alpha-Mangostin	410.46	10mM	DMSO
315236-68-1	Sutherlandin trans-p-coumarate	421.398	10mM	DMSO
501-94-0	2-(4-Hydroxyphenyl)ethanol	138.164	10mM	DMSO
188300-19-8	Massonianoside B	492.516	10mM	DMSO
222629-77-8	Antidesmone	319.438	10mM	DMSO
81678-46-8	3-Dehydro-15-deoxoeucosterol	456.657	10mM	DMSO
53538-13-9	Apigenin 7-O-methylglucuronide	460.388	10mM	DMSO
101140-06-1	3,8"-Biapigenin	538.458	10mM	DMSO
75775-36-9	Cedrusin	346.374	10mM	DMSO
128397-41-1	Hydroprotopine	354.376	10mM	DMSO
31271-07-5	gamma-Mangostin	396.433	10mM	DMSO
81241-53-4	15-Deoxoeucosterol	458.673	10mM	DMSO
26063-95-6	1-Isomangostin hydrate	428.475	10mM	DMSO
76689-98-0	1-Deacetylnimbolinin B	584.697	10mM	DMSO
33390-42-0	Gartanin	396.433	10mM	DMSO
62498-83-3	3-O-Acetyloleanderolide	514.736	10mM	DMSO
27391-16-8	threo-Guaiacylglycerol	214.215	10mM	DMSO
247078-43-9	Daphnezomine B	375.545	10mM	DMSO
57420-46-9	8-O-Acetylshanzhiside methyl ester	448.418	10mM	DMSO
26063-96-7	3-Isomangostin hydrate	428.475	10mM	DMSO
61775-19-7	5-O-Methylnaringenin	286.279	10mM	DMSO
72396-01-1	Yuheinoside	360.356	10mM	DMSO
34366-34-2	Isoapetalic acid	388.454	10mM	DMSO
925705-36-8	3-Isomangostin hydrate formate	456.485	10mM	DMSO
5273-86-9	beta-Asarone	208.254	10mM	DMSO
21698-44-2	Shyobunone	220.35	10mM	DMSO
52706-07-7	Scillascillin	312.274	10mM	DMSO
52096-50-1	2-Hydroxy-7-O-methylscillascillin	342.3	10mM	DMSO
30220-43-0	Effusanin A	348.433	10mM	DMSO
1126-61-0	4-Allylpyrocatechol	150.174	10mM	DMSO
119725-20-1	Fupenzic acid	484.667	10mM	DMSO
217466-37-0	1-Hydroxy-2-oxopomolic acid	502.683	10mM	DMSO
259653-54-8	5-Hydroxy-7-methoxy-3-(4-hydroxybenzylidene)chroman-4	298.29	10mM	DMSO
303008-81-3	Pandamarilactonine B	317.38	10mM	DMSO
38412-82-7	3,4,4',7-Tetrahydroxyflavan	274.269	10mM	DMSO
90582-44-8	Sorghumol	426.717	10mM	DMSO
90582-47-1	Sorghumol acetate	468.754	10mM	DMSO
34168-56-4	Catalponol	230.302	10mM	DMSO
1221262-77-6	Meliasenin B	468.668	10mM	DMSO
No	12-Ethoxyabietic acid	346.504	10mM	DMSO
34274-91-4	Gardneramine	412.479	10mM	DMSO
20716-98-7	Norlichexanthone	258.226	10mM	DMSO
24173-71-5	Kobusone	222.323	10mM	DMSO
22478-65-5	18-Norabieta-8,11,13-trien-4-ol	272.425	10mM	DMSO
107160-24-7	Pyrocincholic acid methyl ester	456.7	10mM	DMSO
4339-72-4	3-O-Acetyloleanolic acid	498.737	10mM	DMSO
6909-19-9	2-Caren-10-ol	152.233	10mM	DMSO
142647-71-0	Macrocarpal D	472.614	10mM	DMSO
773850-91-2	Drimiopsin D	318.278	10mM	DMSO
3682-02-8	Isohemiphloin	434.393	10mM	DMSO
2259-07-6	Epifriedelanol acetate	470.77	10mM	DMSO
3772-55-2	Dehydroabietinol	286.452	10mM	DMSO
119725-19-8	2-Epitormentic acid	488.699	10mM	DMSO
85051-41-8	Norviburtinal	146.143	10mM	DMSO
33980-71-1	7-Oxodehydroabietinol	300.435	10mM	DMSO
34818-83-2	4'-Demethyleucomin	284.263	10mM	DMSO
107585-77-3	4'-Demethyl-3,9-dihydroeucomin	286.279	10mM	DMSO

1585-68-8	Catalpalactone	258.269	10mM	DMSO
221899-21-4	Macrocarpal N	486.597	10mM	DMSO
142628-54-4	Macrocarpal E	472.614	10mM	DMSO
773850-90-1	Drimiopsin C	288.252	10mM	DMSO
1740-19-8	Dehydroabietic acid	300.435	10mM	DMSO
35897-92-8	Ligustroside	524.514	10mM	DMSO
25645-19-6	Preisocalamendiol	220.35	10mM	DMSO
56473-67-7	4,9-Dihydroxy-alpha-lapachone	274.269	10mM	DMSO
35241-80-6	9-Methoxy-alpha-lapachone	272.296	10mM	DMSO
57906-31-7	19-Nor-4-hydroxyabieta-8,11,13-trien-7-one	286.409	10mM	DMSO
6380-24-1	cis-Methylisoeugenol	178.228	10mM	DMSO
32602-81-6	Kaempferol 3-neohesperidoside	594.518	10mM	DMSO
857672-34-5	Longistylumphylline A	367.481	10mM	DMSO
770721-33-0	6-Feruloylcatalpol	538.498	10mM	DMSO
2189-80-2	Taraxeryl acetate	468.754	10mM	DMSO
107585-75-1	3'-Hydroxy-3,9-dihydroeucomin	316.305	10mM	DMSO
7362-39-2	p-Coumaric acid ethyl ester	192.211	10mM	DMSO
874201-05-5	Daphnilongeranin A	383.481	10mM	DMSO
175556-08-8	Longifloroside A	534.552	10mM	DMSO
1159913-80-0	Abiesadine N	330.461	10mM	DMSO
922522-15-4	Daphnilongeridine	513.752	10mM	DMSO
4666-84-6	Cryptomeridiol	240.382	10mM	DMSO
6736-85-2	Catalposide	482.435	10mM	DMSO
179388-53-5	Macrocarpal H	472.614	10mM	DMSO
No	Quercitrin 3',4',2",3",4"-pentaacetate	658.56	10mM	DMSO
38916-91-5	erythro-Guaiacylglycerol	214.215	10mM	DMSO
524-12-9	Wedelolactone	314.246	10mM	DMSO
129488-34-2	3,4-Diacetoxycinnamamide	263.246	10mM	DMSO
70389-96-7	4(15),11-Oppositadien-1-ol	220.35	10mM	DMSO
19716-26-8	Stigmasterol glucoside	574.831	10mM	DMSO
15486-24-5	Eleutheroside C	208.209	10mM	DMSO
638-97-1	β-Amyrone	424.702	10mM	DMSO
16566-88-4	Methyl 6-acetoxyangolensate	528.591	10mM	DMSO
67600-94-6	Hydrangenol 8-O-glucoside	418.394	10mM	DMSO
480-47-7	Hydrangenol	256.253	10mM	DMSO
113270-98-7	(3R)-Hydrangenol 8-O-glucoside pentaacetate	628.577	10mM	DMSO
112047-91-3	p-Vinylphenyl O-[beta-D-apiofuranosyl-(1-6)]-beta-D-gluco	414.404	10mM	DMSO
81968-62-9	4(15),5,10(14)-Germacratrien-1-ol	220.35	10mM	DMSO
19865-87-3	Cabralealactone	414.621	10mM	DMSO
32619-42-4	Oleuropein	540.514	10mM	DMSO
22255-07-8	Methyl 6-hydroxyangolensate	486.554	10mM	DMSO
70206-70-1	8alpha-Hydroxy-alpha-gurjunene	220.35	10mM	DMSO
29836-27-9	Shanzhiside	392.355	10mM	DMSO
1220508-29-1	Khayalenoid E	526.575	10mM	DMSO
118627-52-4	Epivogeloside	388.366	10mM	DMSO
327601-97-8	Macrocarpal L	472.614	10mM	DMSO
13059-93-3	alpha-Terthienylmethanol	278.413	10mM	DMSO
4707-33-9	alpha-Lapachone	242.27	10mM	DMSO
15297-92-4	Dehydro-alpha-lapachone	240.254	10mM	DMSO
93078-83-2	8-O-Demethyl-7-O-methyl-3,9-dihydropunctatin	316.305	10mM	DMSO
1229005-35-9	Tatarinoid A	240.252	10mM	DMSO
680617-50-9	Megastigm-7-ene-3,5,6,9-tetraol	244.327	10mM	DMSO
1390-72-3	Catalpin	322.31	10mM	DMSO
4460-86-0	2,4,5-Trimethoxybenzaldehyde	196.2	10mM	DMSO
326594-34-7	Fraxamoside	538.498	10mM	DMSO
178600-68-5	Oleoside	390.339	10mM	DMSO
1092555-02-6	Paxiphylline D	383.481	10mM	DMSO
35833-70-6	Cabraleahydroxylactone acetate	458.673	10mM	DMSO

480-23-9	Orobol	286.236	10mM	DMSO
477-57-6	Isotetrandrine	622.75	10mM	DMSO
478-61-5	Berbamine	608.723	10mM	DMSO
1220891-22-4	3,4-Dihydro-3,4-dihydroxynaphthalen-1(2H)-one	178.185	10mM	DMSO
22333-58-0	9-Hydroxy-alpha-lapachone	258.269	10mM	DMSO
1226-22-8	Garbanzol	272.253	10mM	DMSO
16274-33-2	3,4-Dihydro-2,2-dimethyl-2H-naphtho[1,2-b]pyran	212.287	10mM	DMSO
16274-34-3	1-Hydroxy-2-prenylnaphthalene	212.287	10mM	DMSO
1189801-51-1	1-O-Deacetyl-2alpha-hydroxykhayanolide E	532.536	10mM	DMSO
185414-25-9	Corchoionoside C	386.437	10mM	DMSO
133538-77-9	Apigenin 4'-O-rhamnoside	416.378	10mM	DMSO
No	Chlorantholide B	246.302	10mM	DMSO
173991-81-6	22-Hydroxy-3-oxo-12-ursen-30-oic acid	470.684	10mM	DMSO
89199-94-0	Fraxiresinol 1-O-glucoside	566.551	10mM	DMSO
142542-89-0	Cimidahurinine	316.304	10mM	DMSO
577976-26-2	4-(2-Hydroxy-1-methoxyethyl)-1,2-benzenediol	184.189	10mM	DMSO
218290-59-6	Macrocarpal K	472.614	10mM	DMSO
911714-91-5	Chrysothol	238.366	10mM	DMSO
483-91-0	Calycanthoside	384.335	10mM	DMSO
13040-46-5	Paulownin	370.353	10mM	DMSO
265644-24-4	3-Hydroxycatalponol	246.302	10mM	DMSO
6713-27-5	Moronic acid	454.684	10mM	DMSO
93-39-0	Skimmin	324.283	10mM	DMSO
152110-17-3	Teuclatriol	256.381	10mM	DMSO
10391-09-0	Nodosin	362.417	10mM	DMSO
3198-49-0	Ethyl glucoside	208.209	10mM	DMSO
301530-12-1	Seneganolide	470.512	10mM	DMSO
90852-99-6	Ptelatoside B	428.43	10mM	DMSO
941227-27-6	1,3,5-Cadinatriene-3,8-diol	234.334	10mM	DMSO
77949-42-9	Longikaurin E	390.47	10mM	DMSO
276870-26-9	Megastigm-7-ene-3,5,6,9-tetraol	244.327	10mM	DMSO
No	Catalponol methylthiomethyl ether	290.42	10mM	DMSO
3568-90-9	Deoxylapachol	226.27	10mM	DMSO
72514-90-0	Specioside	508.472	10mM	DMSO
476682-97-0	6,9,10-Trihydroxy-7-megastigmen-3-one	242.311	10mM	DMSO
179388-54-6	Macrocarpal I	490.629	10mM	DMSO
179603-47-5	Macrocarpal J	490.629	10mM	DMSO
80434-33-9	gamma-Diasarone	416.507	10mM	DMSO
130855-22-0	ent-kaurane-3,16,17-triol	322.482	10mM	DMSO
1043629-23-7	Tetrahydroxysqualene	474.716	10mM	DMSO
54081-48-0	Isoastilbin	450.393	10mM	DMSO
1802-12-6	Phytolaccagenin	532.709	10mM	DMSO
112667-09-1	Erigeside C	360.313	10mM	DMSO
137941-45-8	Arillatose B	518.465	10mM	DMSO
54354-62-0	Decarine	319.311	10mM	DMSO
No	Glochidionionol C	224.296	10mM	DMSO
5027-76-9	Oleuropeic acid	184.232	10mM	DMSO
1187303-40-7	Cuniloside B	512.59	10mM	DMSO
577-56-0	2-Acetylbenzoic acid	164.158	10mM	DMSO
54299-52-4	2',4'-Dihydroxy-3',6'-dimethoxydihydrochalcone	302.322	10mM	DMSO
35878-41-2	Vestitol	272.296	10mM	DMSO
58436-28-5	Dihydroresveratrol	230.259	10mM	DMSO
100432-87-9	Dihydroresveratrol 3-O-glucoside	392.4	10mM	DMSO
67023-81-8	Ohchinin acetate	644.751	10mM	DMSO
25330-21-6	Isocalamendiol	238.366	10mM	DMSO
493-95-8	Savinin	352.337	10mM	DMSO
137887-25-3	6-O-Feruloylglucose	356.325	10mM	DMSO
155418-97-6	4,5-Dihydroblumenol A	226.312	10mM	DMSO

510-30-5	Echinocystic acid	472.7	10mM	DMSO
93767-25-0	Jangomolide	468.496	10mM	DMSO
36190-95-1	3'-O-Methylorobol	300.263	10mM	DMSO
75590-33-9	Kaerophyllin	368.38	10mM	DMSO
No	Chlorantholide A	244.286	10mM	DMSO
164022-75-7	Clerodenoside A	736.714	10mM	DMSO
29838-67-3	Astilbin	450.393	10mM	DMSO
147517-06-4	Thunberginol C	272.253	10mM	DMSO
35833-72-8	3-Epicabraleahydroxylactone	416.636	10mM	DMSO
155060-48-3	24,25-Dihydroxycycloartan-3-one	458.716	10mM	DMSO
479-91-4	Casticin	374.341	10mM	DMSO
831222-78-7	5-Hydroxymethyl-7-methoxybenzofuran	178.185	10mM	DMSO
118930-92-0	5-(3-Hydroxypropyl)-7-methoxybenzofuran	206.238	10mM	DMSO
48236-96-0	Tetrahydroamentoflavone	542.49	10mM	DMSO
No	5,6-O-Isopropylidene-phlorigidoside B	504.482	10mM	DMSO
36052-37-6	Alpinetin	270.28	10mM	DMSO
No	Chloranthollide D	262.301	10mM	DMSO
1231208-53-9	4,8-Dihydroxyeudesm-7(11)-en-12,8-olide	266.333	10mM	DMSO
1020074-97-8	20,24-Epoxy-24-methoxy-23(24-25)abeo-dammaran-3-one	472.743	10mM	DMSO
501-96-2	Rhododendrol	166.217	10mM	DMSO
5096-57-1	Canadine	339.385	10mM	DMSO
No	10-O-Acetylisocalamendiol	280.402	10mM	DMSO
157659-20-6	Prenylpiperitol	424.486	10mM	DMSO
64421-28-9	Shanzhiside methyl ester	406.382	10mM	DMSO
70191-83-2	Isotetrandrine N-2'-oxide	638.749	10mM	DMSO
678138-59-5	2,3,2",3"-Tetrahydroochnaflavone	542.49	10mM	DMSO
182138-70-1	Nyssoside	490.371	10mM	DMSO
14965-20-9	Chrysosplenol D	360.315	10mM	DMSO
366450-46-6	Semialactone	468.668	10mM	DMSO
72061-63-3	Sepinol	318.278	10mM	DMSO
61186-24-1	Grandifloroside	538.498	10mM	DMSO
126223-29-8	Agrimonolide 6-O-glucoside	476.473	10mM	DMSO
197307-49-6	11-Dehydroxygrevilloside B	342.384	10mM	DMSO
57586-98-8	Cycloartane-3,24,25-triol	460.732	10mM	DMSO
20725-03-5	Fustin	288.252	10mM	DMSO
29748-10-5	Loganetin	228.242	10mM	DMSO
1253740-09-8	6-Acetonyl-N-methyl-dihydrodecarine	391.417	10mM	DMSO
67828-62-0	Ethyl 2,4-dihydroxyphenylacetate	196.2	10mM	DMSO
117479-87-5	Sesamoside	420.365	10mM	DMSO
20243-59-8	Hydroxygenkwanin	300.263	10mM	DMSO
1242085-06-8	12-Ursene-3,16,22-triol	458.716	10mM	DMSO
33606-81-4	Myricanol	358.428	10mM	DMSO
127-22-0	Taraxerol	426.717	10mM	DMSO
4382-33-6	Dihydrorobinetin	304.252	10mM	DMSO
520-33-2	Hesperetin	302.279	10mM	DMSO
75069-59-9	20,24-Dihydroxydammar-25-en-3-one	458.716	10mM	DMSO
4382-34-7	Robtin	288.252	10mM	DMSO
83529-71-9	Anisofolin A	724.663	10mM	DMSO
84-79-7	Lapachol	242.27	10mM	DMSO
82003-90-5	Daurichromenic acid	370.482	10mM	DMSO
20133-19-1	1-(3,4-Dimethoxyphenyl)propane-1,2-diol	212.242	10mM	DMSO
35349-68-9	9-Hydroxycalabaxanthone	408.444	10mM	DMSO
85372-70-9	5,19-Epoxy-19,25-dimethoxycucurbita-6,23-dien-3-ol	500.753	10mM	DMSO
104953-08-4	Ethyl beta-D-ribo-hex-3-ulopyranoside	206.193	10mM	DMSO
119767-00-9	3-Furfuryl 2-pyrrolecarboxylate	191.183	10mM	DMSO
1079941-35-7	Myricananin A	344.402	10mM	DMSO
26296-50-4	Ficaprenol 11	767.303	10mM	DMSO
268541-26-0	Triptohypol F	456.743	10mM	DMSO

168293-10-5	C-Veratroylglycol	212.199	10mM	DMSO
No	9,11,13-Octadecatriynoic acid methyl ester	286.409	10mM	DMSO
585534-03-8	Confluentin	326.472	10mM	DMSO
202596-22-3	Calyxin H	566.64	10mM	DMSO
1190225-48-9	Sarcandrone B	554.587	10mM	DMSO
113558-03-5	1,2,3,19-Tetrahydroxy-12-ursen-28-oic acid	504.699	10mM	DMSO
94530-87-7	9(11),12-Oleanadien-3-ol	424.702	10mM	DMSO
62014-81-7	p-Menthane-1,2,8-triol	188.264	10mM	DMSO
41137-85-3	Platyphyllonol	314.376	10mM	DMSO
207446-90-0	Arteannuin M	268.349	10mM	DMSO
944804-58-4	Rhuscholide A	462.663	10mM	DMSO
No	Chlorantholide E	278.3	10mM	DMSO
875585-30-1	ent-14,15-Dinor-13-oxolabda-8(17),11-dien-18-oic acid	290.397	10mM	DMSO
83915-59-7	13-Hydroxylabda-8(17),14-dien-18-oic acid	320.466	10mM	DMSO
1039673-32-9	15-Nor-14-oxolabda-8(17),12-dien-18-oic acid	304.424	10mM	DMSO
30359-01-4	Centrolobol	300.392	10mM	DMSO
56973-65-0	Platyphyllenone	296.36	10mM	DMSO
34316-15-9	Chelerythrine	348.371	10mM	DMSO
58-08-2	Caffeine	194.191	10mM	DMSO
154418-16-3	5,5'-Dimethoxylariciresinol 4-O-glucoside	582.594	10mM	DMSO
22805-15-8	3-(4-Hydroxy-3,5-dimethoxyphenyl)-1,2-propanediol	228.242	10mM	DMSO
116384-26-0	3',4',7-Trimethoxyflavan	300.349	10mM	DMSO
60337-67-9	9-O-Feruloyllariciresinol	536.57	10mM	DMSO
5373-11-5	Luteolin 7-glucoside	448.377	10mM	DMSO
61303-13-7	Isoacteoside	624.587	10mM	DMSO
14215-86-2	Sweroside	358.34	10mM	DMSO
5289-74-7	20-Hydroxyecdysone	480.634	10mM	DMSO
848669-09-0	Stigmasta-4,22,25-trien-3-one	408.659	10mM	DMSO
848669-08-9	Stigmasta-4,25-dien-3-one	410.675	10mM	DMSO
23141-27-7	Vincosamide	498.525	10mM	DMSO
852638-61-0	Heteronoside	790.675	10mM	DMSO
148044-47-7	25-Hydroxycycloart-23-en-3-one	440.701	10mM	DMSO
490-31-3	Robinetin	302.236	10mM	DMSO
32492-74-3	Myricanone	356.412	10mM	DMSO
17884-88-7	Myricadiol	442.717	10mM	DMSO
70051-38-6	4,10-Aromadendranediol	238.366	10mM	DMSO
81910-39-6	5,19-Epoxy-25-methoxycucurbita-6,23-dien-3-ol	470.727	10mM	DMSO
934739-29-4	Karavilagenin D	470.684	10mM	DMSO
7608-44-8	Artocarpin	436.497	10mM	DMSO
159623-48-0	3,4-Secotirucalla-4(28),7,24-triene-3,26-dioic acid	470.684	10mM	DMSO
33390-41-9	8-Deoxygartanin	380.434	10mM	DMSO
41137-87-5	Hirsutenone	328.359	10mM	DMSO
76035-62-6	3-Epikatonic acid	456.7	10mM	DMSO
55497-79-5	Myriceric acid B	634.842	10mM	DMSO
31298-06-3	Arjunic acid	488.699	10mM	DMSO
164991-53-1	Calyxin B	582.64	10mM	DMSO
906-33-2	Neochlorogenic acid	354.309	10mM	DMSO
69120-07-6	Moracin D	308.328	10mM	DMSO
144629-84-5	22-Hydroxy-3-oxoolean-12-en-29-oic acid	470.684	10mM	DMSO
465-13-4	Neritaloside	592.718	10mM	DMSO
142279-41-2	Shizukaol C	634.713	10mM	DMSO
40957-99-1	Medioresinol	388.411	10mM	DMSO
482-35-9	Isoquercitrin	464.376	10mM	DMSO
No	2,3-Di-O-methylthiomethyleuscaphic acid	608.935	10mM	DMSO
61671-56-5	Neridienone B	344.445	10mM	DMSO
18810-25-8	Odoroside H	534.681	10mM	DMSO
129212-92-6	(2S,3S)-(-)-Glucodistylin	466.392	10mM	DMSO
27661-51-4	Leucoside	580.492	10mM	DMSO

405281-76-7	Dadahol A	698.712	10mM	DMSO
59204-61-4	beta-Dihydroplumericinic acid	278.257	10mM	DMSO
52525-35-6	Quercetin 3-O-robinobioside	610.518	10mM	DMSO
480-36-4	Linarin	592.545	10mM	DMSO
6018-40-2	Corypalmine	341.401	10mM	DMSO
No	Chlorantholide F	278.3	10mM	DMSO
51005-44-8	Minecoside	538.498	10mM	DMSO
56222-03-8	Porson	386.438	10mM	DMSO
34509-52-9	Myricanol triacetate	484.538	10mM	DMSO
136807-41-5	6-O-Cinnamoylcatalpol	492.473	10mM	DMSO
211126-61-3	Rubranol	332.391	10mM	DMSO
956869-95-7	Euscaphin B	258.354	10mM	DMSO
552-57-8	Isorhoifolin	578.519	10mM	DMSO
41137-86-4	Hirsutanonol	346.374	10mM	DMSO
572-31-6	Engeletin	434.393	10mM	DMSO
156368-84-2	Ehretioside B	311.287	10mM	DMSO
144049-72-9	6-O-Syringoylajugol	528.503	10mM	DMSO
62312-55-4	5-Hydroxy-2-pyrrolidinone	101.104	10mM	DMSO
31712-49-9	Hesperetin 7-O-glucoside	464.419	10mM	DMSO
564-14-7	Hop-17(21)-en-3-ol	426.717	10mM	DMSO
511-89-7	Plumieride	470.424	10mM	DMSO
84799-31-5	5-Epilithospermoside	329.303	10mM	DMSO
80416-52-0	13-O-p-Coumaroylplumieride	616.567	10mM	DMSO
15404-80-5	Isonormangostin	396.433	10mM	DMSO
112649-48-6	BR-Xanthone A	396.433	10mM	DMSO
120051-54-9	Meridinol	370.353	10mM	DMSO
147714-71-4	6-O-(3",4"-Dimethoxycinnamoyl)catalpol	552.524	10mM	DMSO
121710-02-9	6-O-p-Methoxycinnamoylcatalpol	522.499	10mM	DMSO
No	Chloranthollide C	246.302	10mM	DMSO
936006-11-0	5-(6-Hydroxybenzofuran-2-yl)-2-(3-methylbut-1-enyl)benze	310.344	10mM	DMSO
39011-92-2	Nuezhenide	686.655	10mM	DMSO
77658-46-9	ent-11,16-Epoxy-15-hydroxykauran-19-oic acid	334.45	10mM	DMSO
149250-48-6	De-4'-O-methylyangambin	432.464	10mM	DMSO
162059-94-1	Myriceric acid C	796.984	10mM	DMSO
6619-95-0	Deacetylxylopic acid	318.45	10mM	DMSO
17526-15-7	Xanthorin	300.263	10mM	DMSO
50932-19-9	Verminoside	524.471	10mM	DMSO
108-46-3	Resorcinol	110.111	10mM	DMSO
1007387-95-2	1-O-Deacetylkhayanolide E	516.537	10mM	DMSO
526-87-4	Conduritol A	146.141	10mM	DMSO
54963-52-9	2-Oxopomolic acid	486.683	10mM	DMSO
69120-06-5	Moracin C	310.344	10mM	DMSO
301-19-9	Robinin	740.659	10mM	DMSO
No	7-O-Demethyl-3-isomangostin hydrate	414.448	10mM	DMSO
90332-92-6	Shizukolidol	248.318	10mM	DMSO
112693-21-7	Oleonuezhenide	1073.01	10mM	DMSO
56486-94-3	Steppogenin	288.252	10mM	DMSO
142474-52-0	Glyasperin A	422.47	10mM	DMSO
13956-29-1	Cannabidiol	314.462	10mM	DMSO
168254-96-4	Evofolin B	318.321	10mM	DMSO
477-84-9	Damnacanthal	282.248	10mM	DMSO
62949-79-5	Mulberrin	422.47	10mM	DMSO
119318-15-9	Olean-12-ene-3,24-diol	442.717	10mM	DMSO
471-66-9	α-Boswellic acid	456.7	10mM	DMSO
189351-15-3	Corchoionol C	224.296	10mM	DMSO
1616-93-9	β-Amyrin acetate	468.754	10mM	DMSO
130838-00-5	Scoparinol	426.588	10mM	DMSO
76472-88-3	Morachalcone A	340.37	10mM	DMSO

39903-21-4	29-Hydroxyfriedelan-3-one	442.717	10mM	DMSO
480-43-3	Isosakuranetin	286.279	10mM	DMSO
7460-43-7	Rubiadin 1-methyl ether	268.264	10mM	DMSO
472-30-0	Masticadienolic acid	456.7	10mM	DMSO
56421-12-6	Methyl eichlerianate	488.742	10mM	DMSO
442851-27-6	Methyl isodrimeninol	250.376	10mM	DMSO
62218-23-9	3-O-Methylducheside A	462.36	10mM	DMSO
532-91-2	Coixol	165.146	10mM	DMSO
6926-14-3	8-O-Acetylharpagide	406.382	10mM	DMSO
77741-58-3	1,7-Dihydroxy-3-methoxy-2-prenylxanthone	326.343	10mM	DMSO
3542-72-1	Norathyriol	260.199	10mM	DMSO
72537-20-3	Polygonal	222.323	10mM	DMSO
117-02-2	Rubiadin	254.238	10mM	DMSO
87480-84-0	Dihydroajugapitin	552.654	10mM	DMSO
62596-29-6	Morusin	420.454	10mM	DMSO
1180-35-4	Acetylepipodophyllotoxin	456.442	10mM	DMSO
5282-14-4	Olean-12-ene-3,11-diol	442.717	10mM	DMSO
124168-04-3	6-O-Vanilloylajugol	498.477	10mM	DMSO
83945-57-7	4-Epicommunic acid	302.451	10mM	DMSO
120-14-9	Veratraldehyde	166.174	10mM	DMSO
3420-72-2	Flavokawain A	314.333	10mM	DMSO
961-29-5	Isoliquiritigenin	256.253	10mM	DMSO
20245-39-0	1,3,7-Trihydroxy-2-prenylxanthone	312.317	10mM	DMSO
816456-90-3	Canusesnol A	250.333	10mM	DMSO
60129-63-7	Paniculoside I	480.591	10mM	DMSO
93796-20-4	3-Prenyl-2,4,6-trihydroxybenzophenone	298.333	10mM	DMSO
132586-69-7	15-Demethylplumieride	456.397	10mM	DMSO
3570-40-9	Albaspidin AA	404.41	10mM	DMSO
466-09-1	Uzarigenin	374.514	10mM	DMSO
19186-35-7	Deoxypodophyllotoxin	398.406	10mM	DMSO
479-21-0	Cotoin	244.243	10mM	DMSO
610778-85-3	Isobonducellin	282.291	10mM	DMSO
80508-42-5	Tenacigenin B	364.476	10mM	DMSO
1174017-37-8	Artoheterophyllin B	504.571	10mM	DMSO
No	9-Hydroxycalabaxanthone hydrate	426.459	10mM	DMSO
495-02-3	Auraptene	298.376	10mM	DMSO
90996-27-3	8-Methoxybonducellin	312.317	10mM	DMSO
40456-50-6	Yatein	400.422	10mM	DMSO
26271-33-0	2,3',4,6-Tetrahydroxybenzophenone	246.215	10mM	DMSO
808769-54-2	3,22-Dihydroxyolean-12-en-29-oic acid	472.7	10mM	DMSO
19131-13-6	6-Deoxy-3-O-methyl-β-allopyranosyl(1→4)-β-cymaronic ac	320.336	10mM	DMSO
110-15-6	Succinic acid	118.088	10mM	DMSO
201534-09-0	Triptocallic acid D	472.7	10mM	DMSO
112652-46-7	Fragransin A2	344.402	10mM	DMSO
184587-72-2	Mangostanol	426.459	10mM	DMSO
No	8-Isomulberrin hydrate	440.486	10mM	DMSO
27013-91-8	α-Hederin	750.956	10mM	DMSO
488-76-6	vibo-Quercitol	164.156	10mM	DMSO
3080-20-4	β-Anhydrouzarigenin	356.498	10mM	DMSO
60-82-2	Phloretin	274.269	10mM	DMSO
85122-21-0	2-Methoxystypandrone	260.242	10mM	DMSO
92609-77-3	O-Demethylforbexanthone	326.3	10mM	DMSO
16100-84-8	Dihydroperaksine	312.406	10mM	DMSO
942609-65-6	1,5,15-Tri-O-methylmorindol	328.316	10mM	DMSO
74336-91-7	4',4'''-Di-O-methylcupressuflavone	566.511	10mM	DMSO
51995-99-4	Dehydroespeletone	232.275	10mM	DMSO
6750-10-3	Fuegin	266.333	10mM	DMSO
465-16-7	Oleandrin	576.718	10mM	DMSO

864516-31-4	Nigrolineaxanthone V	408.444	10mM	DMSO
519-34-6	Maclurin	262.215	10mM	DMSO
25577-04-2	1,6,7-Trihydroxyxanthone	244.2	10mM	DMSO
451478-47-0	10-Hydroxydihydroperaksine	328.405	10mM	DMSO
68799-41-7	6-Acetyl-2,2-dimethylchroman-4-one	218.248	10mM	DMSO
118169-27-0	6"-O-Acetylastragalin	490.414	10mM	DMSO
86606-14-6	6-Benzoyl-5,7-dihydroxy-2,2-dimethylchromane	298.333	10mM	DMSO
No	3-(3-Hydroxy-3-methylbutanyl)-2,4,6-trihydroxybenzophen	316.348	10mM	DMSO
63565-07-1	8-Benzoyl-5,7-dihydroxy-2,2-dimethylchromane	298.333	10mM	DMSO
22136-74-9	Podocarpusflavone A	552.485	10mM	DMSO
62949-93-3	Morusinol	438.47	10mM	DMSO
58469-06-0	N-(2-Hydroxy-4-methoxyphenyl)acetamide	181.189	10mM	DMSO
7121-99-5	Erythroxytriol P	324.498	10mM	DMSO
64032-49-1	Torachrysone 8-O-glucoside	408.399	10mM	DMSO
617722-55-1	Methyl 2-(5-acetyl-2,3-dihydrobenzofuran-2-yl)propenoate	246.259	10mM	DMSO
35943-38-5	Pteroside D	410.458	10mM	DMSO
20675-51-8	Cannabichromene	314.462	10mM	DMSO
22649-04-3	Torachrysone	246.259	10mM	DMSO
617722-56-2	Methyl 2-(6-acetyl-5-hydroxy-2,3-dihydrobenzofuran-2-yl)p	262.258	10mM	DMSO
36450-01-8	6-Hydroxystigmasta-4,22-dien-3-one	426.674	10mM	DMSO
218780-16-6	12-Hydroxyisodrimenin	250.333	10mM	DMSO
22415-24-3	3-Epiturraeanthin	514.736	10mM	DMSO
1025023-05-5	Periglaucine B	373.4	10mM	DMSO
42895-58-9	14-Deoxy-11,12-didehydroandrographolide	332.434	10mM	DMSO
970-74-1	Epigallocatechin	306.267	10mM	DMSO
5631-68-5	3-(2,4-Dihydroxyphenyl)propionic acid	182.173	10mM	DMSO
51828-10-5	2'-O-Methylisoliquiritigenin	270.28	10mM	DMSO
465-99-6	Hederagenin	472.7	10mM	DMSO
110064-50-1	7-Hydroxy-3-(4-hydroxybenzylidene)chroman-4-one	268.264	10mM	DMSO
19888-34-7	Humulene epoxide II	220.35	10mM	DMSO
17422-90-1	Methyl 3-(2,4-dihydroxyphenyl)propionate	196.2	10mM	DMSO
52611-75-3	Epipterosin L	264.317	10mM	DMSO
25654-31-3	Cannabigerol	316.478	10mM	DMSO
144881-19-6	Junipediol B	196.2	10mM	DMSO
3570-62-5	Moslosooflavone	298.29	10mM	DMSO
113981-49-0	5-Hydroxy-7,8-dimethoxyflavanone	300.306	10mM	DMSO
62014-87-3	Helichrysetin	286.279	10mM	DMSO
69804-59-7	2,7-Dihydroxy-2H-1,4-benzoxazin-3(4H)-one	181.145	10mM	DMSO
139561-95-8	Epicannabidiol hydrate	332.477	10mM	DMSO
1083200-79-6	1,7-Bis(4-hydroxyphenyl)hept-1-en-3-one	296.36	10mM	DMSO
158500-59-5	Cnidioside B methyl ester	412.388	10mM	DMSO
No	6'-Hydroxy-7'-ethoxybergamottin	400.465	10mM	DMSO
213552-47-7	Drim-7-ene-11,12-diol acetonide	278.43	10mM	DMSO
221257-06-3	7,4'-Di-O-methylapigenin 5-O-xylosylglucoside	592.545	10mM	DMSO
1088-17-1	Isomeranzin	260.285	10mM	DMSO
74048-71-8	Lancifolin C	372.455	10mM	DMSO
34169-70-5	Pterosin D	248.318	10mM	DMSO
190906-61-7	Triptocallic acid A	472.7	10mM	DMSO
904665-71-0	Daturataturin A aglycone	454.598	10mM	DMSO
221289-31-2	Lethedioside A	622.571	10mM	DMSO
920502-42-7	Tenacigenoside A	668.812	10mM	DMSO
113122-54-6	3-Deoxysappanone B	286.279	10mM	DMSO
495-32-9	Nodakenetin	246.259	10mM	DMSO
449729-89-9	(+)-S-Myricanol glucoside	520.569	10mM	DMSO
6587-37-7	3-Epiglochidiol diacetate	526.79	10mM	DMSO
51857-11-5	7-O-Methyleriodictyol	302.279	10mM	DMSO
60102-29-6	Isosativan	286.322	10mM	DMSO
41347-49-3	Anhydrotuberosin	320.339	10mM	DMSO

5928-26-7	Sissotrin	446.404	10mM	DMSO
142566-61-8	Calanolide E	388.454	10mM	DMSO
350986-74-2	Dendocarbin A	250.333	10mM	DMSO
146450-83-1	Camaric acid	568.784	10mM	DMSO
31427-08-4	Isotachioside	302.277	10mM	DMSO
No	7'-O-Ethylmarmin	360.444	10mM	DMSO
70411-27-7	Dihydrotamarixetin	318.278	10mM	DMSO
130288-60-7	Rubiarbonol B	458.716	10mM	DMSO
No	No	384.379	10mM	DMSO
No	No	478.532	10mM	DMSO
No	Astragalin 4',2",3",4",6"-pentaacetate	658.56	10mM	DMSO
487-52-5	Butein	272.253	10mM	DMSO
93915-36-7	Hirsutanonol 5-O-glucoside	508.515	10mM	DMSO
76996-27-5	Garcinone C	414.448	10mM	DMSO
104778-16-7	4-O-Methylsappanol	318.321	10mM	DMSO
474-07-7	Brazilin	286.279	10mM	DMSO
1223097-20-8	6"-O-acetylisovitexin	474.414	10mM	DMSO
1111897-60-9	7,3',4'-Trihydroxy-3-benzyl-2H-chromene	270.28	10mM	DMSO
144223-70-1	Dehydroadynerigenin glucosyldigitaloside	692.79	10mM	DMSO
41744-39-2	Acuminatin	340.413	10mM	DMSO
603-61-2	Tamarixetin	316.262	10mM	DMSO
79995-67-8	Blumeatin B	332.305	10mM	DMSO
924910-83-8	21-Deoxyneridienone B	328.445	10mM	DMSO
4674-50-4	Nootkatone	218.335	10mM	DMSO
4773-96-0	Mangiferin	422.34	10mM	DMSO
72944-06-0	30-Hydroxylup-20(29)-en-3-one	440.701	10mM	DMSO
34425-25-7	Lyoniside	552.568	10mM	DMSO
34169-69-2	Pterosin Z	232.318	10mM	DMSO
94344-54-4	Sappanchalcone	286.279	10mM	DMSO
1025023-04-4	Periglaucine A	373.4	10mM	DMSO
80453-44-7	Padmatin	318.278	10mM	DMSO
945259-61-0	4-Hydroxy-2-methoxyphenol 1-O-(6-O-syringoyl)glucoside	482.435	10mM	DMSO
57296-22-7	Boehmenan	712.738	10mM	DMSO
61262-81-5	Cannabispiran	246.302	10mM	DMSO
482-68-8	Sarpagine	310.39	10mM	DMSO
176519-75-8	8-Hydroxyodoroside A	534.681	10mM	DMSO
118024-26-3	Blumeatin	302.279	10mM	DMSO
529-40-8	Ombuin	330.289	10mM	DMSO
486-60-2	Bergaptol	202.163	10mM	DMSO
84104-80-3	Wilforlide A acetate	496.721	10mM	DMSO
99-18-3	Prunasin	295.288	10mM	DMSO
No	6'-O-Acetylpaniculoside II	538.627	10mM	DMSO
14957-38-1	Marmin	332.391	10mM	DMSO
70677-47-3	Canniprene	342.429	10mM	DMSO
64125-60-6	Longistylin C	294.387	10mM	DMSO
1049674-06-7	8-Hydroxydigitoxigenin	390.513	10mM	DMSO
376361-96-5	7,4'-Dihydroxy-3'-prenylflavan	310.387	10mM	DMSO
99624-27-8	Kazinol B	392.487	10mM	DMSO
32884-36-9	Cajanin	300.263	10mM	DMSO
38242-02-3	β-Amyrenonol	440.701	10mM	DMSO
62043-53-2	Onitisin 2'-O-glucoside	426.457	10mM	DMSO
87686-86-0	6-Hydroxyrubiadin	270.237	10mM	DMSO
521-32-4	Bilobetin	552.485	10mM	DMSO
7622-53-9	5-Deoxycajanin	284.263	10mM	DMSO
102036-29-3	Protosappanin B	304.295	10mM	DMSO
41060-16-6	Skullcapflavone I	314.289	10mM	DMSO
90902-21-9	Broussonin E	288.338	10mM	DMSO
33429-83-3	Quercetin 3,4'-dimethyl ether	330.289	10mM	DMSO

264234-05-1	6',7'-Dihydroxybergamottin	372.412	10mM	DMSO
644967-44-2	Rubianthraquinone	284.263	10mM	DMSO
91269-84-0	Melilotigenin B	454.684	10mM	DMSO
188970-21-0	Melilotigenin C	456.7	10mM	DMSO
10267-31-9	Labd-13-ene-8,15-diol	308.499	10mM	DMSO
522-47-4	Lochnerine	324.417	10mM	DMSO
76947-60-9	Onitin 2'-O-glucoside	410.458	10mM	DMSO
529-55-5	Prunin	434.393	10mM	DMSO
376362-03-7	2'-O-Methylbroussonin C	326.429	10mM	DMSO
82427-77-8	Maglifloenone	386.438	10mM	DMSO
68160-76-9	Nortetraphyllicine	294.391	10mM	DMSO
65597-44-6	Cerberic acid	220.178	10mM	DMSO
No	β-Amyrenonol methylthiomethyl ether	500.819	10mM	DMSO
2935-32-2	Olean-12-ene-3,11-dione	438.685	10mM	DMSO
No	Methyl mandelate glucoside	328.315	10mM	DMSO
767-98-6	Mallorepine	134.135	10mM	DMSO
133360-51-7	Daturataturin A	616.739	10mM	DMSO
10178-31-1	Elliotinol	288.467	10mM	DMSO
1244-58-2	Cannabidiolic acid	358.471	10mM	DMSO
1227375-09-8	Bi-linderone	600.612	10mM	DMSO
320624-68-8	Marmin acetonide	372.455	10mM	DMSO
684217-08-1	6',7'-Dihydroxybergamottin acetonide	412.476	10mM	DMSO
1221-43-8	Auraptenol	260.285	10mM	DMSO
15527-80-7	Peraksine	310.39	10mM	DMSO
51-55-8	Atropine	289.369	10mM	DMSO
213912-46-0	Barbacarpan	322.355	10mM	DMSO
126060-09-1	6-O-Methylcerevisterol	444.69	10mM	DMSO
904667-65-8	Daturametelin I	616.739	10mM	DMSO
138772-01-7	Eugenol rutinoside	472.483	10mM	DMSO
36545-53-6	Cycloheterophyllin	502.555	10mM	DMSO
5356-56-9	β-Amyrenonol acetate	482.738	10mM	DMSO
163136-19-4	Cannabisin F	624.68	10mM	DMSO
61217-80-9	Uzarigenin digitaloside	534.681	10mM	DMSO
64052-90-0	Cannabispirol	248.318	10mM	DMSO
358721-33-2	Apocynol A	224.296	10mM	DMSO
1016974-78-9	Olivil monoacetate	418.437	10mM	DMSO
256445-66-6	Schleicheol 1	444.733	10mM	DMSO
150150-61-1	11,13-Dihydroivalin	250.333	10mM	DMSO
2400-71-7	Pyrocatechol monoglucoside	272.251	10mM	DMSO
20733-94-2	Methyl sinapate	238.237	10mM	DMSO
61117-89-3	Epipterosin L 2'-O-glucoside	426.457	10mM	DMSO
53505-68-3	4,9,9'-Trihydroxy-3,3'-dimethoxy-8,4'-oxyneolignan	362.417	10mM	DMSO
529-59-9	Genistin	432.378	10mM	DMSO
80510-06-1	Grossamide	624.68	10mM	DMSO
33069-62-4	Paclitaxel	853.906	10mM	DMSO
31282-07-2	Raucaffricine	512.552	10mM	DMSO
1265908-20-0	8,14-Epoxyergosta-4,22-diene-3,6-dione	424.615	10mM	DMSO
95456-43-2	Hydroxytuberosone	354.353	10mM	DMSO
66648-44-0	N-Feruloyloctopamine	329.347	10mM	DMSO
84299-80-9	Pterosin D 3-O-glucoside	410.458	10mM	DMSO
858360-61-9	Marsdenoside F	752.885	10mM	DMSO
467-81-2	Rehmannic acid	552.784	10mM	DMSO
160242-09-1	14-Deoxy-11-hydroxyandrographolide	350.449	10mM	DMSO
857297-90-6	3-Hydroxysarpagine	326.39	10mM	DMSO
10236-47-2	Naringin	580.535	10mM	DMSO
869807-57-8	Andropanolide	350.449	10mM	DMSO
66648-45-1	N-p-Coumaroyloctopamine	299.321	10mM	DMSO
125305-73-9	21,24-Epoxycycloartane-3,25-diol	458.716	10mM	DMSO

115040-04-5	29-Norcycloart-23-ene-3,25-diol	428.69	10mM	DMSO
69636-83-5	α-Cannabispiranol	248.318	10mM	DMSO
27215-14-1	Neoandrographolide	480.591	10mM	DMSO
76166-59-1	Derrone	336.338	10mM	DMSO
77988-07-9	Secologanin dimethyl acetal	434.435	10mM	DMSO
166547-20-2	Eurycarpin A	338.354	10mM	DMSO
No	24-Hydroxy-25-ethoxy-3,4-seco-cycloart-4(28)-en-3-oic aci	516.795	10mM	DMSO
73731-87-0	Broussonin A	258.312	10mM	DMSO
73731-86-9	Broussonin B	258.312	10mM	DMSO
191729-44-9	Tenacissoside I	814.955	10mM	DMSO
79114-77-5	Lariciresinol acetate	402.438	10mM	DMSO
952485-00-6	Hydrangenoside A dimethyl acetal	666.71	10mM	DMSO
29307-03-7	Deoxyelephantopin	344.358	10mM	DMSO
135384-00-8	8-Prenyldaidzein	322.355	10mM	DMSO
185213-52-9	Scabertopin	358.385	10mM	DMSO
605-14-1	Serpentinic acid	335.376	10mM	DMSO
7154-01-0	Safrolglycol	196.2	10mM	DMSO
857897-01-9	11,12-Di-O-acetyltenacigenin B	448.549	10mM	DMSO
869384-82-7	14-Deoxy-17-hydroxyandrographolide	352.465	10mM	DMSO
219721-33-2	14-Deoxy-12-hydroxyandrographolide	350.449	10mM	DMSO
No	25-Ethoxy-24-oxo-3,4-secocycloart-4(28)-en-3-oic acid met	514.779	10mM	DMSO
101691-27-4	Barpisoflavone A	300.263	10mM	DMSO
88-14-2	Furan-2-carboxylic acid	112.083	10mM	DMSO
143212-60-6	Dehydroadynerigenin β-neritrioside	838.931	10mM	DMSO
30452-60-9	Cyclomusalenone	424.702	10mM	DMSO
160498-00-0	Bisandrographolide A	664.868	10mM	DMSO
97411-50-2	2',3'-Dehydrosalannol	554.671	10mM	DMSO
94805-83-1	Isolicoflavonol	354.353	10mM	DMSO
205534-17-4	2'',4''-Di-O-(Z-p-coumaroyl)afzelin	724.663	10mM	DMSO
1238116-48-7	Kazinol U	326.386	10mM	DMSO
1110-56-1	Deacetylsalannin	554.671	10mM	DMSO
5945-86-8	Nimbin	540.601	10mM	DMSO
22338-67-6	Grandiflorenic acid	300.435	10mM	DMSO
52275-04-4	3,5-Diprenyl-4-hydroxybenzaldehyde	258.355	10mM	DMSO
349534-70-9	Methyl dodonate A	344.445	10mM	DMSO
7678-85-5	2'-Hydroxydaidzein	270.237	10mM	DMSO
67023-80-7	Ohchinin	602.714	10mM	DMSO
20460-33-7	Epitaraxerol	426.717	10mM	DMSO
263368-91-8	4-Acetyl-3,6,8-trihydroxy-3-methyldihydronaphthalenone	250.247	10mM	DMSO
511-15-9	Totarol	286.452	10mM	DMSO
19941-83-4	Kolavenol	290.483	10mM	DMSO
105608-27-3	Prostephanaberrine	343.374	10mM	DMSO
53526-67-3	Durantoside I	552.524	10mM	DMSO
53526-66-2	Durantoside II	582.55	10mM	DMSO
27856-54-8	Lamiide	422.381	10mM	DMSO
31721-94-5	5,7-Dihydroxychromone	178.142	10mM	DMSO
117614-84-3	1-Hydroxy-1-(4-hydroxy-2-methoxyphenyl)-3-(4-hydroxyph	288.295	10mM	DMSO
191729-43-8	Tenacissoside G	792.949	10mM	DMSO
1268140-15-3	5,7,3'-Trihydroxy-4'-methoxy-8-prenylflavanone	370.396	10mM	DMSO
136196-47-9	3,4'-Dihydroxy-3',5'-dimethoxypropiophenone	226.226	10mM	DMSO
162290-05-3	7,3'-Dihydroxy-4'-methoxyflavan	272.296	10mM	DMSO
110187-11-6	1,5,8-Trihydroxy-3-methoxy-2-prenylxanthone	342.343	10mM	DMSO
61448-03-1	Lup-20(29)-ene-2α,3β-diol	442.717	10mM	DMSO
502-69-2	Phytone	268.478	10mM	DMSO
70387-38-1	6α-Hydroxynidorellol	322.482	10mM	DMSO
263764-83-6	Buergerinin G	184.189	10mM	DMSO
13895-92-6	Rutaretin	262.258	10mM	DMSO
88721-09-9	Adynerigenin β-neritrioside	840.947	10mM	DMSO

349487-98-5	Methyl dodonate A acetate	386.481	10mM	DMSO
118555-84-3	Floribundone 1	566.511	10mM	DMSO
77-60-1	Tigogenin	416.636	10mM	DMSO
919769-83-8	Buergerinin B	202.204	10mM	DMSO
117254-98-5	8α-Hydroxylabda-13(16),14-dien-19-yl p-hydroxycinnamate	452.625	10mM	DMSO
No	6α,7β-Isopropylidenedioxy-abienol	362.546	10mM	DMSO
141973-41-3	14-Deoxy-11,12-didehydroandrographiside	494.575	10mM	DMSO
61854-36-2	Demethoxycapillarisin	286.236	10mM	DMSO
99624-92-7	Uncinatone	326.386	10mM	DMSO
78916-55-9	Deacetylnimbinene	440.529	10mM	DMSO
72058-36-7	Lochnericine	352.427	10mM	DMSO
18609-16-0	Deacetylnimbin	498.565	10mM	DMSO
263368-92-9	4-(cis)-Acetyl-3,6,8-trihydroxy-3-methyldihydronaphthalen	250.247	10mM	DMSO
161753-49-7	12-Oxocalanolide A	368.423	10mM	DMSO
126-17-0	Solasodine	413.636	10mM	DMSO
76248-63-0	Piptocarphin A	422.426	10mM	DMSO
126005-94-5	28-Deoxonimbolide	452.539	10mM	DMSO
82209-72-1	Andropanoside	496.59	10mM	DMSO
77658-38-9	Pterokaurene L3	318.45	10mM	DMSO
103476-99-9	Diacetylpiptocarphol	396.388	10mM	DMSO
152253-67-3	Mupinensisone	440.701	10mM	DMSO
776-86-3	Isoscopoletin	192.168	10mM	DMSO
165689-32-7	Torososide A	728.652	10mM	DMSO
20065-99-0	Hennadiol	442.717	10mM	DMSO
78516-69-5	Dulcioic acid	456.7	10mM	DMSO
2679-65-4	Robtein	288.252	10mM	DMSO
606-91-7	Homopterocarpin	284.307	10mM	DMSO
130395-82-3	2-Oxokolavenol	304.467	10mM	DMSO
2524-37-0	Ethyl orsellinate	196.2	10mM	DMSO
15648-86-9	Myricetin 3-O-galactoside	480.376	10mM	DMSO
381691-22-1	28-Hydroxy-3-oxoolean-12-en-29-oic acid	470.684	10mM	DMSO
79406-13-6	3α-Cinnamoyloxypterokaurene L3	464.593	10mM	DMSO
76045-49-3	Broussonin C	312.403	10mM	DMSO
1222475-77-5	3-Epimeliasenin B	468.668	10mM	DMSO
26194-57-0	Isotaxiresinol	346.374	10mM	DMSO
252333-72-5	Isotaxiresinol 9,9'-acetonide	386.438	10mM	DMSO
868409-19-2	(+)-Puerol B 2''-O-glucoside	474.457	10mM	DMSO
2150-11-0	7,3',4'-Trihydroxyflavone	270.237	10mM	DMSO
439923-16-7	Isoscabertopin	358.385	10mM	DMSO
500-66-3	Olivetol	180.244	10mM	DMSO
40951-69-7	Taxiresinol	346.374	10mM	DMSO
24224-30-4	3-Methyl-9H-carbazol-2-ol	197.233	10mM	DMSO
79406-10-3	ent-3β-Cinnamoyloxykaur-16-en-19-oic acid	448.594	10mM	DMSO
78012-28-9	1,3-Diacetylvilasinin	512.634	10mM	DMSO
480-11-5	Oroxylin A	284.263	10mM	DMSO
471271-55-3	(E)-Aldosecologanin	758.718	10mM	DMSO
3681-99-0	Puerarin	416.378	10mM	DMSO
187110-72-1	Clauszoline M	227.215	10mM	DMSO
1000676-45-8	Stilbostemin N	258.312	10mM	DMSO
139101-67-0	Tristin	260.285	10mM	DMSO
162411-67-8	Stilbostemin B	228.286	10mM	DMSO
121064-78-6	3-O-trans-p-Coumaroyltormentic acid	634.842	10mM	DMSO
123497-84-7	Demethylmurrayanine	211.216	10mM	DMSO
142846-95-5	Clausine D	279.333	10mM	DMSO
114542-44-8	Honyucitrin	406.471	10mM	DMSO
22338-69-8	Grandifloric acid	318.45	10mM	DMSO
35030-38-7	ent-17-Hydroxykaur-15-en-19-oic acid	318.45	10mM	DMSO
1309362-77-3	Cerberic acid B	210.183	10mM	DMSO

20230-41-5	Dregeoside Aa1	939.133	10mM	DMSO
6730-83-2	Kaurenoic acid	302.451	10mM	DMSO
113963-39-6	Andrographidine C	460.431	10mM	DMSO
113963-41-0	Andrographidine E	490.457	10mM	DMSO
942626-75-7	5-Hydroxy-7,8,2',5'-tetramethoxyflavone 5-O-glucoside	520.483	10mM	DMSO
492-14-8	Butin	272.253	10mM	DMSO
552-58-9	Eriodictyol	288.252	10mM	DMSO
182261-94-5	Clausine I	241.242	10mM	DMSO
139115-59-6	Isomurralonginol acetate	302.322	10mM	DMSO
160498-02-2	Bisandrographolide C	664.868	10mM	DMSO
642-18-2	Alstonine	348.395	10mM	DMSO
97169-44-3	6-Epi-8-O-acetylharpagide	406.382	10mM	DMSO
86362-16-5	6-Epiharpagide	364.345	10mM	DMSO
18449-41-7	Madecassic acid	504.699	10mM	DMSO
642-17-1	Akuammigine	352.427	10mM	DMSO
4184-34-3	Mangiferolic acid	456.7	10mM	DMSO
802909-72-4	Dehydro-δ-tocopherol	400.637	10mM	DMSO
38927-54-7	Isodeoxyelephantopin	344.358	10mM	DMSO
7560-49-8	Methyl 3,4,5-trimethoxycinnamate	252.263	10mM	DMSO
82464-35-5	3-Oxotirucalla-7,24-dien-21-oic acid	454.684	10mM	DMSO
80396-57-2	Protoplumericin A	778.707	10mM	DMSO
116499-73-1	9α,13α-Epidioxyabiet-8(14)-en-18-oic acid	334.45	10mM	DMSO
349534-73-2	Dodonolide	312.403	10mM	DMSO
79406-11-4	3α-Angeloyloxypterokaurene L3	416.55	10mM	DMSO
24694-79-9	Uvedalin	448.463	10mM	DMSO
349112-30-7	Cryptomeridiol 11-rhamnoside	386.523	10mM	DMSO
111035-65-5	Annonacin	596.878	10mM	DMSO
17297-56-2	Biorobin	594.518	10mM	DMSO
34020-07-0	Vinorine	334.412	10mM	DMSO
24694-80-2	Chlorouvedalin	484.924	10mM	DMSO
70-70-2	1-(4-Hydroxyphenyl)propan-1-one	150.174	10mM	DMSO
98665-66-8	Dregeoside Ga1	941.149	10mM	DMSO
66777-70-6	6,8-Diprenylorobol	422.47	10mM	DMSO
1246926-08-8	5,7,4'-Trihydroxy-3,6-dimethoxy-3',5'-diprenylflavone	466.523	10mM	DMSO
10163-83-4	Drevogenin A	490.629	10mM	DMSO
62623-86-3	11S,12-Dihydroxyspirovetiv-1(10)-en-2-one	252.349	10mM	DMSO
41997-41-5	Dihydropashanone	302.322	10mM	DMSO
64121-98-8	Di-O-methylcrenatin	346.33	10mM	DMSO
956384-55-7	Demethylsonchifolin	360.401	10mM	DMSO
74690-89-4	Vibsanin C	416.55	10mM	DMSO
133005-15-9	13-Epijhanol	306.483	10mM	DMSO
221466-41-7	2-Oxokolavelool	304.467	10mM	DMSO
5132-66-1	27-Hydroxymangiferonic acid	470.684	10mM	DMSO
644-06-4	Precocene II	220.264	10mM	DMSO
74474-76-3	Casegravol	276.285	10mM	DMSO
41682-30-8	8-Acetoxypentadeca-1,9Z-diene-4,6-diyn-3-ol	274.355	10mM	DMSO
17983-82-3	27-Hydroxymangiferolic acid	472.7	10mM	DMSO
6880-91-7	Dihydrochelerythrine	349.38	10mM	DMSO
232266-08-9	23-Hydroxymangiferonic acid	470.684	10mM	DMSO
1318158-89-2	Cardenolide B-1	532.666	10mM	DMSO
35109-93-4	Adynerin	516.666	10mM	DMSO
52628-62-3	Dehydroadynerigenin digitaloside	530.65	10mM	DMSO
864719-19-7	Sequosempervirin D	356.412	10mM	DMSO
62596-34-3	Cyclomorusin	418.439	10mM	DMSO
221466-42-8	2β-Hydroxykolavelool	306.483	10mM	DMSO
62574-30-5	11R,12-Dihydroxyspirovetiv-1(10)-en-2-one	252.349	10mM	DMSO
710952-13-9	5,8,4'-Trihydroxy-7-methoxyflavone 8-O-glucoside	462.403	10mM	DMSO
5835-26-7	Isopimaric acid	302.451	10mM	DMSO

220328-03-0	3,11,12-Trihydroxyspirovetiv-1(10)-en-2-one	268.349	10mM	DMSO
220328-04-1	3,11,12-Trihydroxyspirovetiv-1(10)-en-2-one	268.349	10mM	DMSO
219298-74-5	2-Hydroxy-3,4,5,6-tetramethoxychalcone	344.358	10mM	DMSO
4431-42-9	Isopedicin	330.332	10mM	DMSO
491-80-5	Biochanin A	284.263	10mM	DMSO
122-69-0	Cinnamyl cinnamate	264.318	10mM	DMSO
17303-67-2	Goniothalamin	200.233	10mM	DMSO
87402-88-8	Denudatin B	356.412	10mM	DMSO
194613-74-6	21αH-24-Norhopa-4(23),22(29)-diene-3β,6β-diol	426.674	10mM	DMSO
No	5-(1-Hydroxyethyl)-2,4-dimethoxytetrahydrofuran-3-ol	192.21	10mM	DMSO
No	17-Epidregeoside Aa1	939.133	10mM	DMSO
82209-76-5	Andrographoside	512.59	10mM	DMSO
18194-29-1	Sequirin C	302.322	10mM	DMSO
7288-11-1	Agatharesinol	286.322	10mM	DMSO
800389-33-7	Agatharesinol acetonide	326.386	10mM	DMSO
163634-05-7	Evofolin C	218.292	10mM	DMSO
38230-99-8	Chloroenhydrin	500.923	10mM	DMSO
36062-05-2	Hexahydrocurcumin	374.428	10mM	DMSO
138870-96-9	5''-Methoxyhexahydrocurcumin	404.454	10mM	DMSO
36062-04-1	Tetrahydrocurcumin	372.412	10mM	DMSO
120-05-8	Sulfuretin	270.237	10mM	DMSO
240122-32-1	Griffithinam	295.289	10mM	DMSO
96405-62-8	Goniotriol	250.247	10mM	DMSO
81122-95-4	(Z)-Lachnophyllum lactone	162.185	10mM	DMSO
16830-15-2	Asiaticoside	959.122	10mM	DMSO
79406-09-0	ent-3β-Tigloyloxykaur-16-en-19-oic acid	400.551	10mM	DMSO
74635-61-3	ent-3β-Angeloyloxykaur-16-en-19-oic acid	400.551	10mM	DMSO
129350-09-0	O-Geranylconiferyl alcohol	316.435	10mM	DMSO
75513-81-4	Cedrin	334.278	10mM	DMSO
77836-86-3	Nelumol A	346.461	10mM	DMSO
41442-57-3	5-Heptadecylresorcinol	348.563	10mM	DMSO
106894-43-3	5-Allyl-3-methoxy-6-methyl-7-(3,4,5-trimethoxyphenyl)bicy	386.438	10mM	DMSO
111843-10-8	Hancinone C	400.465	10mM	DMSO
95839-45-5	Hedyotisol B	810.837	10mM	DMSO
17020-27-8	24-Methylenecycloartane-3β,26-diol	456.743	10mM	DMSO
174423-30-4	Sanggenol A	424.486	10mM	DMSO
57726-26-8	4-(Ethoxymethyl)phenol	152.19	10mM	DMSO
114027-39-3	Humantenidine	342.389	10mM	DMSO
32811-40-8	Coniferyl alcohol	180.2	10mM	DMSO
133882-79-8	Eicosyl ferulate	474.716	10mM	DMSO
No	17-Epidrevogenin A	490.629	10mM	DMSO
18411-75-1	Hautriwaic acid	332.434	10mM	DMSO
569-92-6	Rhamnocitrin	300.263	10mM	DMSO
178764-92-6	Agrocybenine	206.284	10mM	DMSO
104021-39-8	8α-Methacryloyloxybalchanin	332.391	10mM	DMSO
129145-51-3	Gancaonin M	352.38	10mM	DMSO
6874-98-2	Vellosimine	292.375	10mM	DMSO
56407-87-5	Tetrahydrolachnophyllum lactone	166.217	10mM	DMSO
5273-85-8	Isoelemicin	208.254	10mM	DMSO
83-95-4	Skimmianine	259.257	10mM	DMSO
1195760-68-9	11-Hydroxygelsenicine	342.389	10mM	DMSO
2671-32-1	Picraline	410.463	10mM	DMSO
4030-51-7	Cassyfiline	341.358	10mM	DMSO
98665-65-7	Dregeoside Da1	814.996	10mM	DMSO
482-01-9	Homoferreirin	316.305	10mM	DMSO
1334309-44-2	3'-Geranyl-3-prenyl-2',4',5,7-tetrahydroxyflavone	490.587	10mM	DMSO
1351931-30-0	Sanggenol P	492.603	10mM	DMSO
121747-90-8	Isochandalone	404.455	10mM	DMSO

137809-97-3	2,3-Di(3',4'-methylenedioxybenzyl)-2-buten-4-olide	352.337	10mM	DMSO
13878-92-7	Isomangiferolic acid	456.7	10mM	DMSO
18465-71-9	2-C-Methyl-D-erythrono-1,4-lactone	132.115	10mM	DMSO
32885-81-7	Lasiodiplodin	292.37	10mM	DMSO
125002-91-7	8-Prenylluteone	422.47	10mM	DMSO
1897-26-3	Akuammiline	394.464	10mM	DMSO
5890-18-6	Laurolitsine	313.348	10mM	DMSO
27270-89-9	Phyllostine	154.12	10mM	DMSO
917-13-5	Enniatin B	639.82	10mM	DMSO
1197-09-7	3',4'-Dihydroxyacetophenone	152.147	10mM	DMSO
73483-88-2	Tetrachyrin	300.435	10mM	DMSO
864719-17-5	Sequosempervirin B	316.348	10mM	DMSO
3466-23-7	Dehydrodeguelin	392.401	10mM	DMSO
77394-27-5	Sesartemin	430.448	10mM	DMSO
28978-03-2	Persicoside	478.446	10mM	DMSO
1264694-96-3	Metasequirin D	346.374	10mM	DMSO
1188932-15-1	Alstoyunine E	350.411	10mM	DMSO
1212148-58-7	Secodihydro-hydramicromelin B	326.299	10mM	DMSO
31076-39-8	Cedeodarin	318.278	10mM	DMSO
480-33-1	Mellein	178.185	10mM	DMSO
65408-91-5	Altholactone	232.232	10mM	DMSO
4449-55-2	Warangalone	404.455	10mM	DMSO
482-53-1	Osajin	404.455	10mM	DMSO
911004-72-3	Furowanin A	438.47	10mM	DMSO
53734-74-0	Neorauflavane	354.396	10mM	DMSO
123316-64-3	2'-O-Methylhelichrysetin	300.306	10mM	DMSO
1309478-07-6	Wittifuran X	272.253	10mM	DMSO
156-38-7	4-Hydroxyphenylacetic acid	152.147	10mM	DMSO
88664-09-9	14-Deoxycoleon U	330.418	10mM	DMSO
146-48-5	Yohimbine	354.443	10mM	DMSO
28189-90-4	Sinensin	450.393	10mM	DMSO
17811-32-4	3-Deoxyzinnolide	262.301	10mM	DMSO
1083195-05-4	1,7-Bis(4-hydroxyphenyl)hept-6-en-3-ol	298.376	10mM	DMSO
129214-59-1	15-Dihydroepioxylubimin	254.365	10mM	DMSO
167958-89-6	Stigmasta-4,22-diene-3β,6β-diol	428.69	10mM	DMSO
30368-42-4	Dalbergioidin	288.252	10mM	DMSO
2030-53-7	Aporheine	279.333	10mM	DMSO
150226-21-4	12-Deoxo-12α-acetoxyelliptone	396.39	10mM	DMSO
522-17-8	Deguelin	394.417	10mM	DMSO
51225-28-6	6,8-Diprenylgenistein	406.471	10mM	DMSO
76-80-2	Tephrosin	410.417	10mM	DMSO
120278-25-3	4-Hydroxysapriparaquinone	330.418	10mM	DMSO
113900-75-7	Koumine N-oxide	322.401	10mM	DMSO
20958-18-3	Dihydroisotanshinone I	278.302	10mM	DMSO
15345-89-8	5,6-Dehydrokawain	228.243	10mM	DMSO
24512-62-7	Gardenoside	404.366	10mM	DMSO
85769-33-1	Alstolenine	546.611	10mM	DMSO
135905-53-2	Lupinol C	370.353	10mM	DMSO
1206734-95-3	2''-Acetylastragalin	490.414	10mM	DMSO
6880-54-2	Norfluorocurarine	292.375	10mM	DMSO
2447-70-3	Pseudoakuammigine	366.453	10mM	DMSO
240122-30-9	Griffithazanone A	257.241	10mM	DMSO
122590-03-8	11-Hydroxyrankinidine	356.416	10mM	DMSO
552-59-0	Prunetin	284.263	10mM	DMSO
370102-93-5	(-)-Variabilin	300.306	10mM	DMSO
13401-40-6	Phaseollin	322.355	10mM	DMSO
17990-42-0	Oleanonic acid	454.684	10mM	DMSO
511-05-7	Sugiol	300.435	10mM	DMSO

2466-42-4	Neolitsine	323.343	10mM	DMSO
116064-76-7	Piperolactam C	309.316	10mM	DMSO
51838-83-6	Allamandicin	308.283	10mM	DMSO
144429-71-0	Goniodiol 8-acetate	276.285	10mM	DMSO
118-71-8	Maltol	126.11	10mM	DMSO
58124-18-8	2-Hydroxynaringenin	288.252	10mM	DMSO
250691-57-7	Salvisyrianone	312.403	10mM	DMSO
83348-22-5	10-O-Caffeoyl-6-epiferetoside	566.508	10mM	DMSO
63786-17-2	12α-Hydroxygrandiflorenic acid	316.435	10mM	DMSO
32262-18-3	4-Methoxy-N-methyl-2-quinolone	189.211	10mM	DMSO
124868-11-7	Isoaltholactone	232.232	10mM	DMSO
1038753-13-7	4'-O-Methyllicoflavanone	354.396	10mM	DMSO
77263-06-0	Erythrabyssin II	392.487	10mM	DMSO
1246926-09-9	5'-Prenylaliarin	484.538	10mM	DMSO
22697-65-0	5,7,4'-Trihydroxy-3,6-dimethoxyflavone	330.289	10mM	DMSO
75679-58-2	2',4'-Dihydroxy-4,6'-dimethoxydihydrochalcone	302.322	10mM	DMSO
487-06-9	Citropten	206.195	10mM	DMSO
87585-32-8	(+)-Lyoniresinol 9'-O-glucoside	582.594	10mM	DMSO
165459-53-0	16-Hydroxy-2-oxocleroda-3,13-dien-15,16-olide	332.434	10mM	DMSO
87440-56-0	Glepidotin B	340.37	10mM	DMSO
521-51-7	Pedicin	330.332	10mM	DMSO
No	Dodonaflavonol	512.548	10mM	DMSO
92280-12-1	Sanggenon N	422.47	10mM	DMSO
832-58-6	2',4',6'-Trimethoxyacetophenone	210.226	10mM	DMSO
53734-75-1	Neorauflavene	352.38	10mM	DMSO
126737-42-6	Acetylsventenic acid	360.487	10mM	DMSO
82375-30-2	Humantenirine	370.442	10mM	DMSO
23811-50-9	Trichokaurin	434.523	10mM	DMSO
22149-65-1	Hopane-3β,22-diol	444.733	10mM	DMSO
19914-20-6	Enniatin B1	653.847	10mM	DMSO
97399-90-1	Aristolactam AIa	281.263	10mM	DMSO
117469-56-4	Prionitin	310.43	10mM	DMSO
125180-42-9	N-Methoxyanhydrovobasinediol	338.443	10mM	DMSO
1017233-48-5	6α,16,18-Trihydroxycleroda-3,13-dien-15,16-olide	350.449	10mM	DMSO
119309-02-3	Atalantoflavone	336.338	10mM	DMSO
161099-42-9	Bidwillol A	338.397	10mM	DMSO
926010-24-4	Dayecrystal A	320.466	10mM	DMSO
20784-50-3	Isobavachalcone	324.37	10mM	DMSO
4657-58-3	Cycloartanol	428.733	10mM	DMSO
No	Oxyperaksine	364.48	10mM	DMSO
959421-20-6	5,7,4'-Trihydroxy-3,6-dimethoxy-3'-prenylflavone	398.406	10mM	DMSO
118-41-2	Eudesmic acid	212.199	10mM	DMSO
59-02-9	α-Tocopherol	430.706	10mM	DMSO
72458-85-6	11-Hydroxytephrosin	426.416	10mM	DMSO
1438-62-6	13-Epimanool	290.483	10mM	DMSO
122590-04-9	11-Hydroxyhumantenine	370.442	10mM	DMSO
28342-33-8	Oxychelerythrine	363.363	10mM	DMSO
20186-22-5	Pisatin	314.289	10mM	DMSO
1092952-62-9	7,2',4'-Trihydroxy-5-methoxy-3-phenylcoumarin	300.263	10mM	DMSO
210537-04-5	1,11b-Dihydro-11b-hydroxymedicarpin	288.295	10mM	DMSO
68401-05-8	Kuwanon E	424.486	10mM	DMSO
210537-05-6	1,11b-Dihydro-11b-hydroxymaackiain	302.279	10mM	DMSO
124096-81-7	16-Epikoumidine	294.391	10mM	DMSO
27510-33-4	O-Methylpallidine	341.401	10mM	DMSO
6812-87-9	Royleanone	316.435	10mM	DMSO
1372527-39-3	Dodoviscin H	454.512	10mM	DMSO
84294-77-9	Aliarin	416.421	10mM	DMSO
1219603-97-0	4,5-Diepipsidial A	474.588	10mM	DMSO

5876-17-5	Haplopine	245.231	10mM	DMSO
3690-05-9	p-Coumaryl alcohol	150.174	10mM	DMSO
126778-79-8	Sventenic acid	318.45	10mM	DMSO
53948-07-5	Aristolactam AII	265.263	10mM	DMSO
145643-96-5	Cyclocommunol	352.337	10mM	DMSO
488-17-5	3-Methylcatechol	124.137	10mM	DMSO
1372527-40-6	Dodoviscin I	386.395	10mM	DMSO
1372527-25-7	Dodoviscin A	500.538	10mM	DMSO
71850-15-2	Camaldulenic acid	470.684	10mM	DMSO
865187-17-3	Gelsempervine A	382.453	10mM	DMSO
139954-00-0	Palbinone	358.471	10mM	DMSO
136685-37-5	9-Deoxygoniopypyrone	234.248	10mM	DMSO
20486-27-5	Procumbide	362.329	10mM	DMSO
142763-37-9	3,4-Didehydrosapriparaquione	312.403	10mM	DMSO
80454-42-8	Paeoniflorigenone	318.321	10mM	DMSO
69651-80-5	Hesperetin 5-O-glucoside	464.419	10mM	DMSO
74515-47-2	Cristacarpin	354.396	10mM	DMSO
6935-99-5	Obtucarbamate A	238.24	10mM	DMSO
20913-18-2	Obtucarbamate B	238.24	10mM	DMSO
482-45-1	Isoimperatorin	270.28	10mM	DMSO
771493-42-6	6α-Hydroxycleroda-3,13-dien-16,15-olid-18-oic acid	348.433	10mM	DMSO
61135-92-0	6a-Hydroxymedicarpin	286.279	10mM	DMSO
114027-38-2	16-Epivoacarpine	368.426	10mM	DMSO
39986-86-2	4'-Hydroxy-5,6-dehydrokawain	244.243	10mM	DMSO
96422-53-6	Goniodiol 7-acetate	276.285	10mM	DMSO
123702-94-3	Kuwanol C	422.47	10mM	DMSO
51593-96-5	Cuspidiol	236.307	10mM	DMSO
200813-31-6	16-Nor-15-oxodehydroabietic acid	300.392	10mM	DMSO
90468-72-7	Maoyerabdosin	468.537	10mM	DMSO
91913-76-7	1-Methoxyindole-3-carboxylic acid	191.183	10mM	DMSO
61218-44-8	6a-Hydroxymaackiain	300.263	10mM	DMSO
206560-99-8	7-O-Methylporiol	300.306	10mM	DMSO
63910-76-9	Panaxydiol	260.371	10mM	DMSO
479-13-0	Coumestrol	268.221	10mM	DMSO
55610-01-0	Cepharadione A	305.284	10mM	DMSO
105330-59-4	5,7,4'-Tri-O-methylcatechin	332.348	10mM	DMSO
151334-06-4	3-O-p-Coumaroyloleanolic acid	602.843	10mM	DMSO
246870-75-7	Derrisisoflavone B	422.47	10mM	DMSO
136778-40-0	Goniodiol diacetate	318.321	10mM	DMSO
129578-07-0	Goniopypyrone	250.247	10mM	DMSO
17948-42-4	Venoterpine	149.19	10mM	DMSO
96422-52-5	Goniodiol	234.248	10mM	DMSO
97534-10-6	3-O-Caffeoyloleanolic acid	618.842	10mM	DMSO
227289-51-2	(3S,7S)-5,6-Dehydro-4"-de-O-methylcentrolobine	296.36	10mM	DMSO
30536-48-2	Caulilexin C	186.21	10mM	DMSO
883859-83-4	8-Lavandulylkaempferol	422.47	10mM	DMSO
94596-28-8	Senkyunolide I	224.253	10mM	DMSO
76122-57-1	Phaseollidin hydrate	342.386	10mM	DMSO
60976-49-0	Geraniin	952.645	10mM	DMSO
98751-78-1	Paeonilactone B	196.2	10mM	DMSO
98751-77-0	Paeonilactone C	318.321	10mM	DMSO
133442-54-3	Angophorol	314.333	10mM	DMSO
27468-20-8	Deoxyneocryptotanshinone	298.376	10mM	DMSO
221002-11-5	1"-Methoxyerythrinin C	384.379	10mM	DMSO
35354-74-6	Honokiol	266.334	10mM	DMSO
98751-79-2	Paeonilactone A	198.216	10mM	DMSO
52151-92-5	Piperitol	356.369	10mM	DMSO
1916-07-0	Methyl 3,4,5-trimethoxybenzoate	226.226	10mM	DMSO

94451-48-6	Syzalterin	298.29	10mM	DMSO
80621-54-1	8-Demethylsideroxylin	298.29	10mM	DMSO
60297-37-2	Auriculasin	420.454	10mM	DMSO
59901-98-3	3-Hydroxy-8,9-methylenedioxypterocarpene	282.248	10mM	DMSO
754919-24-9	N-Methylnuciferine	310.41	10mM	DMSO
53846-50-7	8-Prenylnaringenin	340.37	10mM	DMSO
82373-94-2	2,3,5,4'-Tetrahydroxystilbene 2-O-glucoside	406.383	10mM	DMSO
6178-44-5	Ethyl 3,4,5-trimethoxybenzoate	240.252	10mM	DMSO
975-77-9	Pericyclivine	322.401	10mM	DMSO
76-98-2	Conopharyngine	398.495	10mM	DMSO
16790-93-5	19(S)-Hydroxyconopharyngine	414.495	10mM	DMSO
220736-54-9	Curlignan	360.358	10mM	DMSO
478158-77-9	Isoerysenegalensein E	422.47	10mM	DMSO
154992-17-3	Erysenegalensein E	422.47	10mM	DMSO
1151862-67-7	6-Epiharpagoside	494.488	10mM	DMSO
1072-93-1	Epigoitrin	129.18	10mM	DMSO
99217-63-7	Kushenol A	408.487	10mM	DMSO
51-34-3	Scopolamine	303.353	10mM	DMSO
4684-28-0	Norscopolamine	289.326	10mM	DMSO
18642-23-4	Psoralidin	336.338	10mM	DMSO
23313-21-5	Anthraglycoside B	432.378	10mM	DMSO
89706-39-8	7-O-Acetyl-4-O-demethylpolysyphorin	446.49	10mM	DMSO
1245636-01-4	ent-Labda-8(17),13Z-diene-15,16,19-triol 19-O-glucoside	484.623	10mM	DMSO
147663-91-0	Magnolianin	826.97	10mM	DMSO
56121-44-9	4-O-Methylhelichrysetin	300.306	10mM	DMSO
61617-29-6	Songoroside A	588.815	10mM	DMSO
118477-06-8	Cyclo(L-Phe-trans-4-hydroxy-L-Pro)	260.288	10mM	DMSO
No	2-Ethoxy-3-acetonyltaxifolin	404.367	10mM	DMSO
1044743-35-2	3'-Methylflavokawin	314.333	10mM	DMSO
59086-93-0	Dehydrotoxicarol	408.401	10mM	DMSO
174204-83-2	3-Chloro-4-hydroxypiperidin-2-one	149.576	10mM	DMSO
89020-11-1	Dregeoside A11	1101.27	10mM	DMSO
115006-86-5	Cyclo(L-Leu-trans-4-hydroxy-L-Pro)	226.272	10mM	DMSO
84873-15-4	p-Hydroxyphenethyl trans-ferulate	314.333	10mM	DMSO
480-66-0	2',4',6'-Trihydroxyacetophenone	168.147	10mM	DMSO
90536-74-6	Ethyl 2,4,6-trihydroxybenzoate	198.173	10mM	DMSO
486-84-0	Harman	182.221	10mM	DMSO
63644-71-3	γ-Methoxyisoeugenol	194.227	10mM	DMSO
No	Ethyl 9,12,13-trihydroxyoctadeca-10,15-dienoate	356.497	10mM	DMSO
186140-36-3	11α,12α-Epoxy-3β,23-dihydroxy-30-norolean-20(29)-en-28,	470.641	10mM	DMSO
6519-27-3	(16R)-E-Isositsirikine	354.443	10mM	DMSO
89199-99-5	Mulberrofuran H	442.46	10mM	DMSO
464-45-9	Borneol	154.249	10mM	DMSO
82513-70-0	10-Hydroxy-16-epiaffinine	340.416	10mM	DMSO
87562-14-9	Randaiol	242.27	10mM	DMSO
1280602-81-4	erythro-1-(4-Hydroxy-3-methoxyphenyl)propane-1,2-diol	198.216	10mM	DMSO
848031-94-7	threo-1-(4-Hydroxy-3-methoxyphenyl)propane-1,2-diol	198.216	10mM	DMSO
16049-28-8	Antirhine	296.407	10mM	DMSO
123702-97-6	Moracin O	326.343	10mM	DMSO
112448-69-8	3-Phenyl-1-(pyrrol-1-yl)propan-1-one	199.248	10mM	DMSO
448905-82-6	3-(4-Methoxyphenyl)-1-(pyrrol-1-yl)propan-1-one	229.274	10mM	DMSO
252248-89-8	1-Cinnamoylpyrrole	197.233	10mM	DMSO
72917-31-8	(Z)-Butylidenephthalide	188.222	10mM	DMSO
94596-27-7	Senkyunolide H	224.253	10mM	DMSO
87085-00-5	Mulberrofuran G	562.565	10mM	DMSO
102841-46-3	Moracin P	326.343	10mM	DMSO
26488-24-4	Cyclo(D-Phe-L-Pro)	244.289	10mM	DMSO
36357-32-1	Cyclo(L-Ala-L-Pro)	168.193	10mM	DMSO

6052-73-9	5,6-Dihydropyridin-2(1H)-one	97.1152	10mM	DMSO
19202-36-9	Hinokiflavone	538.458	10mM	DMSO
41060-15-5	Neobavaisoflavone	322.355	10mM	DMSO
19367-38-5	Methyl 4-hydroxycinnamate	178.185	10mM	DMSO
124858-37-3	5-Dehydroxyparatocarpin K	322.355	10mM	DMSO
548-19-6	Isoginkgetin	566.511	10mM	DMSO
130263-10-4	3'-Demethoxypiplartine	287.31	10mM	DMSO
514-62-5	Ferruginol	286.452	10mM	DMSO
188894-19-1	Junipediol B 8-O-glucoside	358.34	10mM	DMSO
22329-76-6	Methyl ferulate	208.211	10mM	DMSO
2316-26-9	3,4-Dimethoxycinnamic acid	208.211	10mM	DMSO
29376-68-9	Thevetiaflavone	284.263	10mM	DMSO
865887-46-3	Oleuropeic acid 8-O-glucoside	346.373	10mM	DMSO
66568-97-6	Tsugafolin	300.306	10mM	DMSO
736140-70-8	1-(4-Methoxycinnamoyl)pyrrole	227.259	10mM	DMSO
36238-67-2	Cyclo(D-Leu-L-Pro)	210.273	10mM	DMSO
126640-98-0	16-Epinormacusine B	294.391	10mM	DMSO
775351-88-7	Corylifol A	390.472	10mM	DMSO
958631-84-0	(E)-1-(4-Hydroxyphenyl)dec-1-en-3-one	246.345	10mM	DMSO
7727-79-9	Zederone	246.302	10mM	DMSO
77996-04-4	Mulberrofuran C	580.581	10mM	DMSO
72500-11-9	Methyl vanillate glucoside	344.314	10mM	DMSO
34421-19-7	2,3-Dihydrosciadopitysin	582.554	10mM	DMSO
828923-27-9	2,3-Dihydroisoginkgetin	568.527	10mM	DMSO
128261-84-7	1-(3,4-Dimethoxycinnamoyl)piperidine	275.343	10mM	DMSO
270249-38-2	2'-O-Methylkurarinone	452.539	10mM	DMSO
22767-72-2	Ethyl 3-(4-methoxyphenyl)propanoate	208.254	10mM	DMSO
20575-57-9	Calycosin	284.263	10mM	DMSO
24338-53-2	Nagilactone C	362.374	10mM	DMSO
116271-35-3	Pyrroside B	566.508	10mM	DMSO
18836-52-7	Pellitorine	223.354	10mM	DMSO
97938-30-2	Sophoraflavanone G	424.486	10mM	DMSO
272122-56-2	Decursidate	330.332	10mM	DMSO
104691-86-3	Lupiwighteone	338.354	10mM	DMSO
27127-79-3	Thevetin B	858.963	10mM	DMSO
331-39-5	Caffeic acid	180.157	10mM	DMSO
38965-51-4	Eriodictyol 7-O-glucoside	450.393	10mM	DMSO
28610-31-3	8-Prenylkaempferol	354.353	10mM	DMSO
20188-85-6	Ombuoside	638.571	10mM	DMSO
15823-04-8	Methyl 3-(4-methoxyphenyl)propanoate	194.227	10mM	DMSO
5027-30-5	Phloracetophenone 4'-O-glucoside	330.287	10mM	DMSO
67685-22-7	Anhydroglycinol	254.238	10mM	DMSO
38642-49-8	Benzoylpaeoniflorin	584.568	10mM	DMSO
572-32-7	Ayanin	344.315	10mM	DMSO
501-52-0	Hydrocinnamic acid	150.174	10mM	DMSO
23180-57-6	Paeoniflorin	480.462	10mM	DMSO
190381-82-9	Orientanol A	388.411	10mM	DMSO
514-10-3	Abietic acid	302.451	10mM	DMSO
55102-39-1	4β-Carboxy-19-nortotarol	316.435	10mM	DMSO
178765-54-3	3-Hydroxybakuchiol	272.382	10mM	DMSO
47326-53-4	Spegatrine	325.424	10mM	DMSO
489-32-7	Icariin	676.662	10mM	DMSO
475-81-0	Glaucine	355.428	10mM	DMSO
33417-17-3	3-Hydroxy-3-acetonyloxindole	205.21	10mM	DMSO
6877-32-3	Corynoxine	384.469	10mM	DMSO
93753-33-4	Magnaldehyde D	254.281	10mM	DMSO
56083-03-5	Isobavachromene	322.355	10mM	DMSO
20013-76-7	Dehydrochromolaenin	210.271	10mM	DMSO

5041-82-7	Isorhamnetin 3-O-glucoside	478.403	10mM	DMSO
93-15-2	Methyleugenol	178.228	10mM	DMSO
143601-07-4	Acuminatin	512.505	10mM	DMSO
101236-50-4	Kushenol L	440.486	10mM	DMSO
65332-45-8	Demethylvestitol	258.269	10mM	DMSO
3064-05-9	Cycloolivil	376.4	10mM	DMSO
21913-98-4	3'-Methoxydaidzein	284.263	10mM	DMSO
137787-00-9	Boeravinone E	328.273	10mM	DMSO
93673-81-5	Magnolignan A	300.349	10mM	DMSO
52438-21-8	1-Cinnamoylpyrrolidine	201.264	10mM	DMSO
2122-36-3	Apparicine	264.4	10mM	DMSO
63976-69-2	13-Oxopodocarp-8(14)-en-18-oic acid	276.371	10mM	DMSO
852385-13-8	6-Methyl-7-O-methylaromadendrin	316.31	10mM	DMSO
27483-18-7	Cyclo(D-Val-L-Pro)	196.25	10mM	DMSO
7159-86-6	3α,6β-Ditigloyloxytropan-7β-ol	337.416	10mM	DMSO
6711-69-9	Tubotaiwine	324.4	10mM	DMSO
3778-25-4	7"-O-Methylsciadopitysin	594.6	10mM	DMSO
110382-42-8	2,3-Dihydroheveaflavone	582.6	10mM	DMSO
72959-46-7	6,7-Dihydroneridienone A	328.5	10mM	DMSO
3705-26-8	Cyclo(L-Phe-L-Pro)	244.3	10mM	DMSO
6831-17-0	Aristolone	218.34	10mM	DMSO
19452-84-7	Taberpsychine	308.425	10mM	DMSO
19891-51-1	Nagilactone B	364.4	10mM	DMSO
59-48-3	Oxindole	133.15	10mM	DMSO
107633-69-2	Dehydroformouregine	337.4	10mM	DMSO
158642-42-3	Yixingensin	492.43	10mM	DMSO
3705-27-9	Cyclo(Gly-L-Pro)	154.169	10mM	DMSO
113773-90-3	Eudesm-4(15)-ene-3α,11-diol	238.4	10mM	DMSO
873999-88-3	2,3-Dihydroamentoflavone 7,4'-dimethyl ether	568.5	10mM	DMSO
109471-13-8	3'-Methyl-4-O-methylhelichrysetin	314.33	10mM	DMSO
333798-10-0	9-O-Methyl-4-hydroxyboeravinone B	342.3	10mM	DMSO
913690-46-7	1"-Hydroxyerythrinin C	370.4	10mM	DMSO
20013-75-6	Pyrocurzerenone	212.292	10mM	DMSO
125072-69-7	Epinortrachelogenin	374.4	10mM	DMSO
485811-84-5	Mirabijalone D	342.3	10mM	DMSO
16790-92-4	Crassanine	414.5	10mM	DMSO
1411629-26-9	Epiguajadial B	474.6	10mM	DMSO
67349-43-3	Pterokaurane R	322.5	10mM	DMSO
30435-26-8	Pelirine	354.5	10mM	DMSO
1207861-69-5	Gynosaponin I	769	10mM	DMSO
163060-07-9	Lup-20(29)-ene-3β,23-diol	442.7	10mM	DMSO
93697-42-8	Magnolignan C	300.4	10mM	DMSO
73069-13-3	Atractylenolide I	230.3	10mM	DMSO
15486-34-7	Kaempferol 3,7,4'-trimethyl ether	328.3	10mM	DMSO
171817-95-1	2-(1H-Indole-3-carboxamido)benzoic acid	280.283	10mM	DMSO
162229-27-8	Dorsmanin A	324.4	10mM	DMSO
160568-14-9	Zarzissine	135.1	10mM	DMSO
118525-40-9	Icaritin	368.38	10mM	DMSO
113558-15-9	Icariside II	514.527	10mM	DMSO
152464-78-3	Leachianone G	356.4	10mM	DMSO
6859-01-4	Isorhynchophylline	384.47	10mM	DMSO
41682-21-7	8-Methyleugenitol	220.2	10mM	DMSO
70872-29-6	Isoxanthohumol	354.402	10mM	DMSO
118266-99-2	Isodorsmanin A	324.4	10mM	DMSO
874303-33-0	Millewanin G	438.5	10mM	DMSO
1190070-91-7	Abiesinol F	542.5	10mM	DMSO
122855-49-6	Panaxyne	220.3	10mM	DMSO
552-66-9	Daidzin	416.38	10mM	DMSO

84414-40-4	23-Hydroxybetulin	458.7	10mM	DMSO
135626-13-0	Coccineone B	298.25	10mM	DMSO
874303-34-1	Millewanin H	438.5	10mM	DMSO
114567-34-9	Boeravinone B	312.3	10mM	DMSO
82-09-7	α-Toxicarol	410.4	10mM	DMSO
3044-60-8	Toxicarolisoflavone	410.42	10mM	DMSO
61854-37-3	Demethoxy-7-O-methylcapillarisin	300.27	10mM	DMSO
775351-91-2	Corylifol C	338.4	10mM	DMSO
207792-17-4	1,7-Bis(4-hydroxyphenyl)-3-hydroxy-1,3-heptadien-5-one	310.35	10mM	DMSO
6792-07-0	Macusine B	309.4	10mM	DMSO
66556-91-0	ent-3β-Hydroxykaur-16-en-19-oic acid	318.5	10mM	DMSO
717901-03-6	Verbenacine	318.5	10mM	DMSO
23179-78-4	Songoramine	355.5	10mM	DMSO
389572-70-7	Piperlotine A	231.3	10mM	DMSO
34981-26-5	Kurarinone	438.51	10mM	DMSO
955135-37-2	8,3'-Diprenylapigenin	406.5	10mM	DMSO
161068-53-7	Epimedokoreanin B	422.5	10mM	DMSO
1245-00-7	Sitsirikine	354.5	10mM	DMSO
101046-79-1	Larixinol	542.5	10mM	DMSO
52328-96-8	Bisdemethoxycurcumin	308.33	10mM	DMSO
886989-88-4	Piperlotine C	291.4	10mM	DMSO
2068/2/2	3,7-Di-O-methylquercetin	330.3	10mM	DMSO
13323-48-3	Heliangin	362.4	10mM	DMSO
50333-13-6	N-Methylflindersine	241.3	10mM	DMSO
485-72-3	Formononetin	268.26	10mM	DMSO
21293-29-8	Abscisic acid	264.32	10mM	DMSO
654663-85-1	Acantrifoic acid A	544.7	10mM	DMSO
20196-89-8	Kaempferol 7-O-rhamnoside	432.4	10mM	DMSO
518-17-2	Evodiamine	303.36	10mM	DMSO
20493-56-5	Curzerenone	230.3	10mM	DMSO
99633-05-3	14-Benzoylneoline	541.7	10mM	DMSO
958296-13-4	Piperlotine D	291.4	10mM	DMSO
807372-38-9	4'-Demethoxypiperlotine C	261.321	10mM	DMSO
55395-07-8	Baohuoside II	500.5	10mM	DMSO
1207671-28-0	3,2'-Epilarixinol	542.5	10mM	DMSO
332371-82-1	1,7-Bis(4-hydroxyphenyl)hepta-4,6-dien-3-one	294.34	10mM	DMSO
2450-53-5	3,5-Di-O-caffeoylquinic acid	516.45	10mM	DMSO
1344876-77-2	1-Cinnamoyl-3-hydroxypyrrolidine	217.3	10mM	DMSO
574-84-5	Fraxetin	208.17	10mM	DMSO
135293-13-9	2''-O-Rhamnosylicariside II	660.7	10mM	DMSO
187539-57-7	Ethyl rutinoside	354.35	10mM	DMSO
1309920-99-7	15-Methoxymkapwanin	360.5	10mM	DMSO
40918-90-9	3,4-Dimethoxycinnamyl alcohol	194.2	10mM	DMSO
466-26-2	Neoline	337.57	10mM	DMSO
6902-91-6	Germacrone	218.33	10mM	DMSO
163815-35-8	7β-Hydroxyrutaecarpine	303.3	10mM	DMSO
87064-61-7	Euphohelioscopin A	498.66	10mM	DMSO
156974-99-1	Isosalicifolin	356.4	10mM	DMSO
56973-51-4	Alnusdiol	314.4	10mM	DMSO
549-32-6	Reynoutrin	434.4	10mM	DMSO
133568-79-3	2-Ethyl-2,6,6-trimethylpiperidin-4-one	169.3	10mM	DMSO
509-24-0	Songorine	357.49	10mM	DMSO
5041-67-8	Juglanin	418.4	10mM	DMSO
22368-21-4	Eupatilin	344.31	10mM	DMSO
14103-09-4	1-Hydroxy-2,3,4,7-tetramethoxyxanthone	332.3	10mM	DMSO
262272-76-4	3α-Acetoxy-20-oxo-29-norlupane-23,28-dioic acid	530.7	10mM	DMSO
4931-66-2	Methyl L-pyroglutamate	143.14	10mM	DMSO
21422-04-8	Demethylsuberosin	230.3	10mM	DMSO

639-36-1	Akuammidine	352.434	10mM	DMSO
132362-42-6	10-Hydroxyneoline	453.6	10mM	DMSO
180164-14-1	Megastigm-7-ene-3,4,6,9-tetrol	244.3	10mM	DMSO
116424-69-2	Fargesone A	372.4	10mM	DMSO
83-46-5	β-Sitosterol	414.69	10mM	DMSO
163434-73-9	2",4"-Di-O-(E-p-coumaroyl)afzelin	724.7	10mM	DMSO
74730-10-2	Pterolactone A	260.289	10mM	DMSO
79120-40-4	Hannokinol	316.4	10mM	DMSO
28178-92-9	Futoquinol	354.4	10mM	DMSO
254886-77-6	Kushenol X	440.5	10mM	DMSO
99340-07-5	Kadsurin A	372.4	10mM	DMSO
870480-56-1	Bis-5,5-nortrachelogenin	746.8	10mM	DMSO
868405-37-2	Protosappanin A dimethyl acetal	318.32	10mM	DMSO
99217-75-1	Mulberrofuran G pentaacetate	772.8	10mM	DMSO
171438-55-4	Dammarenediol II 3-O-caffeate	606.9	10mM	DMSO
4046-02-0	Ethyl ferulate	222.24	10mM	DMSO
90411-12-4	Neochamaejasmin B	542.5	10mM	DMSO
87355-32-6	Lupeolic acid	456.7	10mM	DMSO
67879-81-6	Glycosolone	456.7	10mM	DMSO
288259-72-3	Denudanolide A	356.4	10mM	DMSO
93859-63-3	Isochamaejasmin	542.5	10mM	DMSO
2034-69-7	Daphnoretin	352.3	10mM	DMSO
455255-15-9	Isovouacapenol C	438.56	10mM	DMSO
6379-72-2	trans-Methylisoeugenol	178.23	10mM	DMSO
32507-66-7	Isorhapontigenin	258.3	10mM	DMSO
116424-70-5	Fargesone B	372.41	10mM	DMSO
76376-43-7	Euphorbia factor L1	552.7	10mM	DMSO
67214-05-5	2"-O-Coumaroyljuglanin	564.5	10mM	DMSO
16805-10-0	Mearnsetin	332.26	10mM	DMSO
120028-43-5	Edgeworin	322.272	10mM	DMSO
26509-45-5	Methyleugenolglycol	212.24	10mM	DMSO
57475-62-4	16-Deoxysaikogenin F	456.711	10mM	DMSO
99-24-1	Methyl gallate	184.15	10mM	DMSO
526-06-7	Eudesmin	386.44	10mM	DMSO
76843-23-7	Przewaquinone A	310.4	10mM	DMSO
112515-37-4	Neolinine	423.54	10mM	DMSO
1521-41-1	3,4-Dimethoxybenzamide	181.19	10mM	DMSO
152784-32-2	Puerol A	298.3	10mM	DMSO
150033-85-5	Cycloshizukaol A	548.62	10mM	DMSO
39945-41-0	Norglaucine hydrochloride	377.86	10mM	DMSO
22804-49-5	1-Hydroxy-2,3,5-trimethoxyxanthone	302.28	10mM	DMSO
78749-47-0	Shizukanolide C	246.3	10mM	DMSO
327-97-9	Chlorogenic acid	354.31	10mM	DMSO
151200-49-6	Borapetoside E	536.57	10mM	DMSO
1269839-26-0	1-(3,4-Dihydroxyphenyl)-7-(4-hydroxyphenyl)heptane-3,5-d	416.46	10mM	DMSO
16265-56-8	6-Deoxyjacareubin	310.3	10mM	DMSO
24211-30-1	Farrerol	300.3	10mM	DMSO
480-64-8	Orsellinic acid	168.15	10mM	DMSO
27770-13-4	5-Hydroxy-1-methoxyxanthone	242.23	10mM	DMSO
39012-04-9	Epimedoside A	662.7	10mM	DMSO
305-01-1	Esculetin	178.14	10mM	DMSO
151200-50-9	Borapetoside F	534.55	10mM	DMSO
88668-99-9	8-Epideoxyloganic acid	360.36	10mM	DMSO
885044-12-2	Farrerol 7-O-glucoside	462.45	10mM	DMSO
54854-91-0	Sanshodiol	358.39	10mM	DMSO
57625-31-7	Piperenone	388.454	10mM	DMSO
84-99-1	Xanthoxyletin	258.269	10mM	DMSO
120211-98-5	1β-Hydroxyeuscaphic acid	504.7	10mM	DMSO

486-21-5	Isofraxidin	222.19	10mM	DMSO
122537-59-1	Gelomulide A	374.471	10mM	DMSO
62163-24-0	Mirandin B	386.438	10mM	DMSO
916236-79-8	Gopherenediol	306.483	10mM	DMSO
16981-20-7	Mesuol	392.451	10mM	DMSO
218916-52-0	Euphorbia factor L3	522.63	10mM	DMSO
67920-48-3	1,6-Dihydro-4,7'-epoxy-1-methoxy-3',4'-methylenedioxy-6-c	340.37	10mM	DMSO
68353-24-2	1-Methyl-2-nonylquinolin-4(1H)-one	285.424	10mM	DMSO
498-02-2	Acetovanillone	166.17	10mM	DMSO
1269839-24-8	5-Hydroxy-1,7-bis(4-hydroxyphenyl)heptan-3-yl acetate	358.428	10mM	DMSO
1098-92-6	Kaempferol 5,7,4'-trimethyl ether	328.316	10mM	DMSO
62820-11-5	Jolkinol A	480.6	10mM	DMSO
118-10-5	Cinchonine	294.39	10mM	DMSO
130-95-0	Quinine	324.42	10mM	DMSO
122537-60-4	Gelomulide B	388.454	10mM	DMSO
128988-55-6	Icariside I hydrate	548.5	10mM	DMSO
438536-34-6	3(20)-Phytene-1,2-diol	312.53	10mM	DMSO
464-85-7	Quinamine	312.406	10mM	DMSO
59443-02-6	1-Methyl-2-undecylquinolin-4(1H)-one	313.477	10mM	DMSO
15266-35-0	Dihydroevocarpine	341.5	10mM	DMSO
244277-75-6	3,5,7,15-Tetraacetoxy-9-nicotinoyloxy-6(17),11-jatrophadie	641.705	10mM	DMSO
104901-05-5	Borapetoside B	552.568	10mM	DMSO
269739-78-8	Aglaxiflorin D	646.727	10mM	DMSO
119642-82-9	Yucalexin P-17	318.45	10mM	DMSO
28808-62-0	Fraxinellone	232.28	10mM	DMSO
40421-52-1	erythro-1-Phenylpropane-1,2-diol	152.19	10mM	DMSO
1251830-57-5	1,7-Bis(4-hydroxyphenyl)hept-6-en-3-one	296.36	10mM	DMSO
174819-51-3	Rabdoketone B	164.201	10mM	DMSO
111830-77-4	10-O-Methylprotosappanin B	318.321	10mM	DMSO
99-20-7	Trehalose	342.297	10mM	DMSO
1092103-22-4	ent-11β-Hydroxyatis-16-ene-3,14-dione	316.435	10mM	DMSO
1221178-16-0	Dehydroborapetoside B	550.552	10mM	DMSO
125292-97-9	Wallichinine	370.439	10mM	DMSO
140669-89-2	Kadsurenin D	356.412	10mM	DMSO
501-96-2	(-)-Rhododendrol	166.2	10mM	DMSO
177468-85-8	Aglain C	630.727	10mM	DMSO
57430-03-2	1,6-Dihydro-4,7'-epoxy-1-methoxy-3',4'-methylenedioxy-6-c	340.37	10mM	DMSO
112523-91-8	ent-16α,17-Dihydroxyatisan-3-one	320.466	10mM	DMSO
61240-34-4	Denudadione C	340.37	10mM	DMSO
69573-60-0	Octadecyl caffeate	432.636	10mM	DMSO
137018-33-8	3'',4''-Di-O-acetyl-2'',6''-di-O-p-coumaroylastragalin	824.736	10mM	DMSO
13018-10-5	Torilin	824.736	10mM	DMSO
151200-48-5	Borapetoside D	698.709	10mM	DMSO
74805-92-8	Methylophiopogonanone A	342.34	10mM	DMSO
120061-96-3	Shizukanolide F	262.301	10mM	DMSO
74805-91-7	Methylophiopogonanone B	328.36	10mM	DMSO
1228964-10-0	12-Demethylneocaesalpin F	470.555	10mM	DMSO
1000995-47-0	Chloramultilide B	734.742	10mM	DMSO
263249-77-0	Perilloxin	274.312	10mM	DMSO
14017-71-1	(-)-Praeruptorin A	386.395	10mM	DMSO
147976-35-0	Isokadsurenin D	356.412	10mM	DMSO
83708-70-7	Dihydrosesamin	356.369	10mM	DMSO
7047-54-3	Corymbol	322.482	10mM	DMSO
80510-09-4	N-cis-Feruloyltyramine	313.4	10mM	DMSO
1000995-48-1	Chloramultilide C	734.7	10mM	DMSO
489-86-1	Guaiol	222.37	10mM	DMSO
477336-75-7	Ophiopogonanone C	356.33	10mM	DMSO
65907-75-7	Danshenxinkun A	296.32	10mM	DMSO

1207185-03-2	Sarcandrolide D	678.72	10mM	DMSO
18085-97-7	Jaceosidin	330.29	10mM	DMSO
485-49-4	(+)-Bicuculline	367.35	10mM	DMSO
86639-52-3	7-Ethyl-10-hydroxycamptothecin	397.45	10mM	DMSO
349545-02-4	3'',4''-Di-O-acetyl-2'',6''-di-O-p-coumaroylastragalin	824.74	10mM	DMSO
464-86-8	Conquinamine	312.41	10mM	DMSO
6451-73-6	Scoulerine	327.37	10mM	DMSO
27208-80-6	Polydatin	390.4	10mM	DMSO
921211-29-2	ent-16-Kaurene-3β,15β,18-triol	320.47	10mM	DMSO
588706-66-5	Ophiopogonanone E	360.36	10mM	DMSO
115783-44-3	ent-Atisane-3β,16α,17-triol	322.48	10mM	DMSO
133369-42-3	4-Hydroxy-11,12,13-trinor-5-eudesmen-7-one	194.27	10mM	DMSO
24405-57-0	β-Rotunol	234.33	10mM	DMSO
112500-90-0	6-Aldehydo-isoophiopogonone A	354.31	10mM	DMSO
75239-63-3	Ophiopogonanone A	328.32	10mM	DMSO
588706-67-6	Ophiopogonanone F	374.38	10mM	DMSO
945619-74-9	Ophiopogonin D	855.02	10mM	DMSO
212201-12-2	5,7-Dihydroxy-3-(4-hydroxy-3,5-dimethoxybenzyl)-6,8-dim	374.39	10mM	DMSO
79082-64-7	(-)-Corlumine	383.39	10mM	DMSO
76994-07-5	Euphroside	376.36	10mM	DMSO
149180-48-3	3-(2,4-Dihydroxybenzyl)-5-hydroxy-7,8-dimethoxy-6-methy	360.36	10mM	DMSO
24778-48-1	β-Peltoboykinolic acid	456.71	10mM	DMSO
102115-79-7	Pseudoprotodioscin	1031.18	10mM	DMSO
177262-32-7	Aglain B	630.73	10mM	DMSO
82958-44-9	11-O-Galloylbergenin	480.38	10mM	DMSO
105108-20-1	Ligucyperonol	234.33	10mM	DMSO
149998-38-9	Gelomuloside A	622.57	10mM	DMSO
149998-39-0	Gelomuloside B	608.54	10mM	DMSO
1005212-02-1	Gelomulide N	432.51	10mM	DMSO
75567-38-3	20-Deoxyingenol 3-angelate	414.54	10mM	DMSO
4970-26-7	(-)-Praeruptorin B	426.465	10mM	DMSO
82958-45-0	4-O-Galloylbergenin	480.378	10mM	DMSO
1011762-93-8	Longipedlactone J	536.665	10mM	DMSO
3520-14-7	Tetrahydropalmatine	355.41	10mM	DMSO
19057-60-4	Dioscin	869.04	10mM	DMSO
19083-00-2	Gracillin	885.04	10mM	DMSO
666250-52-8	2',4'-Dihydroxy-3,7':4,8'-diepoxylign-7-ene	298.338	10mM	DMSO
645414-25-1	Acantrifoside E	356.37	10mM	DMSO
85022-66-8	Shikonofuran A	316.35	10mM	DMSO
81969-41-7	Splendoside	406.38	10mM	DMSO
541-15-1	L-Carnitine	161.201	10mM	DMSO
70-18-8	Glutathione	307.32	10mM	DMSO
53-43-0	Dehydroepiandrosterone	288.424	10mM	DMSO
73-31-4	Melatonin	232.28	10mM	DMSO
887147-75-3	1α-Hydroxytorilin	392.492	10mM	DMSO
127179-70-8	4-O-Demethylkadsurenin D	342.39	10mM	DMSO
89104-59-6	4-O-Demethylisokadsurenin D	342.39	10mM	DMSO
75853-60-0	Dehydroevodiamine hydrochloride	337.8	10mM	DMSO
113626-22-5	Metasequoic acid A	302.458	10mM	DMSO
484-29-7	Dictamnine	199.21	10mM	DMSO
2255-50-7	Robustine	215.205	10mM	DMSO
62517-34-4	Ethyl p-hydroxyphenyllactate	210.23	10mM	DMSO
19427-82-8	Selinidin	328.36	10mM	DMSO
1220707-33-4	2''-O-Acetylsprengerinin C	897.05	10mM	DMSO
19057-67-1	Prosapogenin A	740.92	10mM	DMSO
57498-69-8	Angeflorin	476.43	10mM	DMSO
88861-91-0	Sprengerinin C	855.02	10mM	DMSO
88866-99-3	Sprengerinin A	708.886	10mM	DMSO

13018-09-2	Torilolone	252.35	10mM	DMSO
21764-41-0	Taxoquinone	332.44	10mM	DMSO
19913-01-0	Futoenone	340.37	10mM	DMSO
226904-40-1	4-Hydroxy-1,10-secocadin-5-ene-1,10-dione	252.35	10mM	DMSO
129314-37-0	Licorisoflavan A	438.56	10mM	DMSO
5088-75-5	Neoliquiritin	418.39	10mM	DMSO
35738-25-1	3β-Acetoxy-11α,12α-epoxyoleanan-28,13β-olide	512.731	10mM	DMSO
477-33-8	Samidin	386.395	10mM	DMSO
20105-22-0	Ravenine	243.3	10mM	DMSO
38695-41-9	Preskimmianine	303.35	10mM	DMSO
852875-96-8	2,3-Dihydropodocarpusflavone A	554.5	10mM	DMSO
51095-47-7	Methyl p-hydroxyphenyllactate	196.2	10mM	DMSO
10176-71-3	Ladanein	314.29	10mM	DMSO
20137-37-5	Rotundic acid	488.7	10mM	DMSO
54656-47-2	1-O-Methyljatamanin D	212.242	10mM	DMSO
124960-89-0	Tectoroside	588.6	10mM	DMSO
64181-07-3	30-Oxolupeol	440.7	10mM	DMSO
97856-19-4	8,9-Didehydro-7-hydroxydolichodial	180.203	10mM	DMSO
6426-44-4	Glutinol acetate	468.75	10mM	DMSO
38276-59-4	Burchellin	340.37	10mM	DMSO
13323-67-6	4-O-Methylbutein	286.28	10mM	DMSO
19685-09-7	10-Hydroxycamptothecin	364.36	10mM	DMSO
19891-85-1	29-Nor-20-oxolupeol	428.69	10mM	DMSO
30508-27-1	Licoricidin	424.53	10mM	DMSO
34292-87-0	2,3-Dihydrohinokiflavone	540.47	10mM	DMSO
910856-25-6	Danshenol C	336.38	10mM	DMSO
93395-31-4	8β-(2-Hydroxy-2-methyl-3-oxobutyryloxy)glucozaluzanin C	538.54	10mM	DMSO
148-03-8	β-Tocopherol	416.68	10mM	DMSO
24144-61-4	Khellactone	262.26	10mM	DMSO
64190-94-9	Zanthobungeanine	271.31	10mM	DMSO
121747-89-5	Isoderrone	336.34	10mM	DMSO
108885-61-6	Lupeol 3-hydroxyoctadecanoate	709.18	10mM	DMSO
2111-46-8	Marsformoxide B	482.74	10mM	DMSO
4263-88-1	(±)-Pinoresinol	358.39	10mM	DMSO
70360-12-2	Sideritoflavone	360.31	10mM	DMSO
28957-04-2	Oridonin	364.43	10mM	DMSO
62499-71-2	Isodihydrofutoquinol B	356.41	10mM	DMSO
62560-95-6	Isodihydrofutoquinol A	356.41	10mM	DMSO
60008-03-9	Glabrene	322.35	10mM	DMSO
64199-78-6	Barbinervic acid	488.7	10mM	DMSO
518-82-1	Emodin	270.2	10mM	DMSO
478-43-3	Rhein	284.21	10mM	DMSO
1260-17-9	Carminic acid	492.39	10mM	DMSO
548-04-9	Hypericin	504.45	10mM	DMSO
123940-54-5	Hypocrellin B	546.5	10mM	DMSO
521-61-9	Physcion	284.3	10mM	DMSO
477-84-9	Damnacanthal	282.3	10mM	DMSO
129393-21-1	Physcion 8-O-rutinoside	592.55	10mM	DMSO
23451-01-6	Physcion 8-O-beta-D-monoglucoside	446.4	10mM	DMSO
81-27-6	Sennoside A	862.74	10mM	DMSO
117-10-2	Dantron	240.2	10mM	DMSO
6268-09-3	1-Hydroxy-2-methylanthraquinone	238.2	10mM	DMSO
19852-76-7	9,10-Anthracenedione	284.3	10mM	DMSO
77029-83-5	Hypocrellin A	546.5	10mM	DMSO
1390-65-4	Carmine	492.39	10mM	DMSO
1415-73-2	Aloin A	418.39	10mM	DMSO
128-57-4	Sennoside B	862.74	10mM	DMSO
67979-25-3	Aurantio-obtusin	330.29	10mM	DMSO

481-74-3	Chrysophanol	254.2	10mM	DMSO
481-72-1	Aloeemodin	270.2	10mM	DMSO
87686-86-0	6-Hydroxyrubiadin	270.2	10mM	DMSO
7460-43-7	Rubiadin 1-methyl ether	268.3	10mM	DMSO
84-54-8	Tectoquinone	222.2	10mM	DMSO
72-48-0	Alizarin	240.2	10mM	DMSO
81-54-9	Purpurin	256.2	10mM	DMSO
117-78-2	2-Anthraquinonecarboxylic acid	252.2	10mM	DMSO
28371-16-6	Aloin B	418.39	10mM	DMSO
477-85-0	Obtusifolin	284.27	10mM	DMSO
13739-02-1	Diacerein	368.29	10mM	DMSO
37271-17-3	Sennoside D	848.76	10mM	DMSO
37271-16-2	Sennoside C	848.76	10mM	DMSO
17526-15-7	Xanthorin	300.3	10mM	DMSO
17241-40-6	2-Hydroxy-3-methylanthraquinone	238.2	10mM	DMSO
517-44-2	Sennidin B	538.46	10mM	DMSO
641-12-3	Sennidin A	538.46	10mM	DMSO
113443-70-2	Rhein-8-glucoside calcium salt	484.43	10mM	DMSO
55954-61-5	Pseudohypericin	520.44	10mM	DMSO
23313-21-5	Emodin-8-beta-D-glucoside	432.41	10mM	DMSO
13241-28-6	Chrysophanol 8-O-glucoside	416.4	10mM	DMSO
117-02-2	Rubiadin	254.2	10mM	DMSO
481-73-2	Citreorosein	286.2	10mM	DMSO
118555-84-3	Floribundone 1	566.52	10mM	DMSO
165689-32-7	Torososide A	728.66	10mM	DMSO
942609-65-6	1,5,15-Tri-O-methylmorindol	328.3	10mM	DMSO
518-83-2	Xanthopurpurin	240.21	10mM	DMSO
33037-46-6	Aloe-emodin-8-O-beta-D-glucopyranoside	432.4	10mM	DMSO
84-33-3	Ophiohayatone C	268.2	10mM	DMSO
6003-11-8	Alizarin 2-methyl ether	254.2	10mM	DMSO
602-63-1	1,2-dihydroxy-3-methyl-anthracene-9,10-dione	254.2	10mM	DMSO
24094-45-9	Digiferruginol	254.2	10mM	DMSO
149457-83-0	Hypocrellin C	528.5	10mM	DMSO
68243-30-1	2-Hydroxy-3-(hydroxymethyl)anthraquinone	254.2	10mM	DMSO
6170-06-5	2-Hydroxy-1-methoxyanthraquinone	254.2	10mM	DMSO
17241-59-7	2-(Hydroxymethyl)anthraquinone	238.2	10mM	DMSO
477-86-1	Digitolutein	268.3	10mM	DMSO
54328-09-5	Protopseudohypericin	522.46	10mM	DMSO
644967-44-2	Rubianthraquinone	284.26	10mM	DMSO
103392-51-4	1-O-Methylnataloe-emodin	284.26	10mM	DMSO
35688-09-6	Questinol	300.26	10mM	DMSO
5803-62-3	Averantin	372.37	10mM	DMSO
3774-64-9	Questin	284.26	10mM	DMSO
8015-61-0	Aloin	418.39	10mM	DMSO
478-08-0	Lucidin	270.05	10mM	DMSO
80368-74-7	3-hydroxymorindone	286.24	10mM	DMSO
14597-16-1	Laccaic acid E	495.4	10mM	DMSO
38840-23-2	Emodin 1-O-beta-D-glucoside	432.38	10mM	DMSO
1401414-53-6	1-Methyl-2,8-dihydroxy-3-carboxy-9,10-anthraquinone	298.3	10mM	DMSO
66466-22-6	Emodin-8-O-beta-gentiobioside	594.5	10mM	DMSO
120181-08-0	Chrysophanol 1-O-beta-tetraglucoside	902.8	10mM	DMSO
849789-95-3	Emodin-1-O-beta-gentiobioside	594.5	10mM	DMSO
